# Asymmetric Enantio-complementary Synthesis of Thioethers via Ene-Reductase-Catalyzed C–C Bond Formation

**DOI:** 10.1021/jacs.5c00761

**Published:** 2025-04-02

**Authors:** Christian M. Heckmann, Derren J. Heyes, Martin Pabst, Edwin Otten, Nigel S. Scrutton, Caroline E. Paul

**Affiliations:** † Department of Biotechnology, 2860 Delft University of Technology, van der Maasweg 9, Delft 2629HZ, The Netherlands; ‡ Manchester Institute of Biotechnology and Department of Chemistry, 5292 University of Manchester, 131 Princess Street, Manchester M1 7DN, U.K.; § Stratingh Institute for Chemistry, University of Groningen, Nijenborgh 3, Groningen 9747AG, The Netherlands

## Abstract

Enzymes are attractive catalysts due to their high chemo-, regio-, and enantioselectivity. In recent years, the application of enzymes in organic synthesis has expanded dramatically, especially for the synthesis of chiral alcohols and amines, two very important functional groups found in many active pharmaceutical ingredients (APIs). Indeed, many elegant routes employing such compounds have been described by industry. Yet, for the synthesis of chiral thiols and thioethers, likewise found in APIs albeit less ubiquitous, only very few biocatalytic syntheses have been reported, and stereocontrol has proved challenging. Here, we apply ene-reductases (EREDs), whose ability to initiate and control chemically challenging radical chemistries has recently emerged, to the synthesis of chiral thioethers from α-bromoacetophenones and pro-chiral vinyl sulfides, without requiring light. Depending on the choice of ERED either enantiomer of the product could be accessed. The highest conversion and selectivity were achieved with GluER T36A using fluorinated substrates, reaching up to 82% conversion and >99.5% *ee*. With α-bromoacetophenone and α-(methylthio)­styrene, the reaction could be performed on a 100 mg scale, affording the product in a 46% isolated yield with a 93% *ee*. Finally, mechanistic studies were carried out using stopped-flow spectroscopy and protein mass spectrometry, providing insight into the preference of the enzyme for the intermolecular reaction. This work paves the way for new routes for the synthesis of thioether-containing compounds.

## Graphical abstract

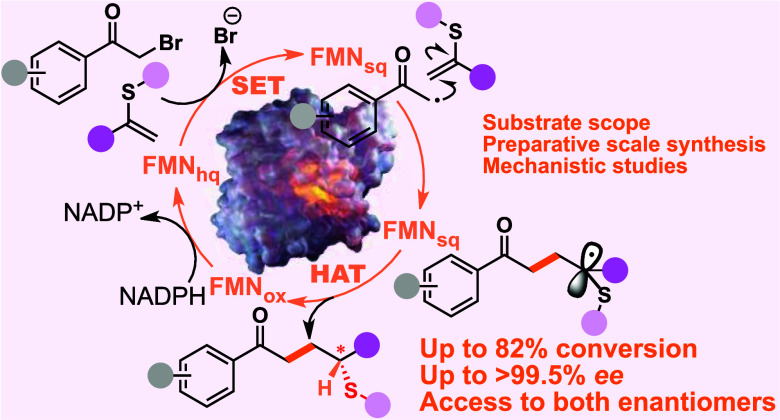

## Introduction

The efficient synthesis of chiral compounds is a key requirement for the synthesis of active pharmaceutical ingredients (APIs).^1^ Enzymes have a unique advantage over conventional chemistries, due to their innate high stereo-, regio-, and chemo-selectivities, and have emerged as a powerful tool in synthetic routes, as highlighted by recent examples from industry such as Merck’s routes to islatravir^2^ and nemtabrutinib,^3^ Pfizer’s route to abrocitinib,^4^ or GSK’s route to GSK2879552.^5^ Many enzyme classes have been employed in the synthesis of chiral alcohols and amines (Figure 1A),^6^ two key functional groups in many APIs; however, the same is not true for other important functional groups, such as thiols and thioethers (Figure 1B). Indeed, only few examples exist for the biocatalytic synthesis of thiols and thioethers (Figure 1C), most of which start from racemic compounds and employ (dynamic) kinetic resolutions.^7–10^ The first biocatalytic asymmetric synthesis of thioethers reaching high levels of enantioselectivity was recently reported by Zhao et al.^11^ The synthesis of thiols and thioethers by chemical means, such as metal catalysis, is also challenging due to the poisoning effect of sulfur on many metal catalysts.^12^ Furthermore, the reactivity of thiols further complicates enantiocontrol due to uncatalyzed background reactivity.

**Figure 1:**
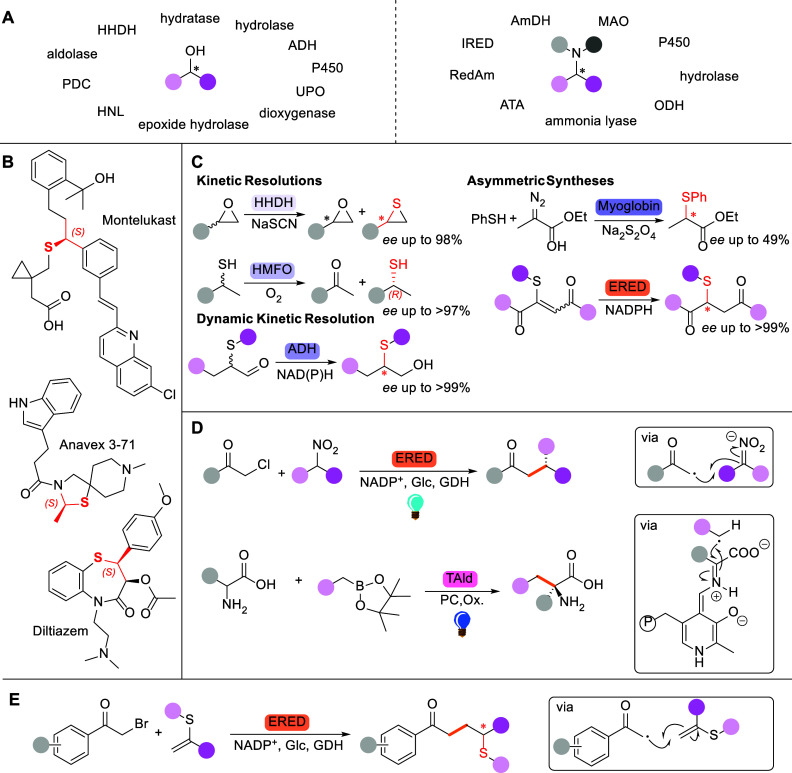
(A) Chiral alcohols and chiral amines and the main enzyme classes that have been applied in biocatalytic approaches for their production. ADH: alcohol dehydrogenase, PDC: pyruvate decarboxylase, UPO: unspecific peroxygenase, HHDH: halohydrin dehalogenase, HNL: hydroxynitrile lyase, IRED: imine reductase, AmDH: amine dehydrogenase, ATA: amine transaminase, RedAm: reductive aminase, ODH: opine dehydrogenase, MAO: monoamine oxidase. (B) Examples of APIs containing chiral thioethers. (C) Examples of chiral thiols and thioethers generated biocatalytically. HMFO: hydroxymethylfurfural oxidase, ERED: ene reductase. (D) Selected examples of recently developed enzymatically controlled radical reactions. GDH: glucose dehydrogenase, TAld: threonine aldolase, PC: photocatalyst, Ox.: oxidant. (E) Proposed synthesis of chiral thioethers from pro-chiral vinyl sulfides.

While some natural products contain chiral thiols and thioethers, the strategy for their synthesis in nature either relies on the asymmetric enzymatic synthesis of other functional groups (such as epoxides) followed by substitution or the use of radical enzymes.^13^ These enzymes are typically very substrate-specific and thus do not appear promising as general biocatalysts in a synthetic organic chemistry context. Recently, several classes of enzymes have been shown to be able to control non-native radical chemistries, such as ene-reductases (EREDs) by the Hyster group,^14^ pyridoxal 5-phosphate (PLP)-dependent enzymes by the Yang group (Figure 1D),^15^ and other examples.^16–23^ These strategies typically rely on light to generate radicals, either by accessing excited states of cofactors (e.g., flavin mononucleotide (FMN) in EREDs) or by supplying an exogenous photocatalyst. This light requirement comes with serious disadvantages, such as known photodegradation of the light-sensing chromophore,^24,25^ as well as light penetration during process scale-up/intensification.^26^

Remarkably, some EREDs do not require light to access single-electron chemistries with α-bromoacetophenones, generating reactive α-acyl radicals that have been shown to react with styrenes.^27^ In the case of pro-chiral styrenes, such as α-methylstyrene, the benzylic radical intermediate formed after radical addition to the double bond is terminated via a highly enantioselective hydrogen atom transfer (HAT) from the FMN cofactor; however, larger substituents than methyl or ethyl have been reported to give only traces of conversion.^27^ Inspired by this reaction, we envisaged that radical addition to prochiral vinyl sulfides, followed by enantioselective HAT would generate chiral thioethers (Figure 1E). This strategy requires the enzyme to accept the large size of the sulfur substituent at the double bond and control both the reactive α-acyl radical intermediate as well as the orientation of the cosubstrate to prevent unselective radical chemistries occurring at the sulfur.^28^

## Results and Discussion

In our initial attempts of the reaction between α-bromoacetophenone **1a** and α-(methylthio)­styrene **2a**, using a variant of the ERED from Gluconobacter oxydans (GluER T36A),^29^ we were pleased to observe the formation of predominantly a single product by HPLC (Table 1, **entry 1**; Figures S2 and S8); however, this product appeared more hydrophilic than expected. Analyzing the reaction further by GC–MS, we observed two products that were identified as α-(methylthio)­acetophenone **6a** and a second product with a mass and fragments consistent with **7a** (Figure S42). Expecting a competing dehalogenation of substrate **1a** to give acetophenone **4a**, we used an excess of **1a**. We speculated that product **3aa** was indeed formed (Scheme 1), but sufficiently nucleophilic to react with a second equivalent of substrate **1a**, forming sulfonium **5aa** (observed by HPLC). Upon heating during GC–MS injection, **5aa** eliminates **6a**, thus forming the two observed products. Indeed, incubating isolated **3aa** with **1a** under the reaction conditions (no ERED), we observed the formation of the same products (Figures S8 and S43). Reducing the equivalents of **1a** (Table 1, **entries 2–4;** Figures S2, S9, and S44), the undesired product peak(s) decreased and the desired product **3aa** was the predominant product formed. Using equal amounts of **1a** and **2a** proved to give the highest conversions to product **3aa**. We also explored analogues of **1a** bearing weaker leaving groups, namely, α-chloroacetophenone (Cl-**1a**) and α-fluoroacetophenone (F-**1a**), and while indeed the undesired product was no longer observed (Figures S2 and 11), the formation of **3aa** was also diminished or fully abolished, respectively (Table 1, **entries 5–6**).

**Table 1:**
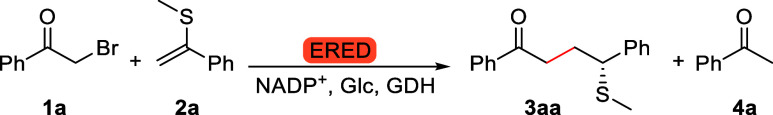
Exploration of Reaction Conditions

Entry	ERED	[1a] (mM)	[2a] (mM)	conversion to 3aa (%)	ee 3aa (%)
1	GluER T36A	30	≈10^a^	2	n.d
2	GluER T36A	15	10	74	86 (*R*)
3	GluER T36A	10	10	76	99 (*R*)
4	GluER T36A	10	15	64	97 (*R*)
5	GluER T36A	10 (Cl-**1a**)	≈30^a^	25	n.d
6	GluER T36A	10 (F-**1a**)	≈30^a^	0	n.d
7^b^	GluER T36A	10	10	0	n.d
8	GluER T36A^c^	10	10	45	80 (*R*)
9	GluER T36A^d^	10	10	75	>99.5 (*R*)^g^
10^e^	GluER T36A	20	20	74	99 (*R*)
11^e^	GluER T36A^f^	20	20	64	86 (*R*)
12	FMN	10	10	0	n.d
13	NCR	10	10	45	70 (*R*)
14	OYE3	10	10	0	n.d
15	PETNR	10	10	40	87 (*S*)

a Partially polymerized/oxidized **2a**.

b Aerobic.

c 0.8 mol %.

d 2.0 mol %.

e d-glucose (100 mM), Tris-HBr (100 mM).

f 0.7 mol %.

g Other enantiomer not detected; n.d.: not determined. Conditions: d-glucose (55 mM), NADP^+^ (0.5 mM), JM GDH-101 (0.5 mg mL^–1^), **1a** (10–30 mM), **2a** (10–30 mM), ERED (1.4 mol %), Tris-HBr (50 mM), pH 7.5, 25 °C, 750 rpm, anoxic, 24 h.

**Scheme 1:**
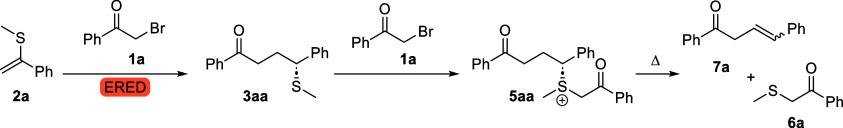
Proposed Side Product Formation

Next, we explored the catalyst loading and found that decreasing the catalyst loading resulted in both lower conversion and *ee*, while increasing the loading increased both slightly (Table 1, **entries 8–9,** Figure S2). Doubling the substrate concentration, we observed comparable conversion and enantioselectivity (Table 1, **entry 10,** Figure S2). Again, using a lower catalyst loading reduced both conversion and *ee*, albeit to a lesser extent than at the lower substrate concentration (Table 1, **entry 11**, Figure S2).

We carried out a time course of the reaction, which showed that the reaction reached the final conversion after between 16 and 24 h. We also observed small amounts of putative **5aa** forming transiently (Figures 2 and S12). We hypothesized that the reaction shown in Scheme 1 was reversible, leading to racemization of **3aa** over time; however, we observed no loss in *ee* when incubating isolated **3aa** with **1a** (Figure S30). Thus, we are currently unable to explain the decrease in *ee* for slower reactions.

**Figure 2:**
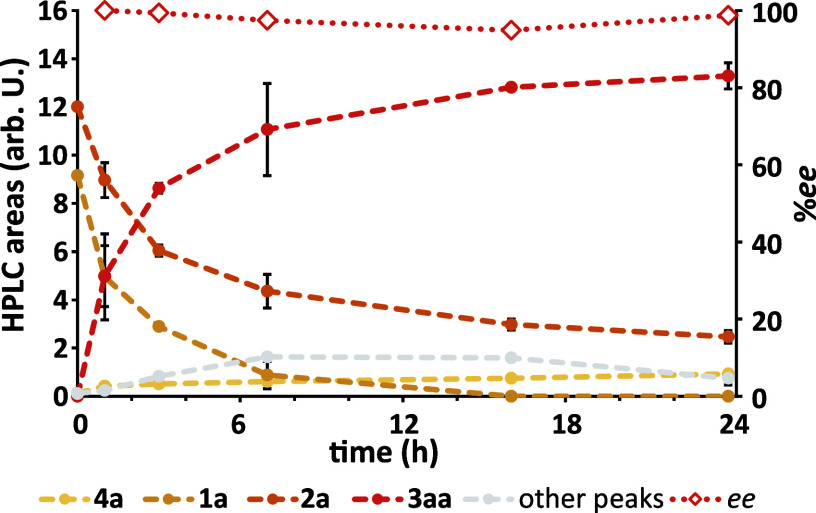
Time course of the GluER T36A-catalyzed reaction between **1a** and **2a**. Conditions: d-glucose (55 mM), NADP^+^ (0.5 mM), JM GDH-101 (0.5 mg mL^–1^), **1a**–**d** (10 mM), **2a** (10 mM), ERED (1.4 mol %), Tris-HBr (50 mM), pH 7.5, 25 °C, 750 rpm, anoxic. Independent reactions for each time point. Time points are averages; error bars are standard deviations (*n* = 2).

Having identified suitable reaction conditions with GluER T36A, we looked at a small panel of the EREDs. Both NCR from *Zymomonas mobilis* ^30^ and PETNR from Enterobacter cloacae^31^ showed conversion to the desired product, while OYE3 from Saccharomyces cerevisiae^32^ showed no product formation (Table 1, **entries 13–15,** Figure S3). Like GluER T36A, NCR selectively formed the (*R*)-enantiomer but with lower conversions and *ee*, while PETNR formed the opposite (*S*)-enantiomer albeit with lower *ee*. Control reactions with FMN, or with GluER T36A in the presence of oxygen, showed no conversion, with the latter showing a variety of unidentified side products (Table 1, **entries 7** and **12,** Figure S3).

We then explored the substrate scope of the reaction, exploring substituents on both the vinyl sulfide and α-bromoacetophenone (Figures 3 and S3). In each case, GluER T36A and PETNR were the best sets of enantiocomplementary enzymes. A bulky naphthyl group (**2b**) resulted in decreased conversions for all EREDs and decreased enantioselectivity with GluER T36A, whereas enantioselectivity improved with PETNR. Methyl substituents were tested at the *para*-, *meta*-, and *ortho*-position (**2c**–**e**). Substitution at the *ortho*-position was poorly tolerated, resulting in substantially decreased conversions and increased side-product formation (which GC–MS suggests is predominantly the corresponding diketone (Figure S49)^33^), whereas *para*-substitution slightly reduced conversion. In both cases, *ee* was decreased. Substitution at the *meta*-position was tolerated best, obtaining high *ee*s with both GluER T36A and PETNR, with the latter showing a slightly reduced conversion. An electron-withdrawing *para*-fluoro substituent (**2f**) boosted conversions and *ee* for GluER T36A, while the effect on PETNR was negligible. While we would have liked to test an electron-donating *para*-methoxy substituent, the compound proved to be too unstable to be synthesized. Unexpectedly, phenyl vinyl sulfide (**2g**) was also accepted, while in the absence of the sulfur, an aromatic group is required to stabilize the second radical intermediate prior to termination by HAT.^27^ Pro-chiral analogue **3h** showed modest conversion with both GluER T36A and PETNR, with modest to weak enantioselectivity, respectively, and both enzymes forming the same enantiomer, indicating a change in binding mode compared to that of styrenyl substrates **2a**–**f**.

**Figure 3:**
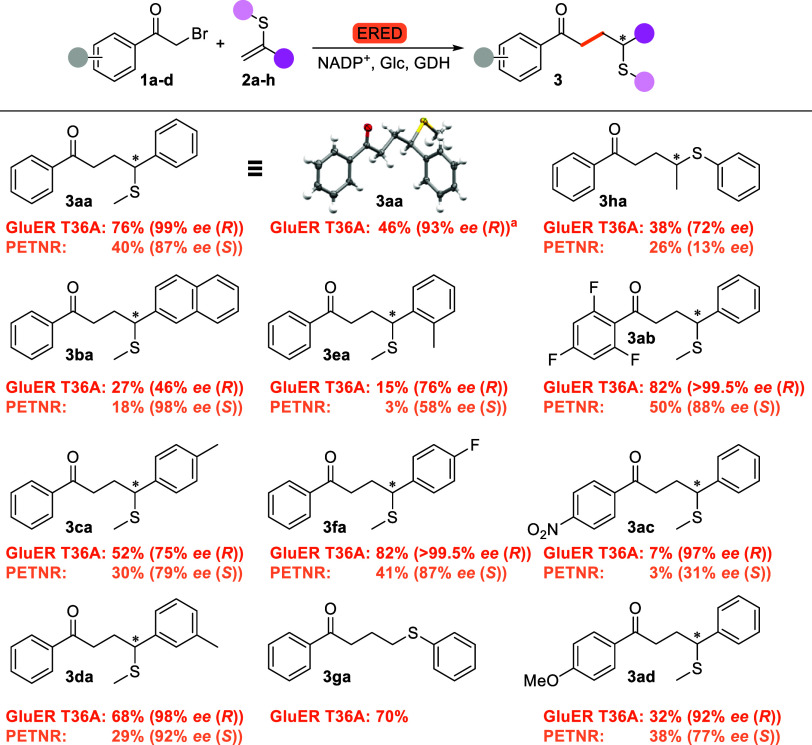
Substrate scope. Conversions based on relative HPLC areas. Conditions: d-glucose (55 mM), NADP^+^ (0.5 mM), JM GDH-101 (0.5 mg mL^–1^), **1a**–**d** (10 mM), **2a**–**h** (10 mM), ERED (1.4 mol %), Tris-HBr (50 mM), pH 7.5, 25 °C, 750 rpm, anoxic, 24 h. ^a^ isolated yield (49.5 mg), d-glucose (100 mM), NADP^+^ (0.5 mM), JM GDH-101 (0.5 mg mL^–1^), **1a** (20 mM), **2a** (20 mM), ERED (1.0 mol %), Tris-HBr (100 mM), pH 7.5, 23–24 °C, anoxic, 24 h. (*R*)-**3aa**: CCDC 2410188.

On the acetophenone side, 2,4,6-trifluoro-substituted **1b** showed improved conversions with both EREDs and boosted *ee* in the case of GluER T36A. Yet, an electron-withdrawing *para*-nitro group (**2c**) resulted in very low conversions with both EREDs. An electron-donating *para*-methoxy group (**2d**) reduced conversion only for GluER T36A. This suggests that both EREDs are affected to different extents by both steric and electronic effects.

To investigate the nature of the enantiocomplementarity of GluER T36A and PETNR, we docked the prochiral benzylic radical intermediate **3aa** ^•^ into the active sites of GluER T36A (PDB: 6MYW) and PETNR (1GVO).^29,34^ For both enzymes, we found two similar binding poses, leading to both enantiomers (Figure S4). Measuring the distance between the benzylic carbon and N5 of the flavin, we see that for GluER T36A, the pro-(*R*) binding mode shows a shorter distance of 3.4 Å vs 4.0 Å for the pro-(*S*) binding mode, explaining the high enantioselectivity. On the other hand, in PETNR, the distance for the pro-(*S*) binding mode is 4.1 Å vs 4.6 Å for the pro-(*R*) binding mode. These relative distances are consistent with the observed selectivities, and the larger distances observed in PETNR might explain the lower conversions observed with this enzyme. For the pro-(*R*) binding mode, binding of the substrate predominantly occurs via π–π interactions with the isoalloxazine moiety of FMN. The relative bulkiness of F269 in GluER T36A vs L275 in PETNR appears to shift the position of the bound intermediate, affecting the catalytic distance. In PETNR, the pro-(*S*) binding mode is rotated and stabilized by an additional π–π interaction with H184, and a hydrogen bond with Y351, compared with GluER T36A.

We were surprised by the high selectivity of GluER T36A for the formation of **3aa** over that of **4a** (Figure 2). For photobiocatalytic reactions, it has been shown that the presence of a cosubstrate can enhance the absorbance of the charge transfer complex, thus providing a gated mechanism toward the intermolecular reaction over competing dehalogenation.^35^ A gating mechanism has also been suggested for a nonlight-dependent reaction^27^ but has not been directly shown. To investigate this hypothesis (Figure 4A), we carried out presteady-state kinetics by stopped flow with a diode array detector, to follow the reoxidation of flavin hydroquinone (FMN_hq_) by **1a** in the presence or absence of **2a**. We observed FMN_hq_ and FMN_ox_ but not the FMN semiquinone (FMN_sq_, Figure S5), indicating that the initial single electron transfer (SET) is much faster than the subsequent radical reactions and the final hydrogen atom transfer (HAT). Thus, the stopped-flow data was most appropriately modeled as a single step from FMN_hq_ to FMN_ox_ (Figure 4B).

**Figure 4:**
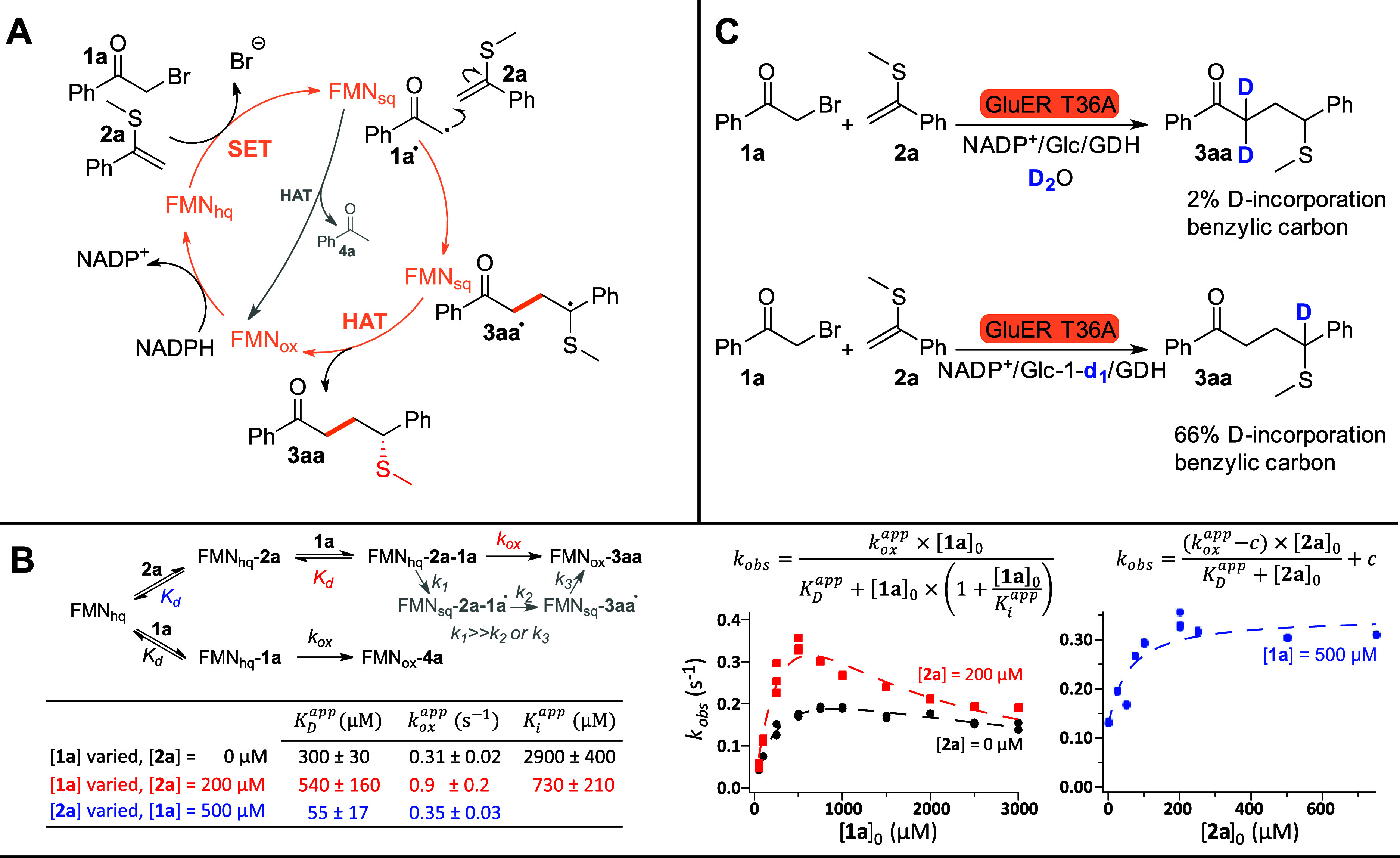
Mechanistic investigations. (A) Proposed catalytic cycle. SET: single electron transfer. HAT: hydrogen atom transfer. (B) Presteady state kinetics measured by stopped-flow. No signal for the semiquinone was observed (see Figure S5), implying that the SET is the rate-determining step. The reaction was thus modeled as a single step (rate constant *k*_ox_). An acceleration of the reaction was observed in the presence of the vinyl cosubstrate (**2a**). The affinity for **2a** was 1 order of magnitude higher compared to **1a**, explaining the exquisite selectivity of the enzyme for the formation of **3aa**. (C) Deuterium incorporation experiments, showing that the hydrogen at the benzylic position originates from the FMN cofactor.

The stopped-flow experiments confirmed a rate enhancement of the SET in the presence of cosubstrate **2a**. Furthermore, the affinity for **2a** is an order of magnitude higher than that for substrate **1a**. This suggests that the highly reactive α-acyl radical is predominantly formed in the presence of **2a**, demonstrating a gating mechanism, where the α-acyl radical is predisposed for C–C bond formation. Deuterium labeling experiments confirm that the benzylic hydrogen in **3aa** comes from the FMN cofactor, as expected (Figure 4C).

We next investigated the reaction without a cosubstrate in more detail. As expected, no reaction was observed with only cosubstrate **2a**. Interestingly, the concentration of acetophenone **4a** formed in the absence of the cosubstrate was only slightly increased compared to the reaction containing both substrates and a significant loss of material was observed (Figure 5A). This implies that, in the absence of the cosubstrate, the fate of the α-acyl radical is not predominantly acetophenone **4a**. Concurrently, we observed a yellow color, indicative of oxidized flavin, in the reaction containing only **1a** (Figure 5A,B), implying enzyme inactivation. We carried out protein mass spectrometry on the band corresponding to GluER T36A (Figure 5C) and observed a decrease in the abundance of certain peptides containing active-site amino acids (Figure 5D) that may become modified by highly reactive radicals in the active site (Figure 5E). Reactions containing either only **2a** or both substrates showed no such decrease (Figures S6 and S7), demonstrating that the reaction with the cosubstrate outcompetes this radical inactivation. While we observed a clear decrease in peptide abundance, we were unable to determine the nature of the modifications. We also observed a potential addition of acetophenone to residue C97 (with concurrent proton loss), but this modification is difficult to rationalize as the side chain is buried and facing away from the active site of GluER T36A.

**Figure 5:**
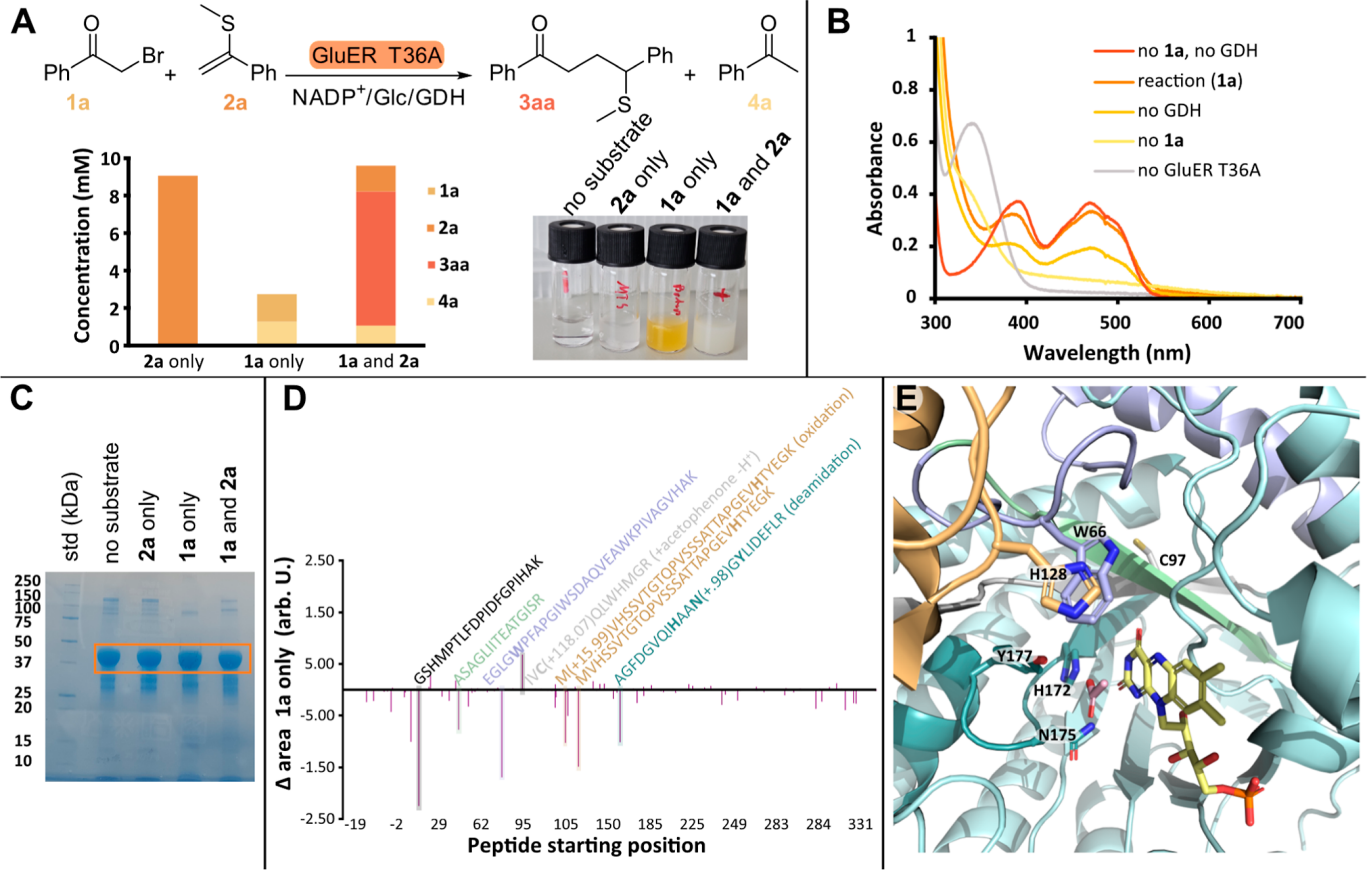
(A) Outcome of biotransformations with GluER T36A with **2a** only, **1a** only, or both **1a** and **2a**. Concentrations determined by HPLC using a calibration curve. It is apparent that in the absence of **2a**, the fate of the α-acyl intermediate is not predominantly **4a**. Also shown is the appearance of biotransformations with GluER T36A after 24 h with no substrate, **2a** only, **1a** only, or both **1a** and **2a**. The yellow color in the reaction with **1a** is indicative of oxidized flavin, implying enzyme inactivation. (B) UV–vis spectra of reactions containing **1a**, as well as controls without the substrate, GluER T36A, or GDH. The spectrum of FMN after a reaction with **1a** only matches that of enzyme-bound oxidized flavin (no substrate, no GDH control). The control containing **1a** but no GDH showed extensive precipitation of GluER T36A resulting in a lower flavin peak. In the absence of **1a**, FMN remains fully reduced, with some slight turbidity. (C) Coomassie-stained SDS-PAGE gel of biotransformations with GluER T36A after 24 h with no substrate, **2a** only, **1a** only, or both **1a** and **2a**. The orange box shows GluER T36A. (D) Difference in peptide abundance after tryptic digest of the band of GluER T36A in the reaction with **1a** only and without the substrate. For the difference in peptide abundance of **2a** only and **1a** and **2a**, see Figures S5 and S6. (E) Active site of GluER T36A (PDB: 6MYW) with acetate bound, showing peptides with decreased abundance by color, putative amino acids in the active site that may become modified by radical reactions, and C97 which is distal from the active site but may have formed an adduct with the α-acyl radical.

## Conclusions

We have successfully developed a novel biocatalytic strategy for the synthesis of chiral γ-thioether ketones. Depending on the choice of ERED, both enantiomers may be accessed with moderate to excellent *ee*. Mechanistic experiments showed a clear acceleration and the rate-determining SET in the presence of the vinyl sulfide cosubstrate, suggesting a gating mechanism which enables the highly selective C–C bond formation. Indeed, in the absence of the cosubstrate, the enzyme becomes inactivated with the FMN cofactor remaining in an oxidized state, which protein mass spectrometry suggests is due to unspecified modifications of protein by generated radicals. Finally, we demonstrated the synthetic applicability of the reaction on a 100 mg scale. We anticipate this reaction to be immediately useful in the development of compound libraries, for example, during drug discovery, whereas applications on a process scale require enzyme engineering and optimization of product isolation. Protein engineering may be informed by targeting the steric and electronic interactions identified by the docking studies and residues implicated in the radical damage. However, care must be taken to avoid modifying residues important for cofactor binding, such as the conserved His–His/Asn motif (H172 and N175 or H181 and H184 in GluER T36A and PETNR, respectively).^36^

## Materials and Methods

Chemicals were purchased from Sigma-Aldrich, abcr GmbH, Fisher, or TCI and used without further purification. Where specified, solvents were dried over 3 Å molecular sieves for at least 48 h. Biotransformations were carried out in a COY chamber, using degassed buffers and solvents. GDH-101 was provided by Johnson Matthey (JM). NMR spectra were recorded on an Agilent 400/54 premium shielded spectrometer: ^1^H-spectra referenced relative to TMS and ^13^C-spectra referenced using absolute referencing relative to the corresponding ^1^H-spectrum. EREDs were produced in E. coli BL21 Gold­(DE3) and purified by immobilized metal affinity chromatography, as previously described.^37^ Vinyl sulfides **2a** to **2g** were synthesized based on the literature.^38–40^ Further details can be found in the electronic Supporting Information.

### Biotransformation Reactions (0.5 mL Scale)

Reactions were set up in a COY chamber using degassed buffers and solvents that had been allowed to equilibrate in the COY chamber for at least 24 h. EREDs (<200 μL aliquots) were thawed in the COY ante-chamber over several vacuum cycles and allowed to equilibrate for approximately 15 min. Stock solutions were prepared in the COY chamber. To a 1.5 mL glass vial with a screw top and PTFE septum were added (in the following order) Tris-HBr (50 mM, pH 7.5, 182–325 μL), d-glucose (50 μL, 550 mM stock in Tris-HBr), NADP^+^ (50 μL, 5 mM stock in Tris-HBr), JM GDH-101 (25 μL, 10 mg/mL stock in Tris-HBr), styrene (25 μL, 200 mM stock in DMSO), α-bromoacetophenone (25 μL, 200 mM stock in DMSO), and ERED (0–143 μL, 490–1300 μM), to a final reaction volume of 500 μL. Biotransformations were incubated in an Eppendorf Thermomixer at 25 °C and 750 rpm for up to 24 h inside the COY chamber. Reactions were quenched aerobically by the addition of acetonitrile (500 μL). HPLC samples were prepared by adding 200 μL of the quenched reaction mixture to 800 μL of acetonitrile; the precipitated protein was removed by centrifugation (21,000*g*, 2 min). GC–MS samples were prepared by extracting the remaining quenched reaction mixture with 600 μL of EtOAc. Details on analytical methods and characterization of **3aa** and **3ha** can be found in the electronic Supporting Information.

### Preparative-Scale Biotransformation—Synthesis of **3aa**

A round-bottomed flask was charged with a magnetic stirrer, JM GDH-101 (10 mg), NADP^+^ disodium (7.8 mg, 9.91 μmol), d-glucose monohydrate (396.4, 2 mmol), and α-bromoacetophenone (79.6 mg, 400 μmol). The flask was then brought into a COY chamber, and Tris-HBr (15 mL, 100 mM, pH 7.5), DMSO (1 mL), and α-(methylthio)­styrene (1 mL of a 400 mM stock solution in DMSO) were added. Finally, GluER T36A (3.02 mL, 1300 μM stock, 3.92 μmol) was added, the flask was sealed with a rubber septum and wrapped in aluminum foil, and the reaction mixture was stirred (at a speed just below vortex formation) in the COY chamber for 26 h at ambient temperature (23–24 °C). Brine (10 mL) was added, and the reaction mixture was extracted with EtOAc (3 × 20 mL), breaking the emulsion with gentle heating when needed. The combined organic extracts were washed with brine (10 mL) and dried (MgSO_4_), and the solvent was removed in vacuo giving crude **3aa** (162.9 mg) as a pale-yellow very viscous oil, which was purified by preparative TLC (Supelco PLC Silica Gel 60 F_254_ (20 cm × 20 cm × 2 mm; 20 cm × 4 cm concentrating zone); pentane/diethyl ether 98:2; 4 passes), obtaining **3aa** (49.5 mg, 46% yield, 93% *ee*) as a clear oil which solidified over time. ^1^H NMR (400 MHz, CDCl_3_): δ 1.9 (3H, s), 2.23–2.42 (2H, m), 2.96–3.11 (2H, m), 3.80 (1H, dd, *J* 8.0, 7.2 Hz), 7.22–7.28 (1H, m), 7.33 (4H, app. d, *J* = 4.4 Hz), 7.40–7.46 (2H, m), 7.54 (1H, tt, *J* = 7.4, 1.4 Hz), 7.87–7.91 (2H, m); ^13^C­{^1^H}-NMR (101 MHz, CDCl_3_): δ 14.2 (CH_3_), 30.1 (CH_2_), 36.4 (CH_2_), 50.7 (CH), 127.2 (CH), 127.8 (CH), 128.0 (CH), 128.5 (CH), 128.6 (CH), 133.0 (CH), 136.8 (C), 141.9 (C), 199.4 (C).

## Supplementary Material



## Notes

Detailed materials and methods (enzyme production, synthesis of substrates, biotransformations, stopped-flow kinetics, crystallography, mass spectrometry, spectroscopy, dockings, and analytical methods); additional results, HPLC and GC–MS chromatograms, and NMR spectra (PDF)
