# Supplementary material for: Asymmetric Enantio-complementary Synthesis of Thioethers via Ene-Reductase-Catalyzed C–C Bond Formation
Source: J Am Chem Soc. 2025 Apr 2;147(22):18618–25. doi: 10.1021/jacs.5c00761 (PMC12147138; doi:10.1021/jacs.5c00761)

Electronic Supporting Information

Asymmetric Enantio-Complementary Synthesis of Thioethers via Ene Reductase Catalyzed C-C bond formation

Christian M. Heckmann,[a,]* Derren J. Heyes,[b] Martin Pabst,[a] Edwin Otten,[c] Nigel S. Scrutton,[b] Caroline E. Paul[a,]*

[a] Department of Biotechnology, Delft University of Technology, van der Maasweg 9, 2629HZ Delft, The Netherlands; c.m.heckmann@tudelft.nl; c.e.paul@tudelft.nl

[b] Manchester Institute of Biotechnology and Department of Chemistry, University of Manchester, 131 Princess Street, Manchester M1 7DN, UK

[c] Stratingh Institute for Chemistry, Nijenborgh 3, 9747AG, Groningen, The Netherlands

Contents

Materials and Methods 2
- Enzyme production 2
- Synthesis of α-(methylthio)styrenes 3
  - α-(methylthio)styrene **2a** (standard protocol) 3
  - 2-(1-methylthiovinyl)naphthalene **2b** 4
  - α-methylthio-4-methylstyrene **2c** 4
  - α-methylthio-3-methylstyrene **2d** 4
  - α-methylthio-2-methylstyrene **2e** 4
  - α-methylthio-4-fluorostyrene **2f** 4
- Synthesis of 2-(phenylthio)propene **2h** 5
- Biotransformation reactions (0.5 mL scale) 5
- Preparative scale biotransformation—synthesis of **3aa** 6
- Stopped-flow Kinetics 7
- X-ray Crystallography 7
- Protein Mass-spectrometry 9
- UV-vis spectroscopy of reactions without vinyl co-substrate 10
- Docking studies 10
- Analytical methods 10
  - Reverse phase HPLC 10
  - Chiral reverse phase HPLC 10
  - Chiral normal phase HPLC 11
  - GC-MS 11
- References 11

Supplementary Figures 12

DNA and protein sequences 17
- pET22b-NCR 17
- pET28a-GluER T36A 17
- pET21a-PETNR 18
- pET28a-OYE3 18

HPLC chromatograms 19

Chiral HPLC chromatograms 30

GC-MS chromatograms 38

NMR spectra 50

Materials and Methods

Chemicals were purchased from Sigma Aldrich, abcr GmbH, Fisher, or TCI and used without further purification. Where specified, solvents were dried over 3 Å molecular sieves for at least 48 h. Biotransformations were carried out in a COY chamber, using degassed buffers and solvents. GDH-101 was provided by Johnson Matthey (JM). NMR spectra were recorded on an Agilent 400/54 Premium Shielded Spectrometer; ^{1}H-spectra referenced relative to TMS, ^{13}C-spectra referenced using absolute referencing relative to the corresponding ^{1}H-spectrum.

Enzyme production

E. coli BL21 Gold(DE3) competent cells were transformed with a plasmid harbouring the gene of interest. An overnight pre-culture (LB, 15 mL, kanamycin (kan, 50 µg/mL) or ampicillin (amp, 100 µg/mL)) was inoculated with a single colony and grown overnight (37 °C, 180 rpm). TB medium (600 mL) containing lactose (5 g/L), glucose (0.5 g/L), and either kan (50 µg/mL) or amp (100 µg/mL) was inoculated with the pre-culture, and grown for 2-3 h at 37 °C, 170 rpm, and then 25 °C, 170 rpm for an additional 24 h (using 2 L baffled shake flasks and a 1 inch throw). Cells were harvested by centrifugation (4500 *g*, 4 °C, 20 min). Pellets were stored at -20 °C until purification.

Pellets were resuspended (with the aid of brief sonication) in buffer A (3 v/w, MOPS-NaOH (20 mM), NaCl (300 mM), imidazole (25 mM), pH 7.5), and FMN (approx. 0.2 mg/mL) and a spatula tip each of $MgCl_2$ and DNase were added. Cells were lysed using a cell disruptor (Multi Shot Cell Disruption System at 4 °C, 21 kPsi, 2 passes) and the lysate clarified by centrifugation (48,000 *g*, 4 °C, 45-60 min), and the cell-free extract filtered (0.45 µm followed by 0.2 µm). Protein purification was carried out on a Bio-Rad NGC system, using HisTrap FF crude nickel columns (5 mL, up to two columns in series). After sample loading, the column was washed with buffer A (2-4 CV, depending on the level of leaching of the EREDs that was observed), and eluted with 100% buffer B (identical to buffer A, but imidazole (300 mM)). Fractions containing purified ERED were pooled and dialyzed against storage buffer (MOPS-NaOH (20 mM), pH 7.5) overnight (the buffer was replaced after the first 3 h). Were needed, EREDs were concentrated using AMICON Ultra-15 (10 kDa MWCO) filters to 500-1300 µM. Protein concentration was determined using the enzyme bound FMN (GluER T36A:[1] ε_{464}=11400 $M^{-1}cm^{-1}$, NCR:[2] ε_{464}=10500 $M^{-1}cm^{-1}$, OYE3: ε_{464}=10850 $M^{-1}cm^{-1}$, PETNR: ε_{467}=10500 $M^{-1}cm^{-1}$). For OYE3 and PETNR, extinction coefficients were determined by denaturing the enzyme using SDS and measuring the concentration of released FMN (ε_{446}=12200 $M^{-1}cm^{-1}$).[3]

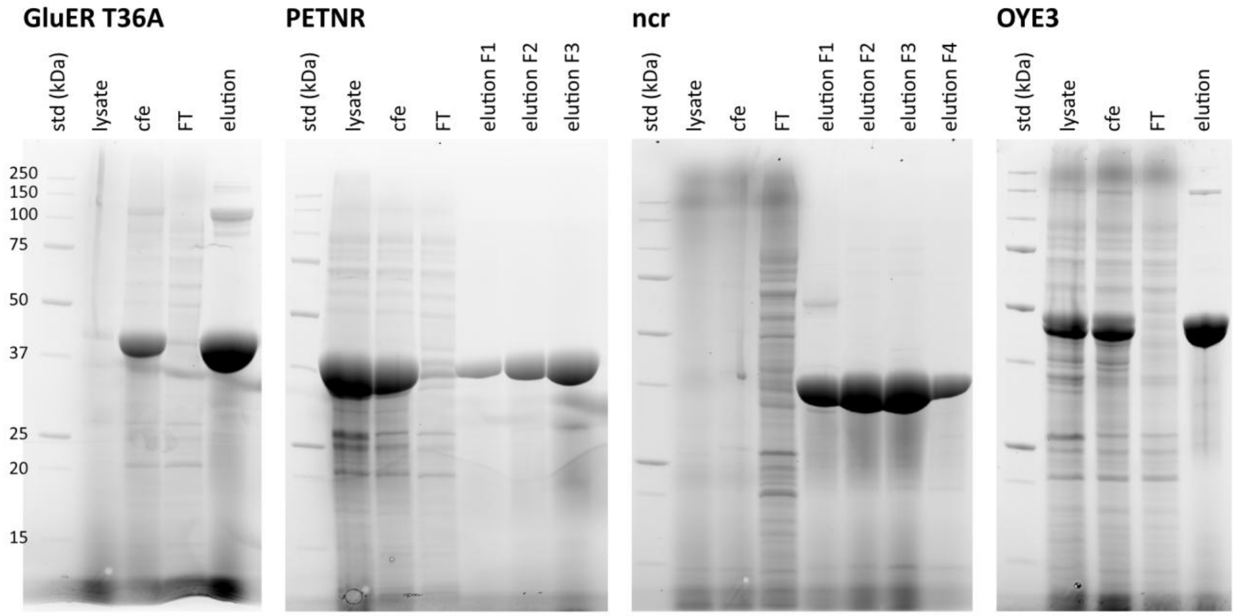

Figure S1: SDS-PAGE gels of the EREDs used in this study. std: protein standard, cfe: cell-free-extract, FT: flow-through, F1-4: fractions 1-4. Protein ladder: Precision Plus Protein™ Unstained Protein Standard from BioRad.

Synthesis of α-(methylthio)styrenes

α-(Methylthio)styrenes **2a** to **2f** were synthesized based on literature.[4,5] Products were stored under nitrogen at -20 °C.

*α-(methylthio)styrene **2a** (standard protocol)*

To a Schlenk flask was added a magnetic stirrer and bromodimethylsulfonium bromide (BDMS.Br; 555 mg; 2.50 mmol), and the flask was evacuated and backfilled with nitrogen (3 cycles), and cooled on ice. Dry acetonitrile (2 mL) was added, followed by dropwise addition of styrene (290 µL, 2.56 mmol) to the stirred mixture. After addition was complete, the mixture was stirred on ice for an additional 15 min, the precipitated intermediate was obtained by vacuum filtration, washed with a minimal amount of cold acetonitrile, and dried with suction. The intermediate (335 mg, 1.03 mmol) was transferred to a Schlenk flask charged with a stirrer bar, which was then evacuated and back-filled with nitrogen (3 cycles) and cooled on ice. Dry dichloromethane (5 mL) was added, followed by dropwise addition of dry triethylamine (430 µL, 3.09 mmol, 3 equivalents) to the stirred suspension. The reaction mixture was then brought to ambient temperature, and stirred for 1 h. Water (5 mL) was added to the solution, as well as tert-butylcatechol (2 µg, from a 0.1 mg/mL stock solution). The mixture was carefully acidified using hydrochloric acid (6M) to a pH of approximately 5, and the organic layer washed with water (2×5 mL), dried ($MgSO_4$), and the solvent removed under a stream of nitrogen. After final drying under vacuum at ambient temperature, (α-methylthio)styrene was obtained as a slightly turbid pale yellow oil (147.2 mg, 0.98 mmol, 98% purity (GC-MS), 39% yield over two steps). ^{1}H-NMR (400 MHz, $CDCl_3$) δ 2.32 (3 H, s), 4.97 (1 H, s), 5.44 (1 H, s), 7.29–7.38z (3 H, m), 7.51–7.56 (2 H, m); ^{13}C{^{1}H}-NMR (101 MHz, $CDCl_3$) δ 15.6 (CH_3), 107.3 (CH_2), 126.9 (CH), 128.3 (CH), 128.4 (CH), 139.7 (C), 146.5 (C). HRMS: m/z $[M+H]^+$ calc. 151.0576, found: 151.0575.

2-(1-methylthiovinyl)naphthalene ***2b***

For the second step, using 332 mg (0.883 mmol) of the intermediate, additional triethyl amine (470 µL, 3.38 mmol, 3.8 eq.) and acetonitrile (0.5 mL) as co-solvent were employed. The reaction was stirred for 2.5 h. 2-(1-methylthiovinyl) was obtained as a turbid viscous pale yellow oil (98.2 mg, 0.490 mmol, 96% purity (GC-MS) 23% yield over two steps). ^{1}H-NMR (400 MHz, $CDCl_3$) δ 2.36 (3 H, s), 5.09 (1 H, s), 5.58 (1 H, s), 7.44–7.54 (2 H, m), 7.67 (1 H, dd, *J* 8.5, 1.8 Hz), 7.79–7.90 (3 H, m),8.02 (1 H, d, *J* 1.8 Hz); ^{13}C{^{1}H}-NMR (101 MHz, $CDCl_3$) δ 15.7 (CH_3), 108.1 (CH_2), 125.0 (CH), 125.9 (CH), 126.2 (CH), 126.3 (CH), 127.6 (CH), 128.0 (CH), 128.3 (CH), 133.2 (C), 133.3 (C), 137.0 (C), 146.4 (C). HRMS: m/z $[M+H]^+$ calc. 201.0733, found: 201.0730.

α-methylthio-4-methylstyrene ***2c***

Obtained as a slightly turbid almost colourless oil (136 mg, 0.828 mmol, 95% purity (GC-MS) 33% yield over two steps). ^{1}H-NMR (400 MHz, $CDCl_3$) δ 2.31 (3 H, s), 3.35 (3 H, s), 4.93 (1 H, s), 5.41 (1 H, s), 7.15 (2 H, d, *J* 7.9 Hz), 7.43 (2 H, d, *J* 7.9 Hz); ^{13}C{^{1}H}-NMR (101 MHz, $CDCl_3$) δ 15.5 (CH_3), 21.2 (CH_3), 106.6 (CH_2), 126.7 (CH), 129.0 (CH), 136.9 (C), 138.3 (C), 146.3 (C). HRMS: m/z $[M+H]^+$ calc. 165.0733, found: 165.0730.

α-methylthio-3-methylstyrene ***2d***

Obtained as a slightly turbid beige oil (87.4 mg, 0.532 mmol, 96% purity (GC-MS) 21% yield over two steps). ^{1}H-NMR (400 MHz, $CDCl_3$) δ 2.31 (3 H, s), 3.36 (3 H, s), 4.95 (1 H, s), 5.42 (1 H, s), 7.11–7.16 (1 H, m), 7.19–7.27 (1 H, m), 7.30–7.36 (2 H, m); ^{13}C{^{1}H}-NMR (101 MHz, $CDCl_3$) δ 15.6 (CH_3), 21.4 (CH_3), 107.0 (CH_2), 124.0 (CH), 127.6 (CH), 128.2 (CH), 129.2 (CH), 138.0 (C), 139.7 (C), 146.5 (C). HRMS: m/z $[M+H]^+$ calc. 165.0733, found: 165.0731.

α-methylthio-2-methylstyrene ***2e***

For the second step, using 323 mg (0.949 mmol) of the intermediate, additional triethyl amine (600 µL, 4.31 mmol, 4.5 eq.) and acetonitrile (0.5 mL) as co-solvent were employed. The reaction was stirred for 20 h at 25 °C. α-methylthio-2-methylstyrene was obtained as a slightly turbid pale yellow oil (135 mg, 0.823 mmol, 80% purity (GC-MS) 32% yield over two steps). ^{1}H-NMR (400 MHz, $CDCl_3$) δ 2.26 (3 H, s), 2.37 (3 H, s), 5.08 (2 H, s), 7.12–7.33 (5 H, m); ^{13}C{^{1}H}-NMR (101 MHz, $CDCl_3$) δ 15.4 (CH_3), 19.5 (CH_3), 108.3 (CH_2), 125.5 (CH), 128.0 (CH), 129.2 (CH), 130.1 (CH), 135.9 (C), 139.8 (C), 146.1 (C). HRMS: m/z $[M+H]^+$ calc. 165.0733, found: 165.0731.

α-methylthio-4-fluorostyrene ***2f***

For the second step, using 476 mg (1.39 mmol) of the intermediate, additional triethyl amine (870 µL, 6.25 mmol, 4.5 eq.) and acetonitrile (0.5 mL) as co-solvent were employed. The reaction was stirred for. α-methylthio-4-fluorostyrene was obtained as a slightly turbid pale yellow oil (170 mg, 1.01 mmol, 94% purity (GC-MS) 40% yield over two steps). ^{1}H-NMR (400 MHz, $CDCl_3$) δ 2.31 (3 H, s), 4.95 (1 H, s), 5.39 (1 H, s), 6.99–7.06 (2 H, m), 7.47–7.53 (2 H, m); ^{13}C{^{1}H}-NMR (101 MHz, $CDCl_3$) δ 15.6 (CH_3), 107.3 (CH_2, d, *J* 1.1 Hz), 115.2 (CH, d, *J* 21.6 Hz), 128.6 (CH, d, *J* 8.1 Hz), 135.8 (C, d, *J* 3.5 Hz), 145.4 (C), 162.8 (C, d, *J* 247.6 Hz). HRMS: m/z $[M+H]^+$ calc. 181.0482, found: 181.0480.

*Synthesis of 2-(phenylthio)propene **2h***

2-(phenylthio)propene was synthesized following a literature protocol.[6] To an oven-dried Schlenk flask under nitrogen was added dry THF (30 mL), phenyl vinyl sulfide (**2g**, 654 mg, 4.80 mmol). The solution was degassed using three cycles of freeze-pump-thaw, and cooled to -78 °C using a dry ice/acetone bath. LDA (1 M in THF/ hexanes,10 mL, 10 mmol) was added over the course of 1 min, and the solution stirred for 20 min. Methyl iodide (1.30 mL, 20.9 mmol) was added slowly over 2 min, and the reaction stirred for 1 h. The cold bath was removed, and the reaction allowed to warm to ambient temperature over 20 min. The reaction was quenched with saturated ammonium chloride (15 mL) and extracted with pentane/diethyl ether (1:1, 2×10 mL). The organic extracts were combined and washed with hydrochloric acid (1M, 30 mL), water (2×30 mL), and brine (10 mL), and dried ($MgSO_4$). Solvent was removed *in vacuo*, giving a mixture of 2-(phenylthio)propene and phenyl vinyl sulfide (66:34; 591 mg). A portion (90 µL) of this crude product was purified by preparative HPLC:

Sample was prepared for injection by diluting 1:1 with acetonitrile. Shimadzu Prominence HPLC (DGU-20A5 degasser, LC-20AT pump module, SIL-20A HT auto-sampler CTO-20AC column oven, SPD-20A DAD detector, CBM-20A communication module, FRC-10A fraction collector), using a Restek Raptor ARC-18 column (150 mm × 4.6 mm × 2.7 µm). The wavelength was 210 nm. Oven temperature 30 °C, injection volume 20 µL, flow rate 1 mL/min. Method:

**Time (min)**	**milliQ**	**MeCN**
0	50	50
2	50	50
12	40	60
12.01	0	100
14	0	100
14.1	50	50
21	50	50

The product was collected, diluted with water (20 mL), and extracted with pentane (3×20 mL). The organic extracts were combined and washed with water (3×10 mL) and brine (10 mL), and dried ($MgSO_4$). Solvent was removed under a stream of nitrogen. After final drying under vacuum at ambient temperature, 2-(phenylthio)propene (38.7 mg) was obtained as a clear colourless oil. ^{1}H-NMR (400 MHz, $CDCl_3$) δ 2.31 (3 H, s), 4.95 (1 H, s), 5.39 (1 H, s), 6.99–7.06 (2 H, m), 7.47–7.53 (2 H, m); ^{13}C{^{1}H}-NMR (101 MHz, $CDCl_3$) δ 15.6 (CH_3), 107.3 (CH_2, d, *J* 1.1 Hz), 115.2 (CH, d, *J* 21.6 Hz), 128.6 (CH, d, *J* 8.1 Hz), 135.8 (C, d, *J* 3.5 Hz), 145.4 (C), 162.8 (C, d, *J* 247.6 Hz). HRMS: m/z $[M+H]^+$ calc. 151.0576, found: 151.0573.

Biotransformation reactions (0.5 mL scale)

Reactions were set up in a Coy chamber using degassed buffers and solvents that had been allowed to equilibrate in the COY chamber for at least 24 h. EREDS (<200 µL aliquots) were thawed in the COY ante chamber during over several vacuum cycles and allowed to equilibrate for approx. 15 min. Stock solutions were prepared in the COY chamber. To 1.5 mL glass vial with a screw top and PTFE septum was added (in the following order) Tris-HBr (50 mM, pH 7.5, 182-325 µL), D-Glucose (50 µL, 550 mM stock in Tris-HBr), $NADP^+$ (50 µL, 5 mM stock in Tris-HBr), JM GDH-101 (25 µL, 10 mg/mL stock in Tris-HBr), styrene (25 µL, 200 mM stock in DMSO), α-bromoacetophenone (25 µL, 200 mM stock in DMSO), and ERED (0-143 µL, 490-1300 µM), to a final reaction volume of 500 µL. Biotransformations were incubated in an Eppendorf

Thermomixer at 25 °C, 750 rpm for up to 24 h inside the COY chamber. Reactions were quenched aerobically by addition of acetonitrile (500 µL). HPLC samples were prepared by adding 200 µL of quenched reaction to 800 µL of acetonitrile, precipitated protein was removed by centrifugation (21,000 g, 2 min). GC-MS samples were prepared by extracting the remaining quenched reaction mixture with 600 µL EtOAc.

For the isolation of **3aa** from the reaction with NCR, 10 reactions as described above were carried out in parallel, and extracted each with EtOAc (2 × 1 mL). The extracts were combined, dried ($MgSO_4$), and EtOAc removed under a stream of nitrogen. After final drying under vacuum at ambient temperature, crude **3aa** was obtained (56.9 mg) as a pale yellow oil, which was purified by preparative TLC (Macherey-Nagel Sil G-50 UV (20 cm × 20 cm × 0.5 mm); pentane:diethyl ether 98:2; 2 passes), obtaining **3aa** (4.0 mg, 30% yield, 76% *ee*) as a white solid. ^{1}H-NMR (400 MHz, $CDCl_3$) δ 1.9 (3 H, s), 2.23–2.42 (2 H, m), 2.96–3.11 (2 H, m), 3.80 (1 H, dd, *J* 8.0, 7.3 Hz), 7.22–7.28 (1 H, m), 7.33 (4 H, app. d, *J* 4.4 Hz), 7.40–7.46 (2 H, m), 7.54 (1 H, tt, *J* 7.4, 1.3 Hz), 7.87–7.91 (2 H, m); ^{13}C{^{1}H}-NMR (101 MHz, $CDCl_3$) δ 14.2 (CH_3), 30.1 (CH_2), 36.4 (CH_2), 50.7 (CH), 127.2 (CH), 127.8 (CH), 128.0 (CH), 128.5 (CH), 128.6 (CH), 133.0 (CH), 136.8 (C), 141.9 (C), 199.4 (C). HRMS: m/z $[M+H]^+$ calc. 271.1151, found: 271.1146.

For the isolation of **3ha** from the reaction with GluER T36A, 8 reactions as described above were carried out in parallel, and extracted each with EtOAc (1 mL). The extracts were combined, dried ($MgSO_4$), and EtOAc removed under a stream of nitrogen. The crude residue was purified by preparative TLC (Macherey-Nagel Sil G-50 UV (20 cm × 20 cm × 0.5 mm); pentane:diethyl ether 98:2; 2 passes), obtaining **3aa** (2.0 mg, 18% yield, 72% *ee*) as a white solid. ^{1}H-NMR (400 MHz, $CDCl_3$) δ 1.35 (3 H, d *J* 6.8 Hz), 1.93–2.11 (2 H, m), 3.13–3.26 (2 H, m), 3.35 (1 H, dqd, *J* 7.8, 6.8, 5.6 Hz), 7.19–7.24 (1 H, m), 7.25–7.30 (2 H, m), 7.38–7.41 (2 H, m), 7.43–7.48 (2 H, m), 7.56 (1 H, tt, *J* 7.4, 1.4 Hz), 7.93–7.97 (2 H, m); ^{13}C{^{1}H}-NMR (101 MHz, $CDCl_3$) δ 21.5 (CH_3), 30.8 (CH_2), 35.8 (CH_2), 43.1 (CH), 126.9 (CH), 128.0 (CH), 128.6 (CH), 128.9 (CH), 132.1 (CH), 133.0 (CH), 134.8 (C), 136.9 (C), 199.7 (C). HRMS: m/z $[M+H]^+$ calc. 271.1151, found: 271.1139.

*Preparative scale biotransformation—synthesis of **3aa***

A round bottom flask was charged with a magnetic stirrer, JM GDH-101 (10 mg), $NADP^+$ disodium (7.8 mg, 9.91 µmol), D-glucose monohydrate (396.4, 2 mmol), and α-bromoacetophenone (79.6 mg, 400 µmol). The flask was then brought into a COY chamber, and Tris-HBr (15 mL, 100 mM, pH 7.5), DMSO (1 mL), and α-(methylthio)styrene (1 mL of a 400 mM stock solution in DMSO) were added. Finally, GluER T36A (3.02 mL, 1300 µM stock, 3.92 µmol) was added, the flask sealed with a rubber septum and wrapped in aluminium foil, and the reaction was stirred (at a speed just below vortex formation) in the COY chamber for 26 h at ambient temperature (23-24 °C). Brine (10 mL) was added, and the reaction was extracted with EtOAc (3×20 mL), breaking the emulsion with gentle heating when needed. The combined organic extracts were washed with brine (10 mL), dried ($MgSO_4$), and solvent was removed *in vacuo* giving crude **3aa** (162.9 mg) as a pale yellow very viscous oil, which was purified by preparative TLC (Supelco PLC Silica Gel 60 F_{254} (20 cm × 20 cm × 2 mm; 20 cm × 4 cm concentrating zone); pentane:diethyl ether 98:2; 4 passes), obtaining **3aa** (49.5 mg, 46% yield, 93% *ee*) as a clear oil which solidified over time. ^{1}H-NMR (400 MHz, $CDCl_3$) δ 1.9 (3 H, s), 2.23–2.42 (2 H, m), 2.96–3.11 (2 H, m), 3.80 (1 H, dd, *J* 8.0, 7.2 Hz), 7.22–7.28 (1 H, m), 7.33 (4 H, app. d, *J* 4.4 Hz), 7.40–7.46 (2 H, m), 7.54 (1 H, tt, *J* 7.4, 1.4 Hz), 7.87–7.91 (2 H, m); ^{13}C{^{1}H}-NMR

(101 MHz, $CDCl_3$) δ 14.2 (CH_3), 30.1 (CH_2), 36.4 (CH_2), 50.7 (CH), 127.2 (CH), 127.8 (CH), 128.0 (CH), 128.5 (CH), 128.6 (CH), 133.0 (CH), 136.8 (C), 141.9 (C), 199.4 (C).

Stopped-flow Kinetics

Experiments were set up in a glove box (Belle Technology, O_2 <3 ppm) using degassed buffers and solvents that had been allowed to equilibrate in the glove box for at least 24 h. The instrument used was an Applied Photophysics stopped-flow spectrophotometer with a photodiode array detector. The temperature was set to 25 °C. GluER T36A was allowed to thaw in the antechamber and allowed to equilibrate in the glove box for 1h. It was then diluted to 40 µM using Tris-HBr (50 mM, pH 7.5) and DMSO (20% *v/v*), and reduced by titrating NADPH. Substrate solutions for each concentration were prepared by diluting 5x concentrated DMSO stocks with Tris-HBr (50 mM, pH 7.5). Final concentrations after mixing were: **1a** (0-3 mM), **2a** (0-750 µM), GluER T36A (20 µM), Tris-HBr (40 mM), DMSO (20% *v/v*), pH 7.5. Whole spectra were acquired from 300-730nm, acquiring 500 spectra per reaction. Reactions were measured in triplicate at each substrate concentration. Spectra were corrected for baseline drift at 700 to 730 nm. The oxidation of FMN was followed by monitoring at 474 nm, and a single exponential ($y_0 + Ae^{-k_{\mathrm{obs}}t}$) was fitted to each transient. An appropriate kinetic equation was then fitted to the rate constants k_{obs}.

X-ray Crystallography

3aa (11.2 mg) was dissolved in ethanol (1.5 mL) and water (800 µL) was added slowly. After filtering (0.45 µm PTFE), the solution was allowed to slowly evaporate upon which thin needles formed. A single crystal of compound **3aa** was mounted on top of a cryoloop and transferred into the cold nitrogen stream (100 K) of a Bruker-AXS D8 Venture diffractometer. Data collection and reduction was done using the Bruker software suite APEX4.[7] The final unit cell was obtained from the xyz centroids of 9940 reflections after integration. A multiscan absorption correction was applied, based on the intensities of symmetry-related reflections measured at different angular settings (SADABS). The structures were solved by direct methods using SHELXT and refinement of the structure was performed using SHELXL.[8,9] The hydrogen atoms were generated by geometrical considerations, constrained to idealised geometries and allowed to ride on their carrier atoms with an isotropic displacement parameter related to the equivalent displacement parameter of their carrier atoms. The absolute structures was chosen based on refinement of Flack's x-parameter (0.00(2)) from anomalous scattering.[10] Crystal data and details on data collection and refinement are presented in **Table S1**. CCDC deposition number (2410188).

Table S1. Crystallographic data for **3aa**.

chem formula	C17 H18 O S
M_r	270.37
cryst syst	Triclinic
color, habit	colorless, needle
size (mm)	0.31 x 0.03 x 0.02
space group	P1
a (Å)	5.5592(2)
b (Å)	7.3157(3)
c (Å)	9.4698(3)
α, deg	88.665(1)
β, deg	84.905(1)
γ, deg	69.126(1)
V ($Å^3$)	358.42(2)
Z	1
ρ_{calc}, g.cm^{-3}	1.253
μ(Cu K$\bar{\alpha}$), mm^{-1}	1.901
F(000)	144
temp (K)	100(2)
θ range (deg)	4.688 – 74.514
data collected (h,k,l)	-6:6, -8:9, -11:11
no. of rflns collected	12086
no. of indpndt reflns	2783
observed reflns	2763 ($F_o \geq 2\ \sigma(F_o)$)
R(F) (%)	5.44
wR(F^2) (%)	13.78
GooF	1.051
Weighting a,b	0.1157, 0.0510
params refined	173
restraints	3
min, max resid dens	-0.431, 0.435

Protein Mass-spectrometry

Reactions were set up as described above, containing either no substrate, **2a**, **1a**, or both **1a** and **2a**. After 24 h, all reactions were colourless except for the reaction containing only **1a**. SDS-PAGE samples were prepared by combining the reaction mixture (10 µL), milliQ water (20 µL), and 4x Lämmli buffer (10 µL). Following gel-electrophoresis, gels were stained using Coomassie (GelCode^{TM} Blue Safe Protein Stain), and bands corresponding to GluER T36A were excised and sliced thinly. Samples were destained in 1.5 mL Eppendorf LoBind tubes using 1 mL of ammonium bicarbonate (100 mM) in LC-MS grade acetonitrile (40% *v*/*v*) and water, for 15 min at ambient temperature with gentle shaking. The supernatant was discarded. LC-MS grade ammonium bicarbonate buffer (100 mM, 98 µL) was added followed by 2 µL of modified trypsin, sequencing grade (Promega cat# 5111), that had been resuspended in hydrochloric acid (1 mM) to 0.1 µg/µL. Following gentle mixing, samples were incubated at 37 °C, 300 rpm for 22 h. The supernatant was removed, and the gel pieces were incubated with 150 µL of LC-MS grade formic acid (5% *v*/*v*) in LC-MS grade acetonitrile (70% *v*/*v*) and water for 15 min at 37 °C, 300 rpm. The supernatant was removed, and the gel pieces were incubated with 100 µL of LC-MS grade acetonitrile for 15 min at 37 °C, 300 rpm. The supernatant was removed, and the gel pieces were incubated with 100 µL of LC-MS grade acetonitrile (10% *v*/*v*) and water for 15 min at 37 °C, 300 rpm. The supernatant was removed. Supernatants were combined and dried in a centrifuge concentrator at 45 °C. Samples were stored at -20 °C, and resuspended with 15 µL of TFA (0.01% *v*/*v*) in LC-MS grade acetonitrile (3% *v*/*v*) and water for analysis. Approx. 2 µL of each sample was analyzed using an EASY nano-LC 1200, equipped with an Acclaim PepMap RSLC RP C18 separation column (50 µm × 150 mm, 2 µm), and a QE plus Orbitrap mass spectrometer (Thermo Fisher Scientific, Germany). Solvent A was H_2O containing 0.1% formic acid, and solvent B consisted of 80% ACN in H_2O and 0.1% formic acid. The mass spectrometer was operated in data-dependent acquisition mode, where the top 10 most intense precursor ions were selected for fragmentation using higher-energy collisional dissociation (HCD) using a normalized collision energy (NCE) of 28. Mass spectrometric raw data were database searched using a protein reference database from *E. coli* BL21 including the sequence of the recombinantly expressed protein and sequences of common lab contaminants, using PEAKS Studio X (Bioinformatics Solutions Inc., Waterloo, Canada). Database searching allowed 20 ppm parent ion and 0.02 m/z fragment ion mass error, 3 missed cleavages, methionine oxidation and N/Q deamidation as variable modifications. Peptide spectrum matches were filtered for 0.1% false discovery rates (FDR) and identifications with ≥2 unique peptides were considered as significant. A second-round search was performed using PEAKS PTM (SPIDER) including all built-in modifications, performing PTM search on spectra with a de novo ALC score >15, allowing max 2 variable modifications per peptide. Peptides corresponding to GluER T36A were sorted by starting amino acid. Difference maps (**Figure 5D, Figure S6**) were generated by subtracting the area of each peptide found in the reaction without any substrate from the areas found in samples with either or both substrates. Histograms (**Figure S7**) were generated by summation of the areas of all modified and unmodified peptides with the same starting amino acid. Normalization (by dividing by total area) had no effect on samples without and with either **2a** or both substrates, while for the samples with only **1a**, the same peptides were identified as being decreased. As no corresponding modified peptides were identified, this method of normalization inflated the abundance of non-decreased peptides and thus data are being presented without normalization.

UV-vis spectroscopy of reactions without vinyl co-substrate

Reactions were set up as described above, containing either **1a**, no substrate, **1a** without GluER T36A, **1a** without GDH, no substrate and no GDH. After 24 h, reactions were diluted four-fold with Tris-HBr (50 mM, pH 7.5) and DMSO (20% *v*/*v*). Precipitate was removed by centrifugation, and the spectrum was measured from 240 nm to 760 nm on an Avantes AvaLight-DH-S-BAL spectrophotometer.

Docking studies

Structures for GluER T36A (6MYW) and PETNR (1GVO) were retrieved from the protein databank. Additional subunits, substrate, ions, and waters were removed in incentive PyMOL 3.0.5. The structures were then loaded into YASARA 20.12.24 and the forcefield AMBER03 was applied. The enzymes were protonated (pH 7.4), and a simulation cell created. The benzylic radical intermediate was generated in IQmol, and the planar benzylic atom was fixed by manually applying bond orders in YASARA. Flexible docking was carried out using a customized version of dock_run.mcr, using AutodockLGA (100 poses without clustering). The following residues were set as flexible: GluER T36A: H172, N175, Y177, Y343, T25, W66, W100, F269; and PETNR: H181, H184, Y186, Y351, T26, Y68, W102, L275. Docking poses were chosen based on the distance between the benzylic carbon and the N5 of FMN.

Analytical methods

Reverse phase HPLC

Conversions were determined by RP-HPLC-DAD on a Shimadzu Nextera HPLC (DGU-405 degasser, LC-40D pump module, SIL-40 auto-sampler CTO-40C column oven, SPD-M40 DAD detector) using a Restek Raptor ARC-18 column (150 mm × 4.6 mm × 2.7 µm). Oven temperature 30 °C, injection volume 2 µL, flow rate 1 mL/min. Gradient:

Time (min)	milliQ (TFA 0.1%)	MeCN (TFA 0.1%)
0	5	95
2	5	95
12	0	100
14	0	100
14.1	5	95
21	5	95

Chiral reverse phase HPLC

Enantioselectivities were determined by RP-HPLC-UV on a Shimadzu Prominence HPLC (DGU-20A5 degasser, LC-20AT pump module, SIL-20A HT auto-sampler, CTO-20AC column oven, SPD-20A UV-VIS detector, CBM-20A communication module), using a Phenomenex Lux Cellulose-4 column (250 mm × 4.6 mm × 5 µm). The wavelength was 210 nm. Oven temperature 30 °C, injection volume 2-10 µL, flow rate 1 mL/min.

The following isocratic methods were used:

Solvent A: milliQ (TFA 0.1%), Solvent B: MeCN (TFA 0.1%).

For **3aa**, **3ba**, **3ca**, **3da**, **3f**, **3ac**, **3ad**: 40% A, 60% B.

For **3ab**: 50% A, 50% B.

Chiral normal phase HPLC

Enantioselectivities were determined by RP-HPLC-UV on a Shimadzu Prominence HPLC (LC-20D pump module, SIL-20A HT auto-sampler, CTO-20A column oven, SPD-40 UV-VIS detector, CBM-20A communication module), using a Phenomenex Lux i-Cellulose-5 column (250 mm × 4.6 mm × 3 µm). The wavelength was 240 nm. Oven temperature 30 °C, injection volume 2 µL, flow rate 1 mL/min.

The following isocratic method was used:

For **3ha**: 99.5% heptane, 0.5% isopropanol.

GC-MS

Styrene substrates and biotransformation were analyzed by GC-MS to verify product identities/purities on a Shimadzu GC-2010 Plus equipped with a GCMS-QP2010 SE mass spectrometer and AOC-20i Plus autosampler, using a Macherey-Nagel Optima 1 MS (30 m × 0.25 mm × 0.25 µm) column. 1 µL of sample was injected with a split ratio of 10:1 and an injector temperature of 250 °C. Helium was used as the carrier gas, with a linear velocity of 37 cm/s. The ion source temperature was 200 °C, the interface temperature was 250 °C.

Temperature program:

Ramp (°C/min)	Temperature (°C)	Hold (min)
	50	1
10	345	1

References

(1) Page, C. G.; Cao, J.; Oblinsky, D. G.; MacMillan, S. N.; Dahagam, S.; Lloyd, R. M.; Charnock, S. J.; Scholes, G. D.; Hyster, T. K. Regioselective Radical Alkylation of Arenes Using Evolved Photoenzymes. *J. Am. Chem. Soc.* **2023**, *145*, 11866–11874. https://doi.org/10.1021/jacs.3c03607.

(2) Fu, H.; Lam, H.; Emmanuel, M. A.; Kim, J. H.; Sandoval, B. A.; Hyster, T. K. Ground-State Electron Transfer as an Initiation Mechanism for Biocatalytic C-C Bond Forming Reactions. *J. Am. Chem. Soc.* **2021**, *143*, 9622–9629. https://doi.org/10.1021/jacs.1c04334.

(3) Aliverti, A.; Curti, B.; Vanoni, M. A. Identifying and Quantitating FAD and FMN in Simple and in Iron-Sulfur-Containing Flavoproteins. In *Flavoprotein Protocols*; Chapman, S. K. ., Reid, G. ., Eds.; Humana press: Totowa, New Jersey, 1999; Vol. 131, pp 9–23. https://doi.org/https://doi.org/10.1385/1-59259-266-X:9.

(4) Chow, Y. L.; Bakker, B. H.; Iwai, K. Dimethyl-α-Styrylsulphonium Bromide as a Reaction Intermediate. *J. Chem. Soc. Chem. Commun.* **1980**, No. 11, 521–522. https://doi.org/10.1039/C39800000521.

(5) Qiao, N.; Xin, X. Y.; Wang, W. M.; Wu, Z. L.; Cui, J. Z. Two Novel Ln8 Clusters Bridged by CO32− Effectively Convert CO2 into Oxazolidinones and Cyclic Carbonates. *Dalt. Trans.* **2023**, *1*, 10725–10736. https://doi.org/10.1039/d3dt01465g.

(6) Reich, H. J.; Willis, W. W.; Clark, P. D. Vinyl Selenides and Selenoxides: Preparation, Conversion to Lithium Reagents, Diels-Alder Reactivity, and Some Comparisons with Sulfur Analogues. *J. Org. Chem.* **1981**, *46*, 2775–2784. https://doi.org/10.1021/jo00326a035.

(7) Bruker (2021). APEX4 (Version 2021.4-0), SAINT (Version 8.40A) and SADABS (Version 2016/2). Bruker AXS Inc., Madison, Wisconsin, USA. Bruker AXS Inc., Madison, Wisconsin, USA.

(8) Sheldrick, G. M. Crystal Structure Refinement with SHELXL. *Acta Crystallogr. Sect. C Struct. Chem.* **2015**, *71*, 3–8. https://doi.org/10.1107/S2053229614024218.

(9) Sheldrick, G. M. SHELXT - Integrated Space-Group and Crystal-Structure Determination. *Acta Crystallogr. Sect. A Found. Crystallogr.* **2015**, *71*, 3–8. https://doi.org/10.1107/S2053273314026370.

(10) Flack, H. D. On Enantiomorph-polarity Estimation. *Acta Crystallogr. Sect. A* **1983**, *39*, 876–881. https://doi.org/10.1107/S0108767383001762.

Supplementary Figures

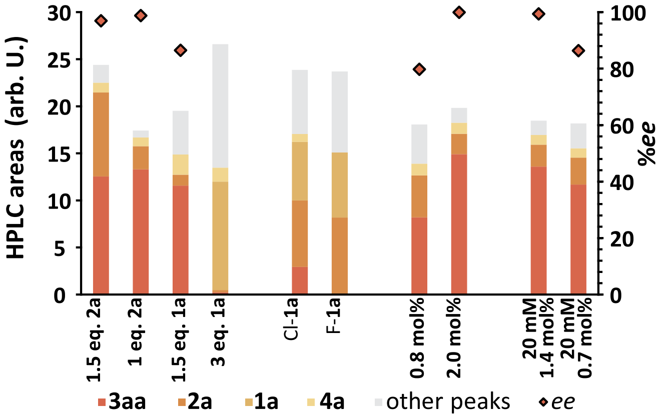

Figure S2: HPLC areas (210 nm) for reactions using different deviations from standard conditions. Standard conditions: D-glucose (55 mM), $NADP^+$ (0.5 mM), JM GDH-101 (0.5 mg mL^{-1}), **1a** (10 mM), **2a** (10 mM), ERED (1.4 mol%), Tris-HBr (50 mM), pH 7.5, 25 °C, 750 rpm, anoxic, 24 h (20 mM scale reactions D-glucose (100 mM), Tris-HBr (100 mM)).

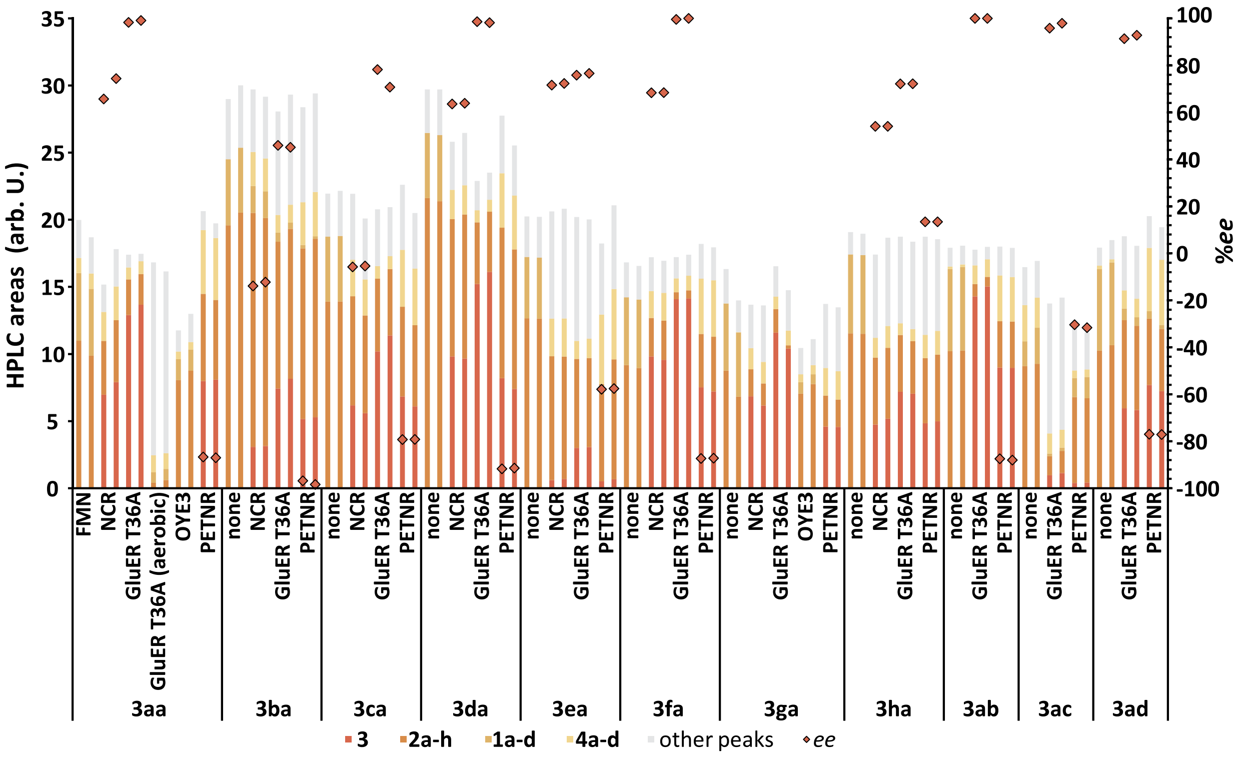

Figure S3: HPLC areas (210 nm) for reactions using different substrates and enzyme combinations. Two replicates are shown for each combination. Conditions: D-glucose (55 mM), $NADP^+$ (0.5 mM), JM GDH-101 (0.5 mg mL^{-1}), **1a-d** (10 mM), **2a-h** (10 mM), ERED (1.4 mol%), Tris-HBr (50 mM), pH 7.5, 25 °C, 750 rpm, anoxic, 24 h.

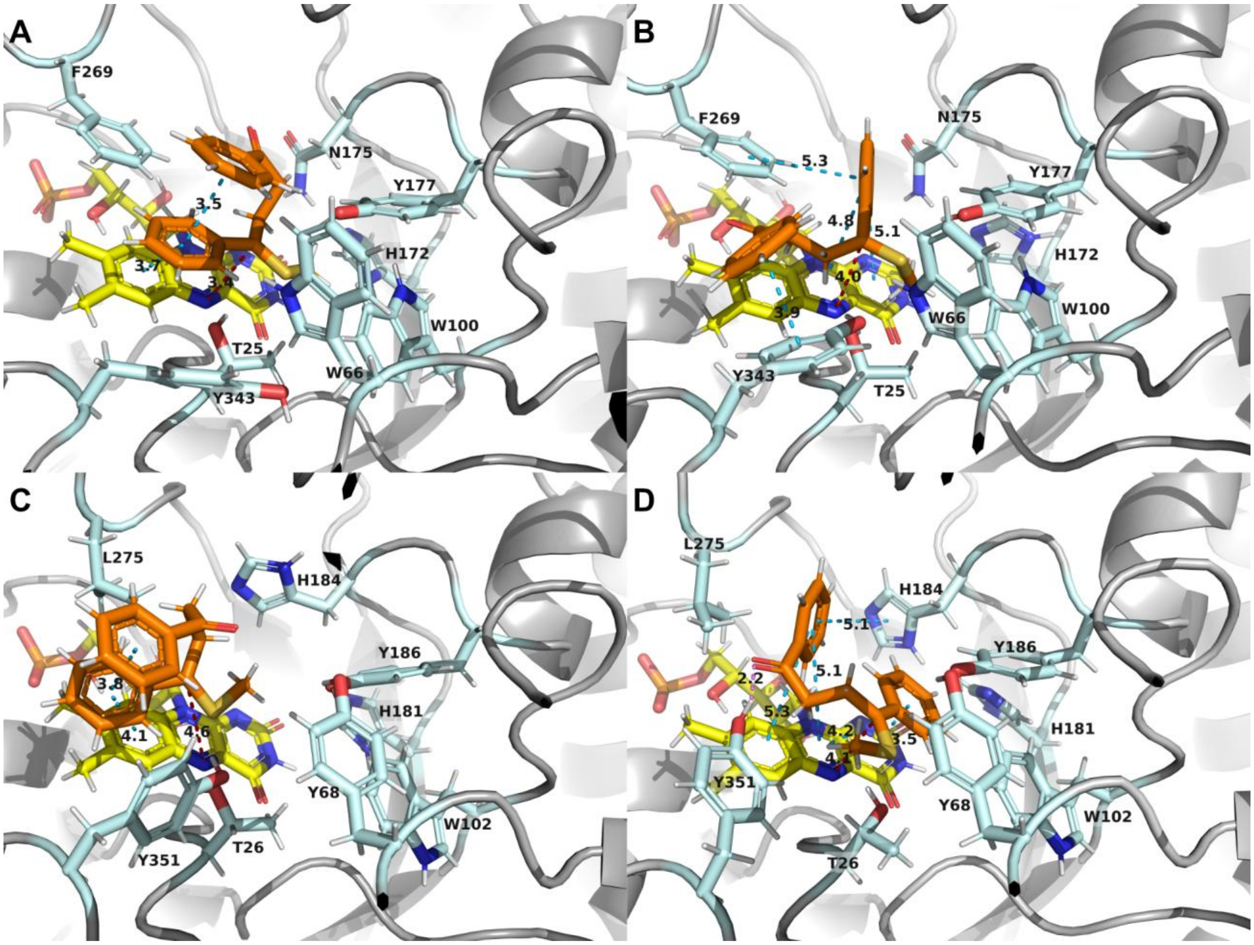

Figure S4: Docking poses of the prochiral benzylic radical intermediate 3aa• into the active sites of GluER T36A (PDB: 6MYW) and PETNR (PDB: 1GVO). **A**: pro-(*R*) binding pose in GluER T36A. **B**: pro-(*S*) binding pose in GluER T36A. **C**: pro-(*R*) binding pose in PETNR. **D**: pro-(*S*) binding pose in PETNR. Distances are shown as follows: blue: π-π interactions, pink: hydrogen bond, red: catalytic distance of benzylic carbon and flavin N5. Residues shown in light blue were flexible during docking.

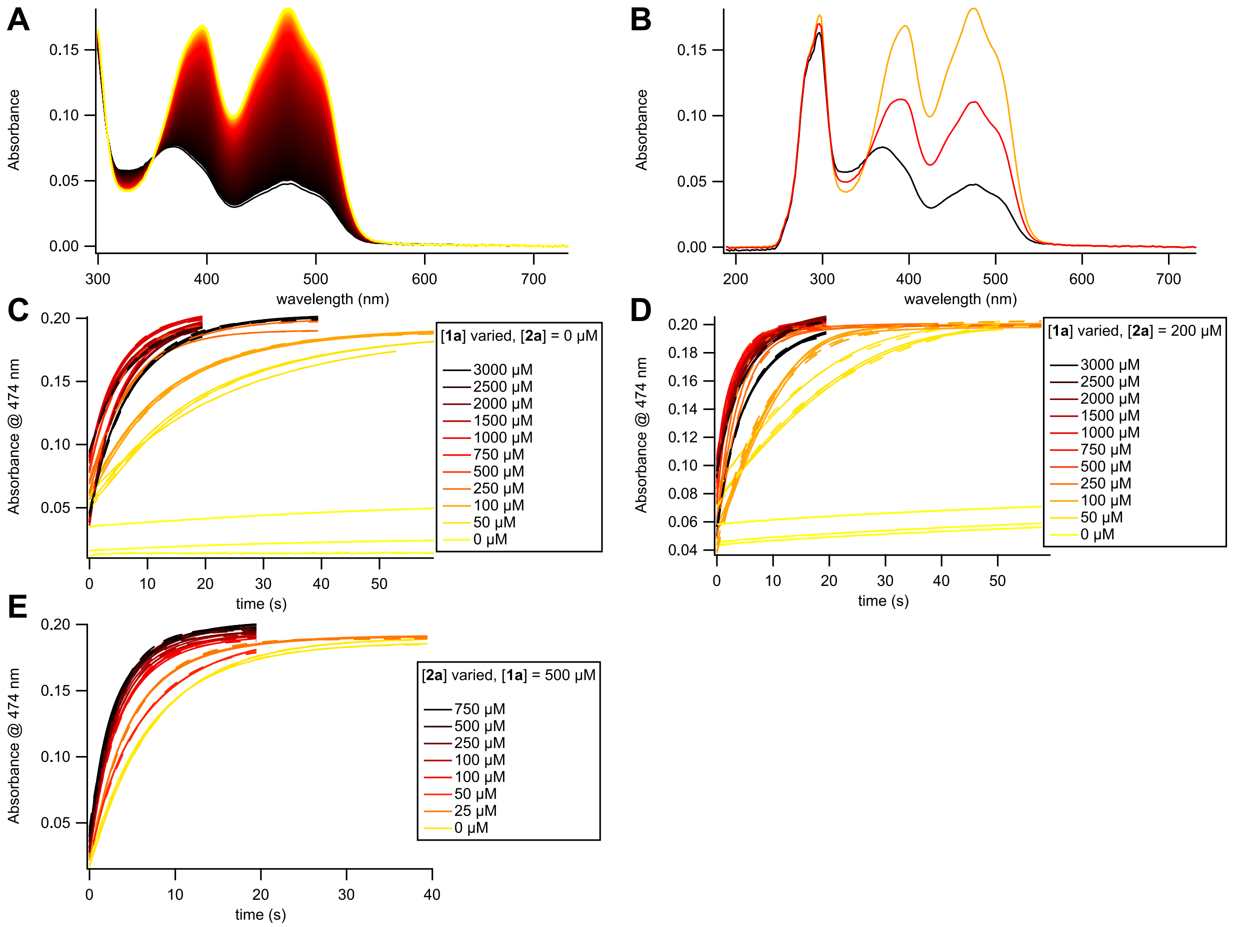

Figure S5: Stopped-flow experiments. **A**: representative time-course (black to red to yellow); 50 µM **1a** 0 µM **2a**. **B**: Same data, showing only spectra for t=0 s (black), t=12 s, and t=60 s. The semiquinone is not observed. **C**: exponential fits to the transients at 474 nm for data with [**2a**]=0. **D**: exponential fits to the transients at 474 nm for data with [**2a**]=200 µM. **E**: exponential fits to the transients at 474 nm for data with [**1a**]=500 µM.

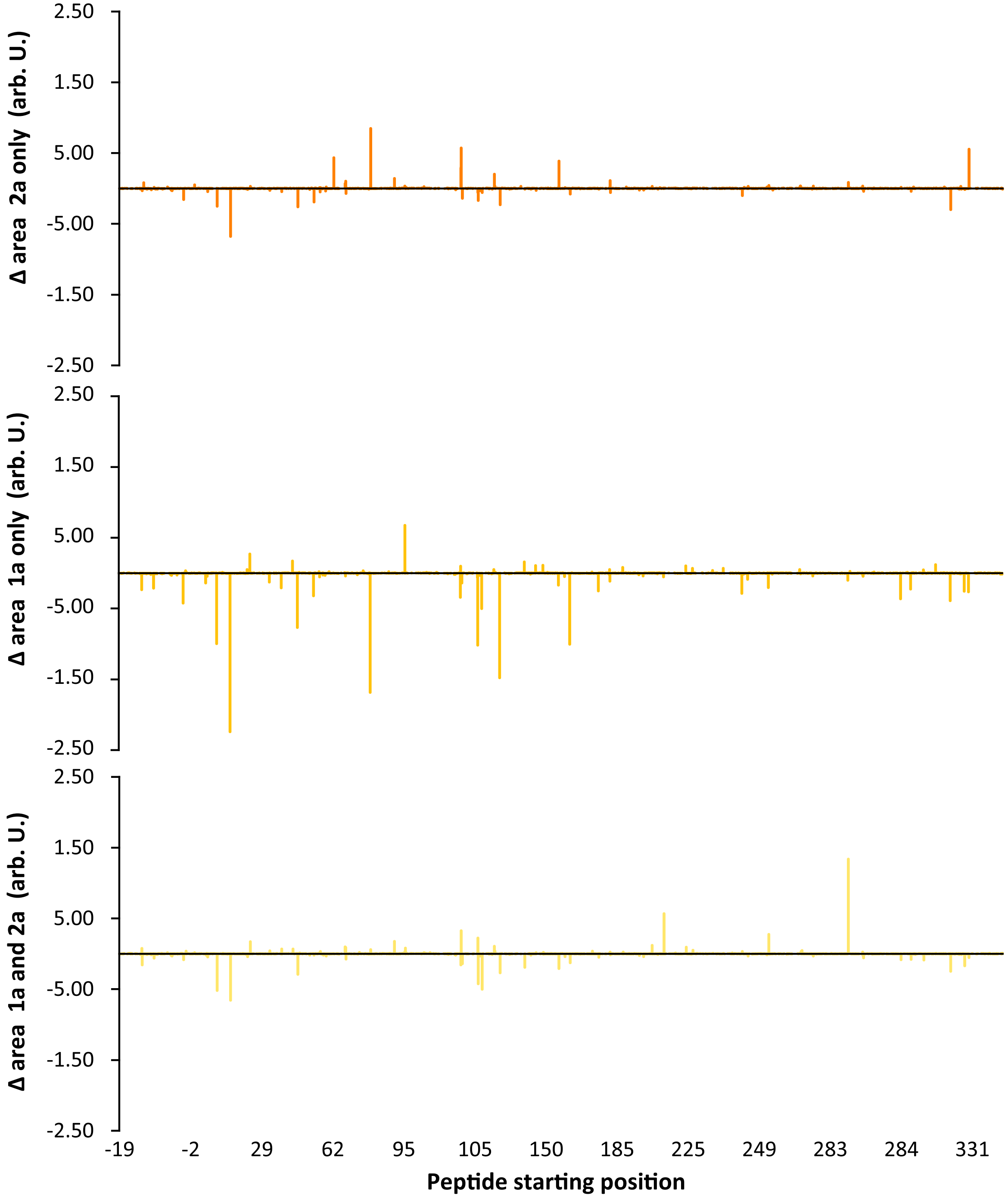

Figure S6: Difference in peptide abundance after tryptic digest of the band of GluER T36A in the reactions with **2a** only, **1a** only, and both **1a** and **2a**, compared to the reaction without substrate. In these graphs, all observed peptides are shown individually. Some peptides contain modifications, for example oxidation or deamidation, which may have formed at any stage from enzyme production to mass spectrometry sample preparation.

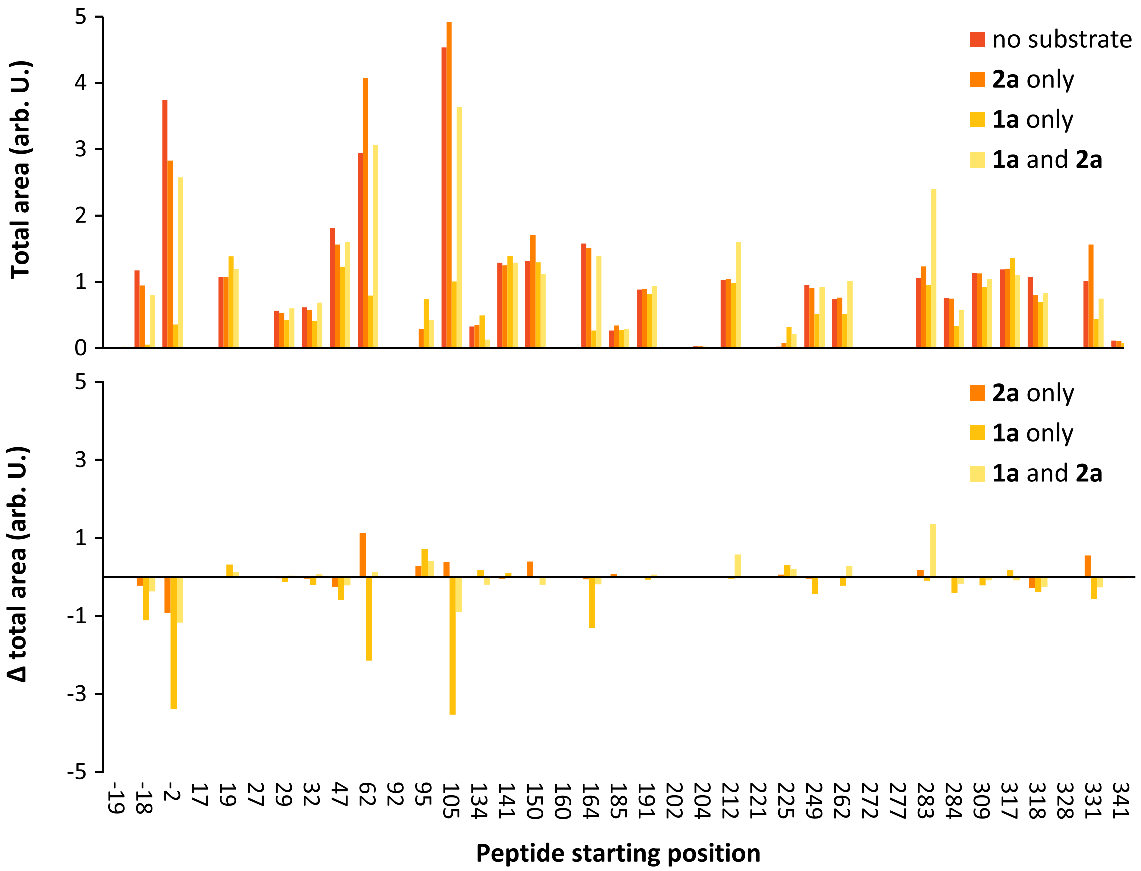

Figure S7: **Top:** Abundance of peptides observed, grouped by starting position, after tryptic digest of the band of GluER T36A in the reactions with **2a** only, **1a** only, and both **1a** and **2a**, compared to the reaction without substrate. **Bottom:** Difference in peptide abundance, grouped by starting position, after tryptic digest of the band of GluER T36A in the reactions with **2a** only, **1a** only, and both **1a** and **2a**, compared to the reaction without substrate. The total area includes both modified and unmodified peptides. Some peptides contain modifications, for example oxidation or deamidation, which may have formed at any stage from enzyme production to mass spectrometry sample preparation.

DNA and protein sequences

pET22b-NCR

MPSLFDPIRFGAFTAKNRIWMAPLTRGRATRDHVPTEIMAEYYAQRASAGLIISEATGISQEGLGWPYAPGIWSDAQVEAWLPITQAVHDAGGLIF
AQLWHMGRMVPSNVSGMQPVAPSASQAPGLGHTYDGKKPYDVARALRLDEIPRLLDDYEKAARHALKAGFDGVQIHAANGYLIDEFIRDSTNHR
HDEYGGAVENRIRLLKDVTERVIATIGKERTAVRLSPNGEIQGTVDSHPEQVFIPAAKMLSDLDIAFLGMREGAVDGTFGKTDQPKLSPEIRKVFKPPL
VLNQDYTFETAQAALDSGVADAISFGRPFIGNPDLPRRFFEKAPLTKDVIETWYTQTPKGYTDYPLLGDLEHHHHHH

ATGCCGTCACTGTTCGATCCAATCCGCTTTGGGGCTTTCACTGCAAAAAATCGTATCTGGATGGCGCCGTTAACACGGGGTCGGGCAACCCGT
GACCATGTCCCAACAGAGATAATGGCTGAATACTATGCCCAACGCGCATCCGCGGGCTTGATCATCAGCGAGGCGACCGGGATCAGCCAAGA
GGGCCTGGGCTGGCCCTATGCACCAGGAATCTGGAGTGATGCGCAGGTCGAGGCATGGTTACCCATAACCCAAGCGGTACACGATGCCGGA
GGTTTGATATTTGCACAACTGTGGCACATGGGGCGTATGGTGCCTTCCAACGTTTCTGGAATGCAACCTGTCGCACCTAGCGCTTCACAAGCG
CCCGGCTTGGGCCATACTTATGATGGCAAAAAGCCATACGATGTAGCCAGAGCATTGAGACTTGACGAGATCCCACGGCTGCTGGACGACTA
TGAAAAGGCAGCTCGGCACGCACTGAAAGCTGGGTTCGATGGAGTTCAGATTCATGCTGCCAACGGATACCTGATTGACGAGTTCATCCGGG
ATTCAACAAATCATAGACACGACGAATACGGGGGGGCGGTTGAGAACAGAATACGGTTATTGAAGGATGTCACTGAGCGGGTTATCGCAACC
ATCGGAAAGGAGCGCACAGCAGTGCGTTTAAGTCCGAATGGAGAGATACAAGGCACAGTAGACTCGCATCCAGAACAGGTATTTATCCCGGC
TGCAAAGATGTTATCTGATTTAGATATCGCGTTCCTTGGGATGCGCGAGGGTGCTGTAGACGGGACATTTGGCAAAACAGACCAGCCCAAACT
TTCGCCCGAGATCCGTAAAGTTTTCAAGCCACCCCTTGTTCTGAATCAAGATTACACTTTCGAGACTGCCCAGGCTGCGTTAGATTCGGGTGTA
GCCGATGCAATCAGTTTTGGTCGTCCATTCATTGGGAATCCCGACTTACCGAGAAGATTCTTTGAAAAGGCACCGTTAACTAAGGACGTAATT
GAGACTTGGTACACTCAGACTCCCAAAGGTTACACCGACTATCCACTGTTAGGTGATCTCGAGCACCACCACCACCACCACTGA

pET28a-GluER T36A

MGSSHHHHHHSSGLVPRGSHMPTLFDPIDFGPIHAKNRIVMSPLTRGRADKEAVPTPIMAEYYAQRASAGLIITEATGISREGLGWPFAPGIWSDA
QVEAWKPIVAGVHAKGGKIVCQLWHMGRMVHSSVTGTQPVSSSATTAPGEVHTYEGKKPFEQARAIDAADISRILNDYENAARNAIRAGFDGVQI
HAANGYLIDEFLRNGTNHRTDEYGGVPENRIRFLKEVTERVIAAIGADRTGVRLSPNGDTQGCIDSAPETVFVPAAKLLQDLGVAWLELREPGPNGT
FGKTDQPKLSPQIRKVFLRPLVLNQDYTFEAAQTALAEGKADAIAFGRKFISNPDLPERFARGIALQPDDMKTWYSQGPEGYTDYPSATSGPN

ATGGGCAGCAGCCATCATCATCATCATCACAGCAGCGGCCTGGTGCCGCGCGGCAGCCATATGCCTACCCTGTTCGACCCGATCGACTTCGGT
CCGATCCACGCTAAAAACCGTATCGTTATGTCTCCGCTGACCCGTGGCCGTGCGGATAAAGAAGCGGTGCCGACCCCGATCATGGCTGAATAC
TACGCGCAGCGCGCGTCCGCGGGCCTGATCATCACTGAAGCAACCGGTATCTCTCGTGAAGGCCTGGGTTGGCCGTTCGCGCCGGGTATCTG
GTCTGACGCGCAGGTTGAAGCCTGGAAACCGATCGTTGCTGGCGTTCACGCTAAAGGCGGTAAAATCGTTTGCCAGCTGTGGCACATGGGCC
GTATGGTACACTCTTCTGTGACCGGCACCCAGCCAGTTTCCTCCTCTGCTACCACTGCGCCGGGTGAAGTACACACTTATGAAGGCAAAAAACC
GTTCGAACAGGCTCGTGCGATCGACGCGGCAGACATTTCTCGTATCCTGAACGATTATGAAAACGCTGCGCGTAACGCAATCCGCGCTGGTTT
CGATGGCGTTCAGATCCACGCAGCGAACGGTTACCTGATTGACGAGTTCCTGCGTAACGGCACCAACCACCGCACCGATGAATACGGTGGCG
TACCGGAAAACCGTATCCGTTTCCTGAAAGAAGTGACTGAACGTGTGATCGCAGCTATCGGTGCGGATCGTACCGGTGTTCGTCTGTCTCCGA
ACGGTGACACCCAGGGTTGCATTGACTCTGCGCCGGAAACCGTGTTCGTTCCGGCGGCTAAACTGCTGCAGGATCTGGGTGTTGCGTGGCTG
GAACTGCGTGAACCGGGTCCGAACGGTACTTTCGGTAAAACCGATCAGCCGAAACTGTCTCCGCAGATCCGTAAAGTTTTCCTGCGTCCGCTG
GTTCTGAACCAGGACTACACCTTCGAAGCAGCGCAGACCGCTCTGGCGGAAGGTAAAGCTGACGCGATCGCTTTCGGTCGTAAATTCATCTCT
AACCCGGACCTGCCGGAACGTTTCGCGCGTGGTATCGCGCTGCAGCCGGACGACATGAAAACCTGGTACTCCCAGGGTCCGGAAGGTTACAC
CGACTACCCGTCCGCGACCAGCGGCCCGAACTAA

pET21a-PETNR

MSAEKLFTPLKVGAVTAPNRVFMAPLTRLRSIEPGDIPTPLMGEYYRQRASAGLIISEATQISAQAKGYAGAPGLHSPEQIAAWKKITAGVHAEDGRI
AVQLWHTGRISHSSIQPGGQAPVSASALNANTRTSLRDENGNAIRVDTTTPRALELDEIPGIVNDFRQAVANAREAGFDLVELHSAHGYLLHQFLSP
SSNQRTDQYGGSVENRARLVLEVVDAVCNEWSADRIGIRVSPIGTFQNVDNGPNEEADALYLIEELAKRGIAYLHMSETDLAGGKPYSEAFRQKVRE
RFHGVIIGAGAYTAEKAEDLIGKGLIDAVAFGRDYIANPDLVARLQKKAELNPQRPESFYGGGAEGYTDYPSLHHHHHH

ATGTCCGCTGAAAAGCTGTTTACCCCACTGAAAGTGGGTGCCGTTACTGCCCCAAACCGCGTGTTTATGGCCCCACTTACCCGTCTGCGCAGCA
TCGAGCCGGGCGATATCCCAACGCCATTGATGGGTGAGTATTACCGCCAGCGCGCCAGCGCGGGCCTGATTATCTCCGAAGCCACGCAGATT
TCTGCTCAGGCAAAAGGCTACGCCGGTGCACCGGGTCTGCACAGCCCGGAACAGATCGCCGCGTGGAAAAAAATCACCGCAGGCGTGCATG
CTGAAGATGGCCGTATTGCGGTTCAGCTGTGGCACACCGGTCGTATCTCACACAGCAGCATCCAGCCTGGCGGTCAGGCGCCGGTTTCTGCCT
CTGCCCTGAACGCCAATACCCGCACTTCCCTGCGCGATGAAAACGGTAATGCGATCCGCGTCGACACCACCACGCCACGCGCGCTGGAGCTGG
ACGAGATCCCGGGTATCGTGAATGATTTCCGTCAGGCCGTCGCCAACGCCCGGGAAGCGGGCTTCGACCTGGTTGAGCTTCACTCTGCGCAC
GGTTACCTGCTGCATCAGTTCCTGTCCCCGTCTTCCAACCAGCGTACCGACCAGTACGGCGGCAGCGTTGAAAACCGCGCGCGTCTGGTGCTT
GAAGTGGTGGATGCTGTCTGTAATGAGTGGAGCGCAGACCGCATTGGTATTCGTGTCTCCCCGATCGGTACTTTCCAGAACGTCGACAACGGT
CCGAACGAAGAAGCAGACGCGCTGTATCTGATTGAAGAGCTGGCGAAACGCGGTATCGCCTATCTGCACATGTCCGAGACGGACTTGGCAGG
CGGCAAGCCTTACAGTGAAGCCTTCCGTCAGAAAGTGCGCGAGCGCTTCCACGGCGTGATTATCGGGGCGGGTGCGTATACGGCAGAGAAA
GCCGAGGATTTGATCGGTAAAGGCCTGATCGACGCCGTGGCCTTTGGCCGTGACTACATTGCTAACCCGGATCTGGTTGCCCGTTTGCAGAAA
AAAGCCGAACTGAACCCGCAGCGTCCTGAAAGCTTCTATGGCGGCGGCGCGGAAGGTTATACCGACTACCCTTCACTGCACCACCACCACCAC
CACTGA

pET28a-OYE3

MGSSHHHHHHSSGLVPRGSHNMPFVKGFEPISLRDTNLFEPIKIGNTQLAHRAVMPPLTRMRATHPGNIPNKEWAAVYYGQRAQRPGTMIITEGT
FISPQAGGYDNAPGIWSDEQVAEWKNIFLAIHDCQSFAWVQLWSLGWASFPDVLARDGLRYDCASDRVYMNATLQEKAKDANNLEHSLTKDDIK
QYIKDYIHAAKNSIAAGADGVEIHSANGYLLNQFLDPHSNKRTDEYGGTIENRARFTLEVVDALIETIGPERVGLRLSPYGTFNSMSGGAEPGIIAQYSY
VLGELEKRAKAGKRLAFVHLVEPRVTDPSLVEGEGEYSEGTNDFAYSIWKGPIIRAGNYALHPEVVREQVKDPRTLIGYGRFFISNPDLVYRLEEGLPLN
KYDRSTFYTMSAEGYTDYPTYEEAVDLGWNKN

ATGGGCAGCAGCCATCATCATCATCATCACAGCAGCGGCCTGGTGCCGCGCGGCAGCCATAATATGCCATTTGTAAAAGGTTTTGAGCCGATC
TCCCTAAGAGACACAAACCTTTTTGAACCAATTAAGATTGGTAACACTCAGCTTGCACATCGTGCGGTTATGCCCCCATTGACCAGAATGAGGG
CCACTCACCCCGGAAATATTCCAAATAAGGAGTGGGCTGCTGTGTATTATGGTCAGCGTGCTCAAAGACCTGGTACCATGATCATCACGGAAG
GTACGTTTATTTCCCCTCAAGCCGGCGGCTATGACAACGCCCCTGGGATTTGGTCTGATGAGCAGGTCGCTGAGTGGAAGAATATCTTTTTAG
CCATCCATGATTGTCAGTCGTTCGCGTGGGTACAACTTTGGTCTTTAGGCTGGGCATCCTTCCCAGACGTATTGGCAAGAGACGGGTTACGCTA
TGACTGTGCATCTGACAGAGTGTATATGAATGCTACGTTACAAGAAAAGGCCAAAGATGCGAATAATCTCGAACATAGTTTGACTAAAGACGA
CATTAAACAGTATATCAAGGATTACATCCATGCGGCTAAGAATTCTATCGCGGCTGGCGCCGATGGTGTAGAAATTCATAGCGCCAATGGGTA
CTTGTTGAATCAGTTCTTGGACCCACATTCTAATAAGAGGACCGACGAATACGGCGGAACGATCGAAAACAGGGCCCGCTTTACACTGGAGGT
TGTCGATGCTCTTATCGAAACTATCGGTCCTGAACGGGTGGGTTTGAGGTTGTCGCCGTACGGCACTTTTAACAGTATGTCTGGGGGTGCTGA
ACCAGGTATTATCGCTCAATATTCGTATGTTTTGGGTGAATTAGAGAAGAGGGCAAAGGCTGGTAAGCGTTTGGCCTTTGTGCACCTCGTTGA
ACCACGTGTCACGGACCCATCGTTGGTGGAGGGCGAAGGAGAATATTCCGAGGGTACTAACGATTTTGCCTACTCTATATGGAAGGGTCCAA
TCATCAGAGCTGGTAATTACGCTCTTCATCCAGAAGTGGTTAGAGAACAAGTAAAGGACCCCAGAACCTTGATAGGCTATGGTAGATTCTTCA
TCTCTAACCCAGATTTAGTCTACCGTTTAGAAGAGGGCCTGCCATTGAACAAGTATGACAGAAGTACCTTCTACACCATGTCCGCGGAAGGTTA
TACCGACTACCCAACGTATGAAGAGGCAGTAGATTTAGGTTGGAACAAGAACTGA

HPLC chromatograms

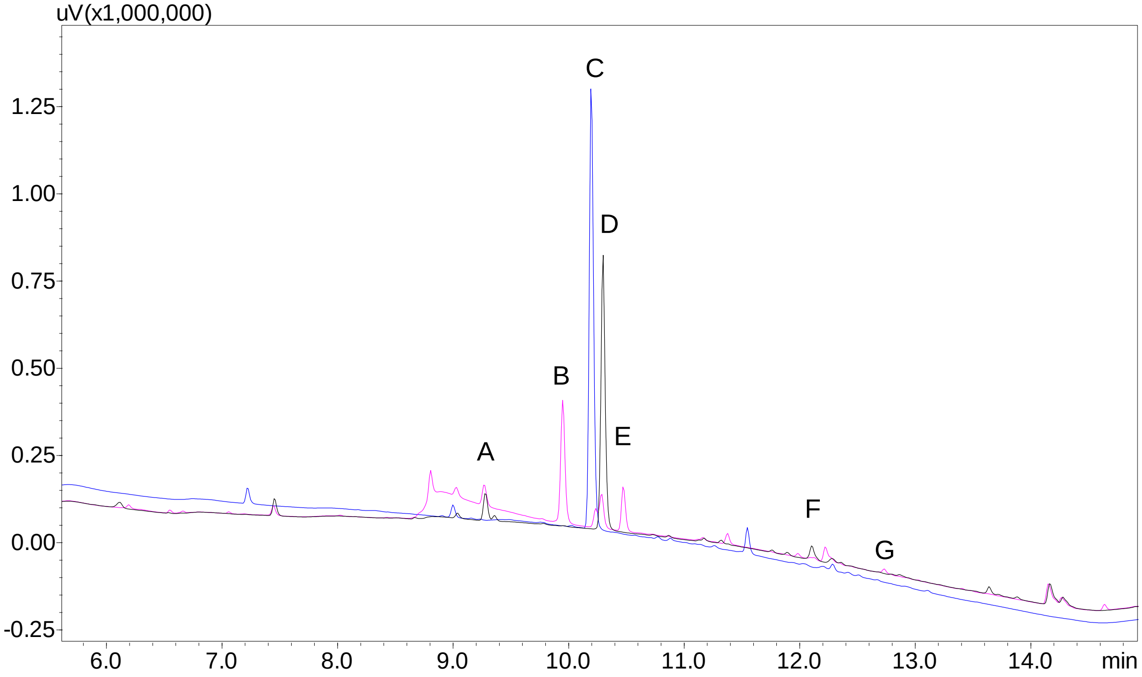

Figure S8: Stacked HPLC chromatograms of initial reactions employing (partially oxidized/polymerized) **2a** and an excess (3eq.) of **1a**. **Black**: no ERED, **pink**: GluER T36A (NaCl in enzyme preparation); **blue**: standard of **6a**. Peak assignment (based on standards): A: **4a**, B: Cl-**1a**, C: **6a**, D: **1a**, E: putative **5aa**, F: **2a**, G: **3aa**. N.B. in this early reaction, the HPLC sample was prepared with TFA (0.1%), which caused hydrolysis of **2a**.

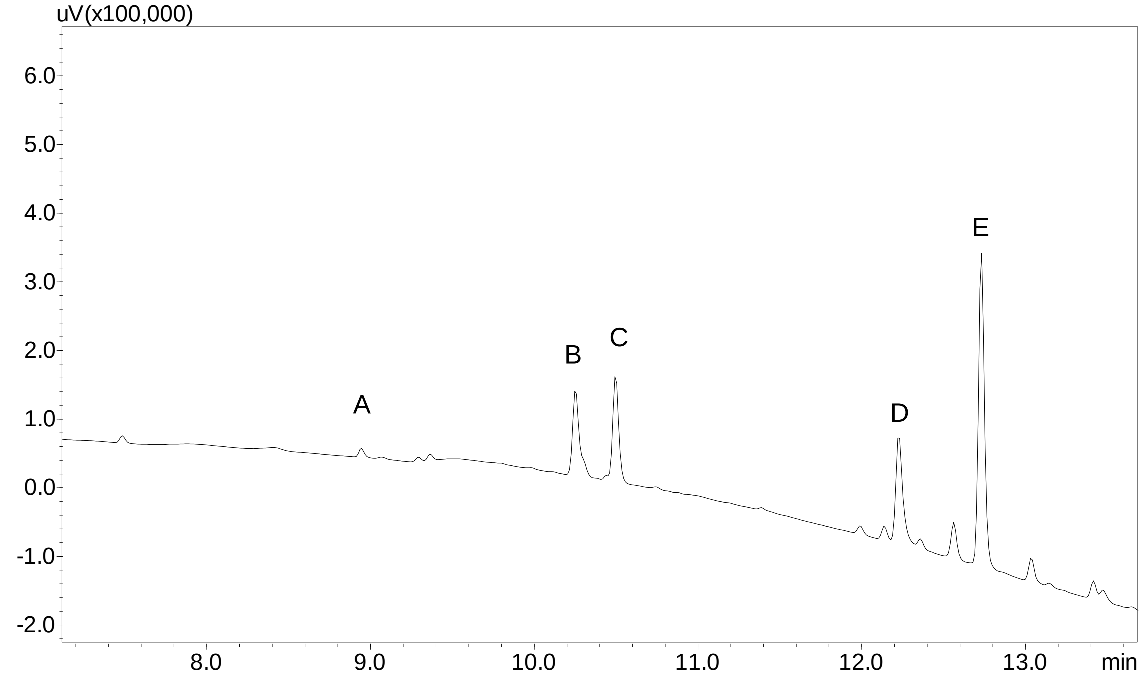

Figure S9: **3aa** (10 mM) incubated with **1a** (10 mM) under reaction conditions, without ERED. A: **4a**, B: **1a**, C: putative **5aa**, D: unknown, E: **3aa**.

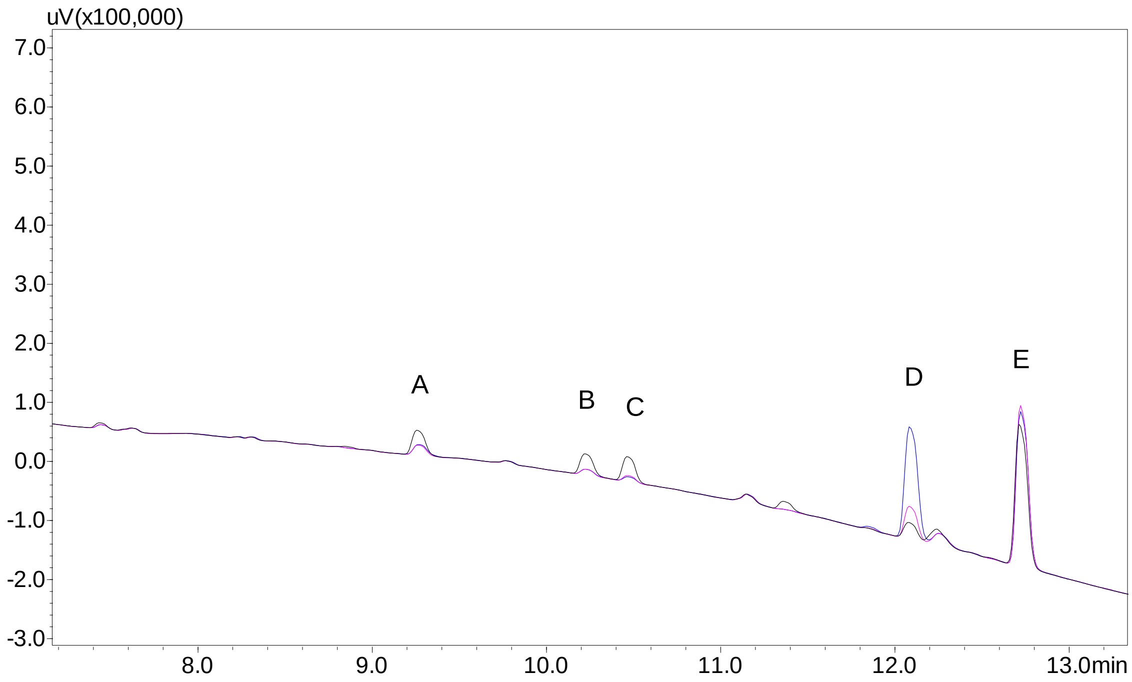

Figure S10: Stacked HPLC chromatograms of reactions employing different amounts of **2a** and **1a**. **Black**: 1.5 eq. **1a**, **pink**: Equal amounts **1a** and **2a**, **blue**: 1.5 eq. **2a**. Peak assignment (based on standards): A: **4a**, B: **1a**, C: putative **5aa**, D: **2a**, E: **3aa**.

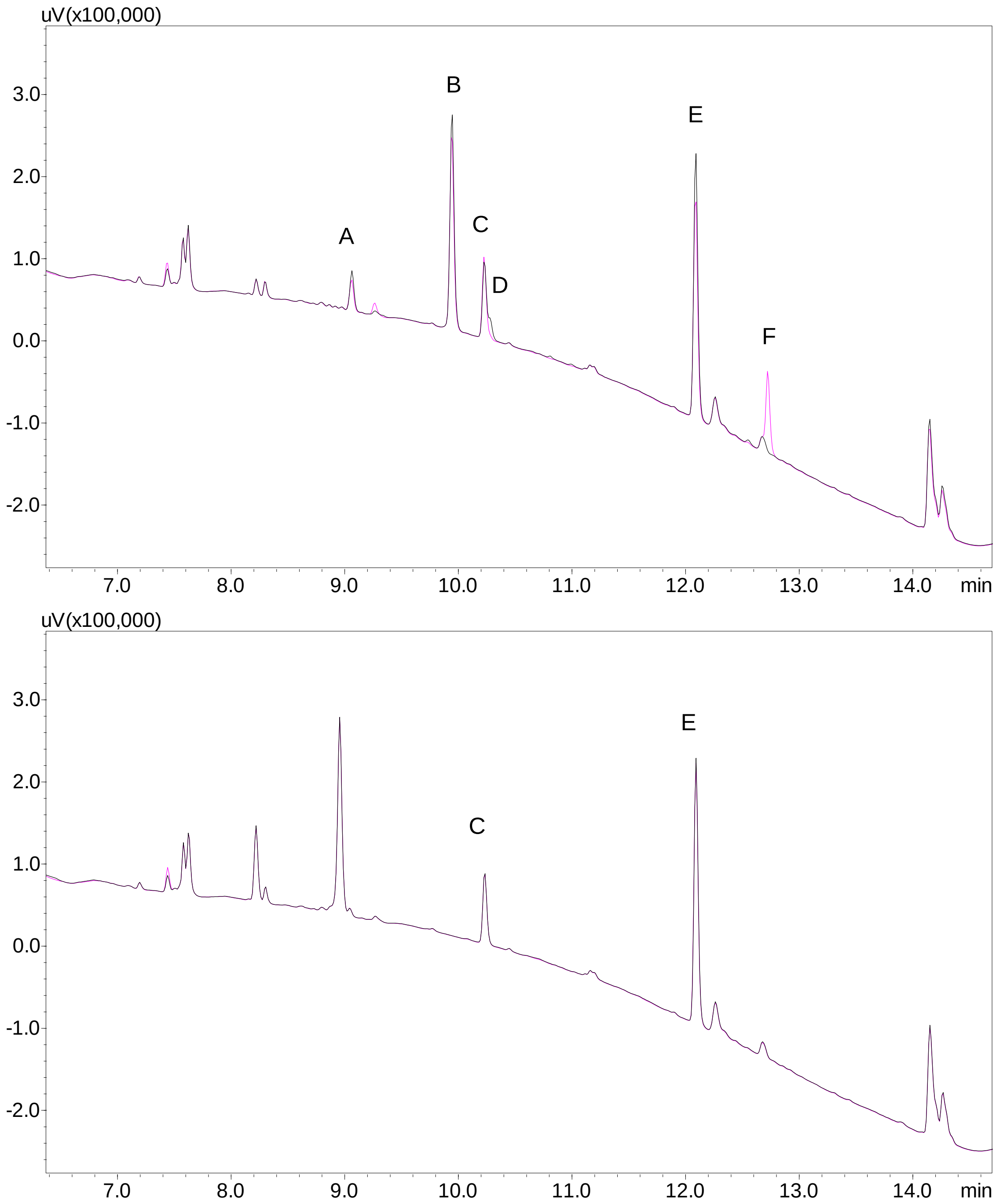

Figure S11: **Top:** Stacked HPLC chromatograms of initial reactions employing (partially oxidized/polymerized) **2a** (3 eq.) and Cl-**1a**. **Black:** no ERED, **pink:** GluER T36A; **Bottom:** Stacked HPLC chromatograms of initial reactions employing (partially oxidized/polymerized) **2a** (3 eq.) and F-**1a**. **Black:** no ERED, **pink:** GluER T36A; Peak assignment (based on standards): A: **4a**, B: Cl-**1a**, C: **6a**, D: **1a**, E: **2a**. F: **3aa**, G: F-**2a**.

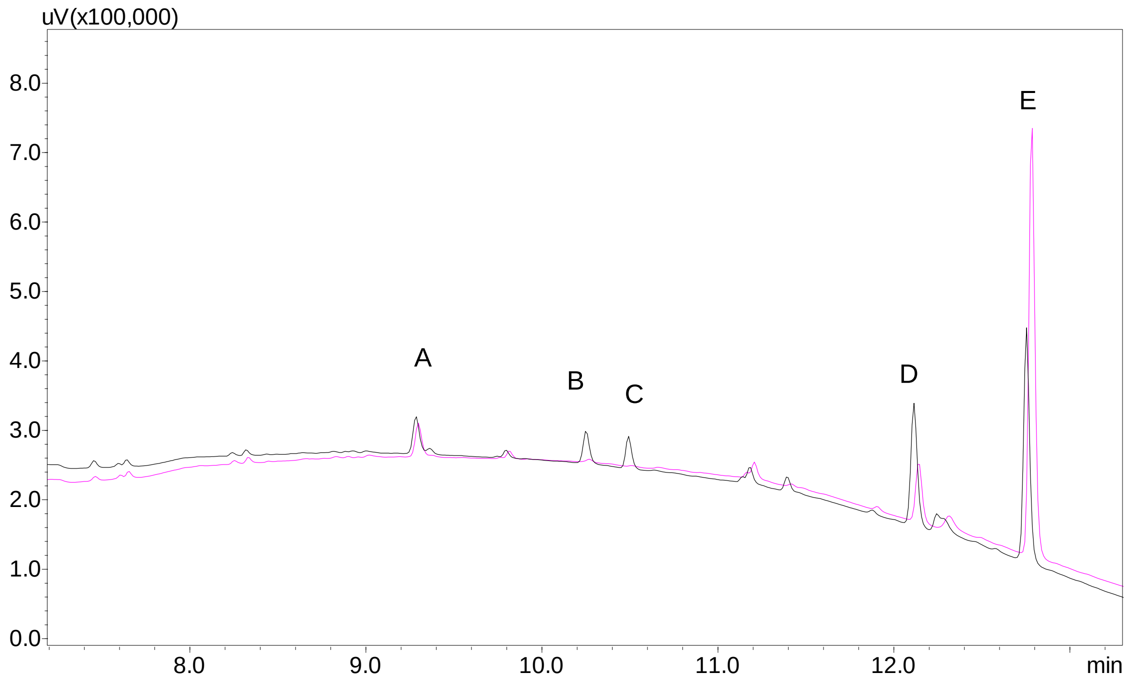

Figure S12: Stacked HPLC chromatograms of reactions of **1a** and **2a,** employing different amounts of GluER T36A. **Black:** 0.8 mol% GluER T36A, **pink:** 2 mol% GluER T36A. Peak assignment (based on standards): A: **4a**, B: **6a**, C: putative **5aa**, D: **2a**, E: **3aa**.

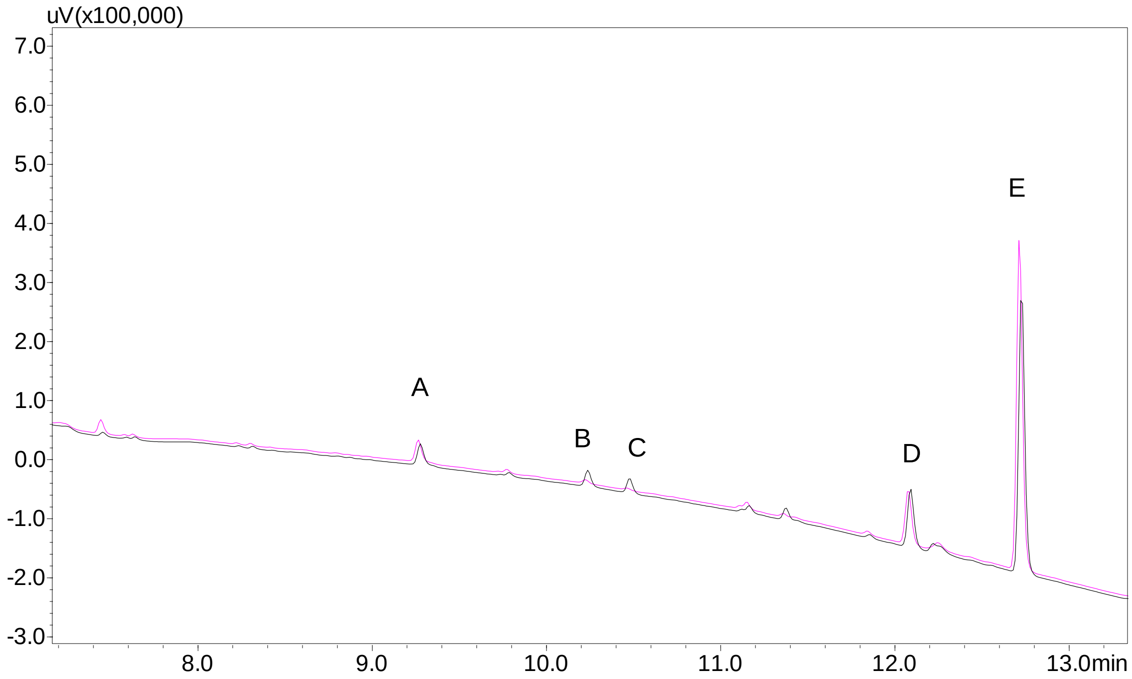

Figure S13: Stacked HPLC chromatograms of reactions of **1a** and **2a** at 20 mM scale, employing different amounts of GluER T36A. **Black:** 0.7 mol% GluER T36A, **pink:** 1.4 mol% GluER T36A. Peak assignment (based on standards): A: **4a**, B: **6a**, C: putative **5aa**, D: **2a**, E: **3aa**.

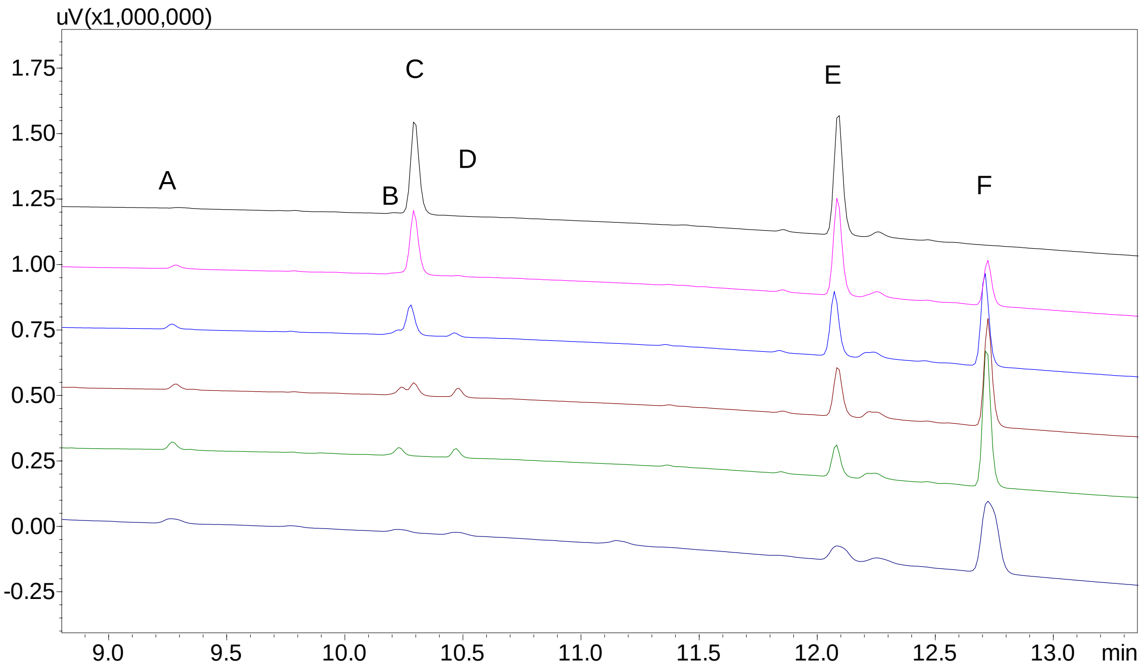

Figure S14: Stacked HPLC chromatograms of a time course of reactions of **1a** and **2a** with GluER T36A. **Black**: t=0h, **pink**: t=1h, **blue**: t=3h, **brown**: t=7h, **green**: t=16h, **dark blue**: t=24h. Peak assignment (based on standards): A: **4a**, B: **6a**, C: **1a**, D: putative **5aa**, E: **2a**, F: **3aa**.

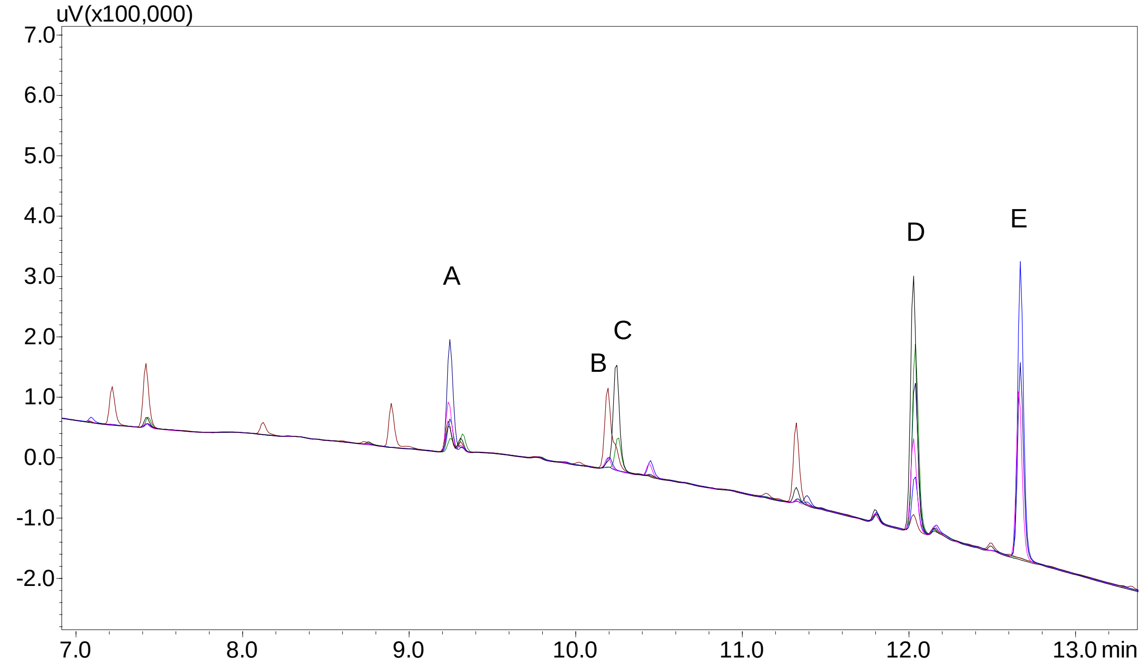

Figure S15: Stacked HPLC chromatograms of reactions of **1a** and **2a**, employing different EREDs. **Black:** FMN, **pink:** NCR, **blue:** GluER T36A, **brown:** GluER T36A in the presence of oxygen, **green**: OYE3, **dark blue**: PETNR. Peak assignment (based on standards): A: **4a**, B: **6a**, C: **1a**, D: **2a**, E: **3aa**.

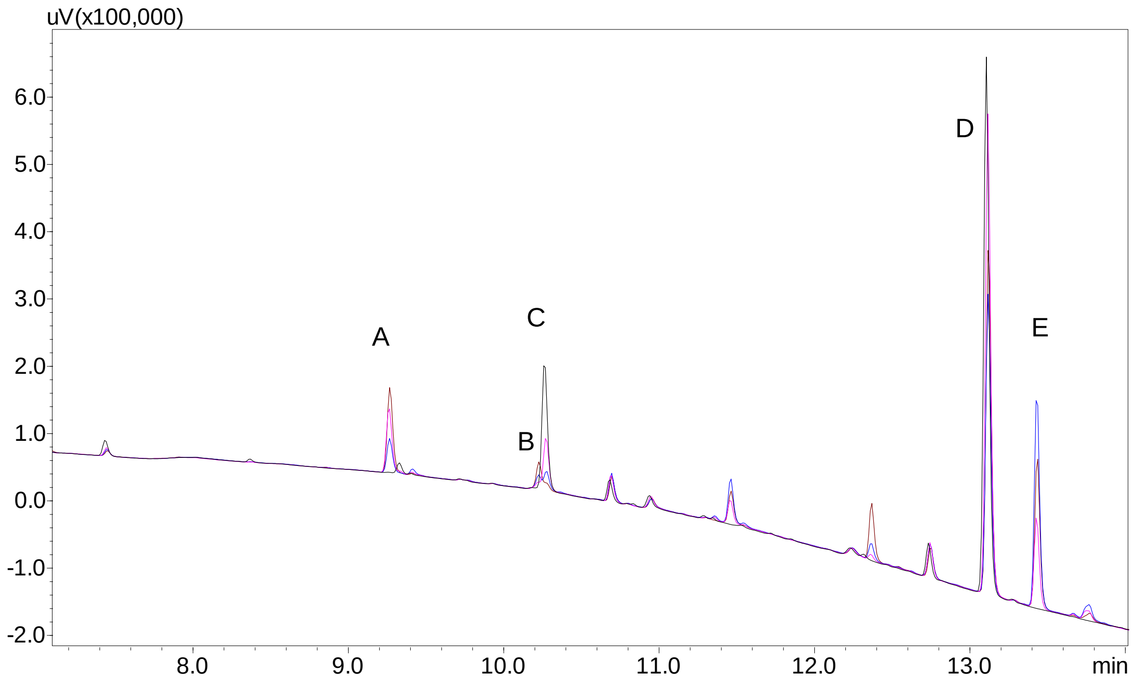

Figure S16: Stacked HPLC chromatograms of reactions of **1a** and **2b**, employing different EREDs. **Black:** none, **pink:** NCR, **blue:** GluER T36A, **brown:** PETNR. Peak assignment (based on standards): A: **4a**, B: **6a**, C: **1a**, D: **2b**, E: **3ba**.

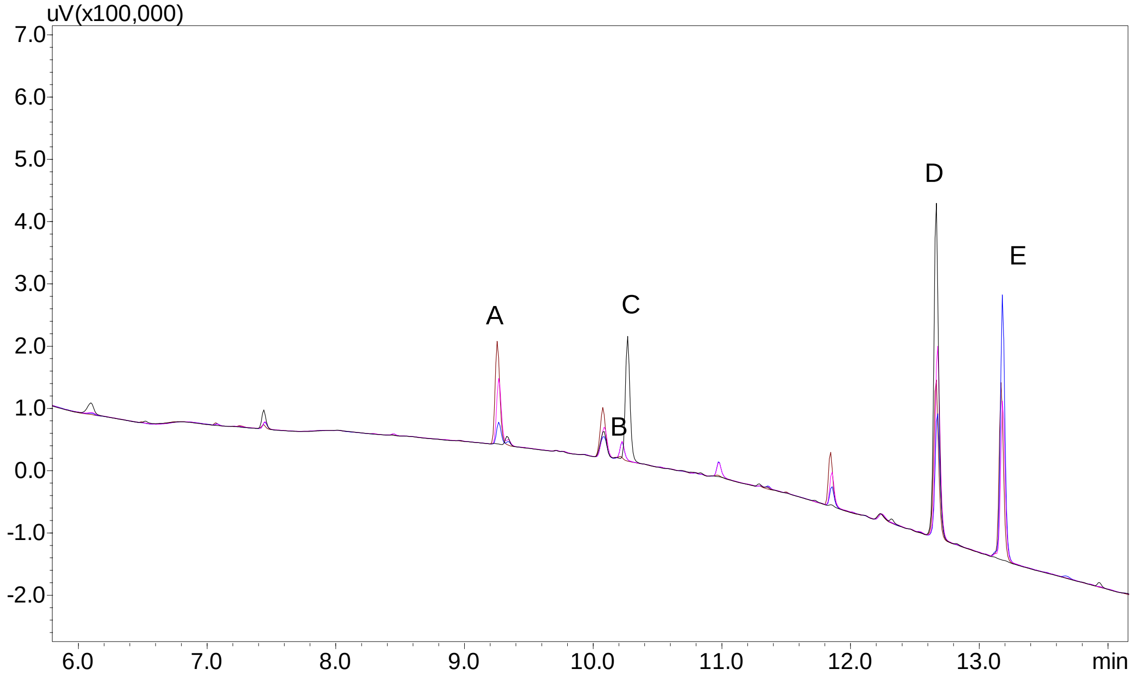

Figure S17: Stacked HPLC chromatograms of reactions of **1a** and **2c**, employing different EREDs. **Black:** none, **pink:** NCR, **blue:** GluER T36A, **brown:** PETNR. Peak assignment (based on standards): A: **4a**, B: **6a**, C: **1a**, D: **2c**, E: **3ca**.

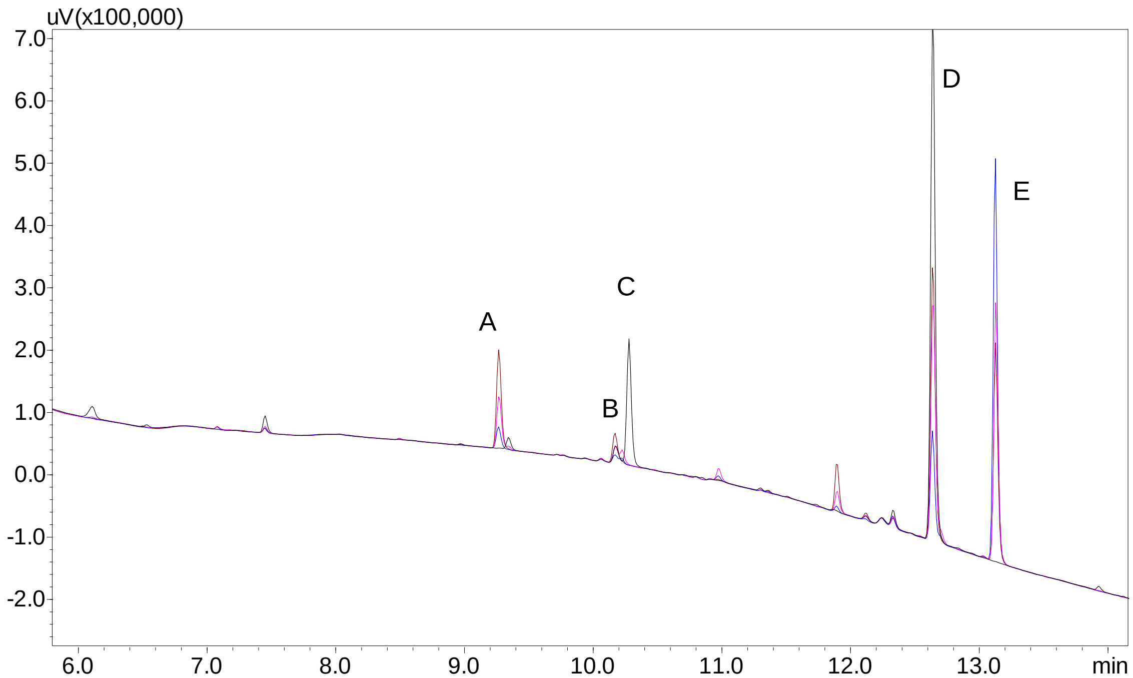

Figure S18: Stacked HPLC chromatograms of reactions of **1a** and **2d**, employing different EREDs. **Black**: none, **pink**: NCR, **blue**: GluER T36A, **brown**: PETNR. Peak assignment (based on standards): A: **4a**, B: **6a**, C: **1a**, D: **2d**, E: **3da**.

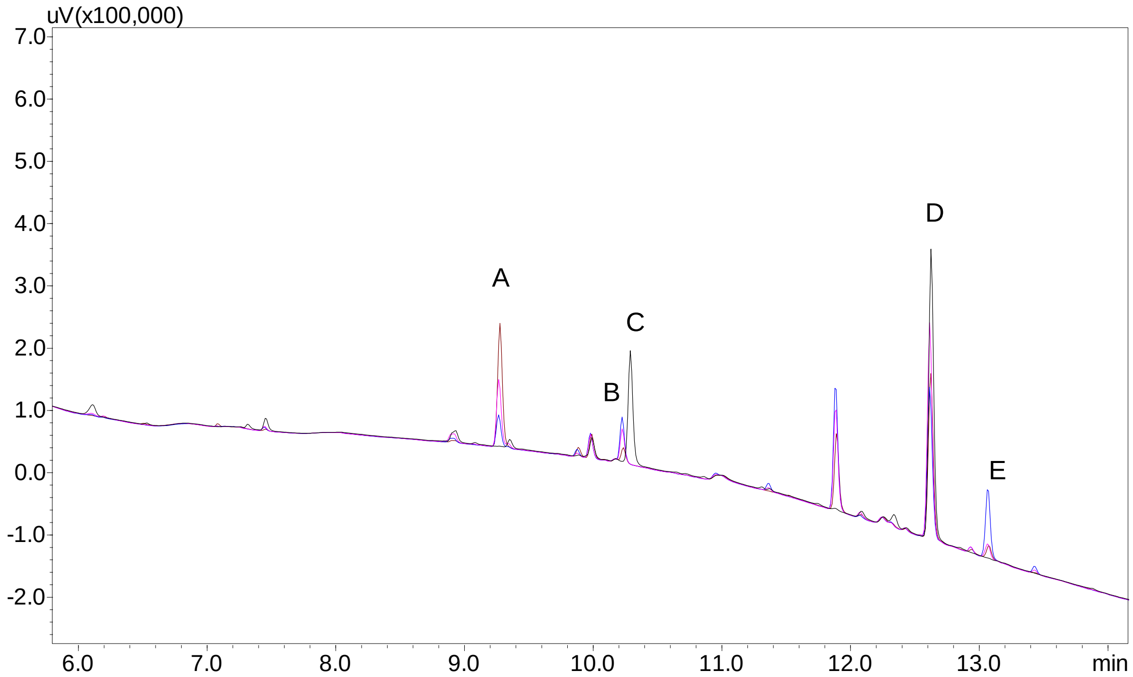

Figure S19: Stacked HPLC chromatograms of reactions of **1a** and **2e**, employing different EREDs. **Black**: none, **pink**: NCR, **blue**: GluER T36A, **brown**: PETNR. Peak assignment (based on standards): A: **4a**, B: **6a**, C: **1a**, D: **2e**, E: **3ea**.

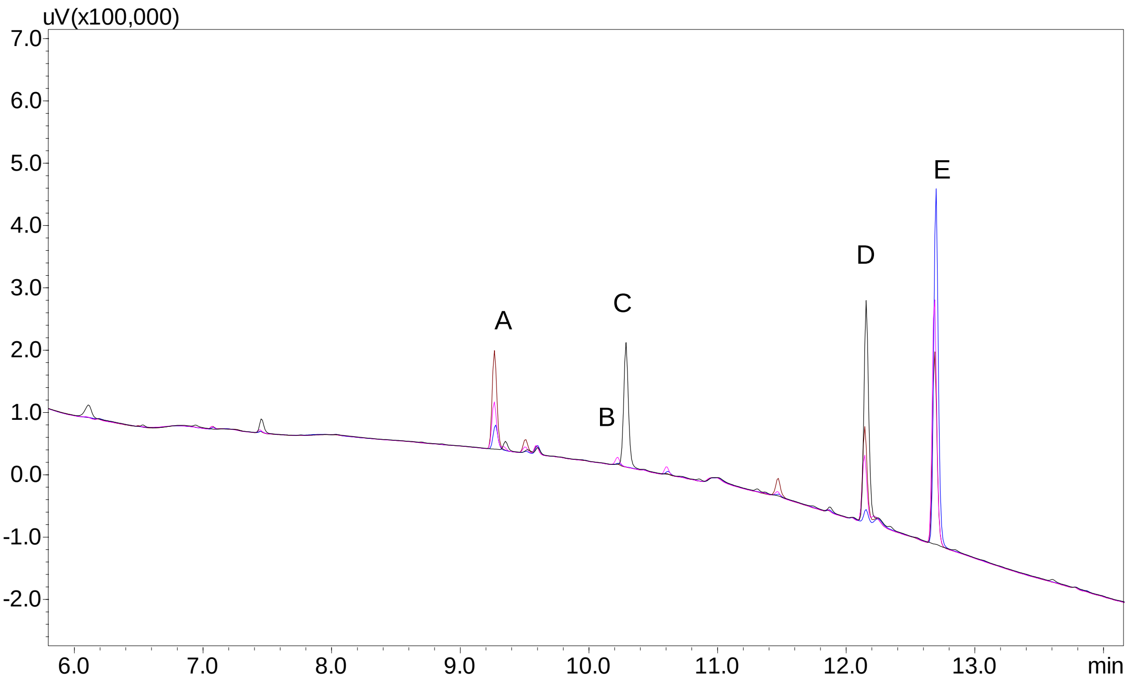

Figure S20: Stacked HPLC chromatograms of reactions of **1a** and **2f**, employing different EREDs. **Black**: none, **pink**: NCR, **blue**: GluER T36A, **brown**: PETNR. Peak assignment (based on standards): A: **4a**, B: **6a**, C: **1a**, D: **2f**, E: **3fa**.

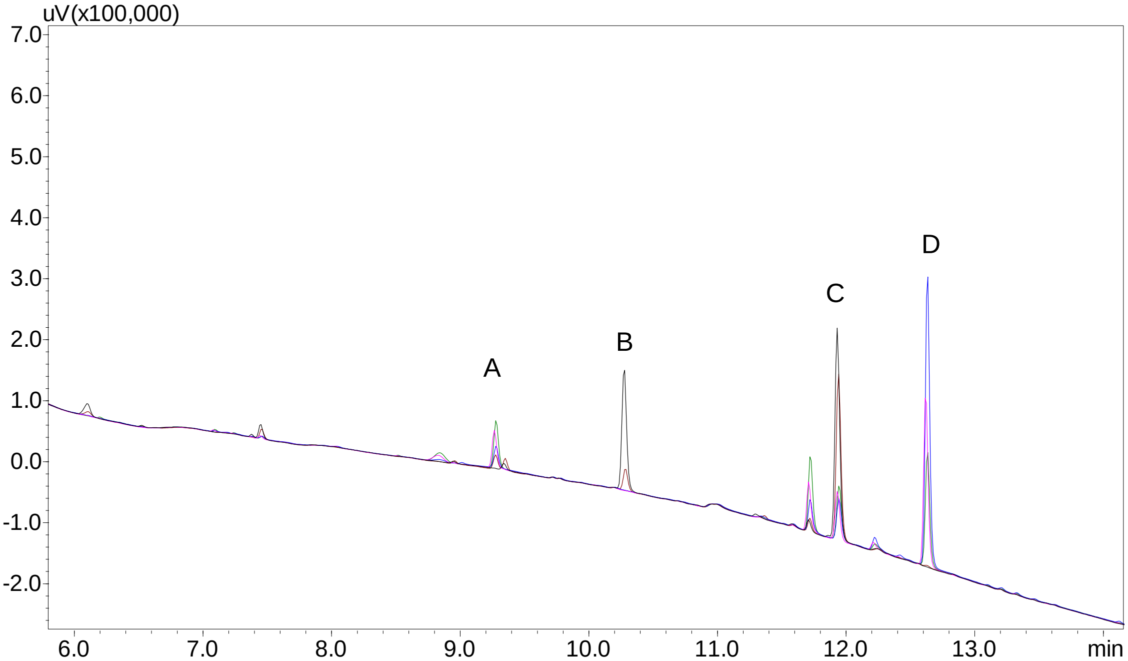

Figure S21: Stacked HPLC chromatograms of reactions of **1a** and **2g**, employing different EREDs. **Black**: none, **pink**: NCR, **blue**: GluER T36A, **brown**: OYE3, **green**: PETNR. Peak assignment (based on standards): A: **4a**, B: **1a**, C: **2g**, D: **3ga**.

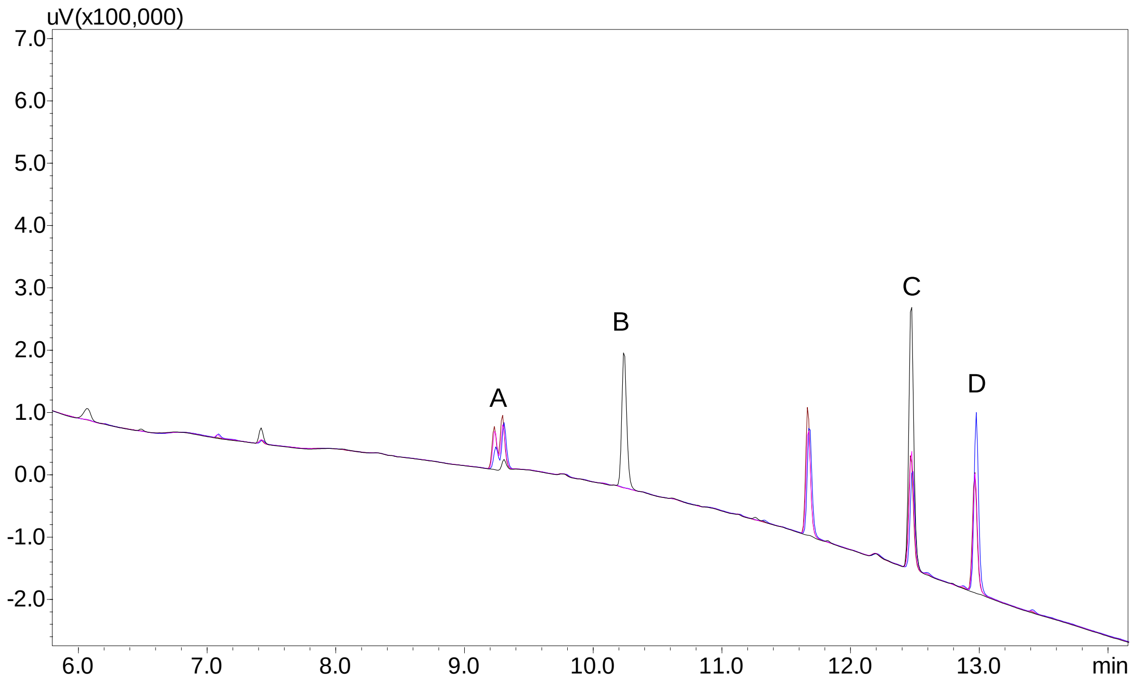

Figure S22: Stacked HPLC chromatograms of reactions of **1a** and **2h**, employing different EREDs. **Black**: none, **pink**: NCR, **blue**: GluER T36A, **brown**: PETNR. Peak assignment (based on standards): A: **4a**, B: **1a**, C: **2h**, D: **3ha**.

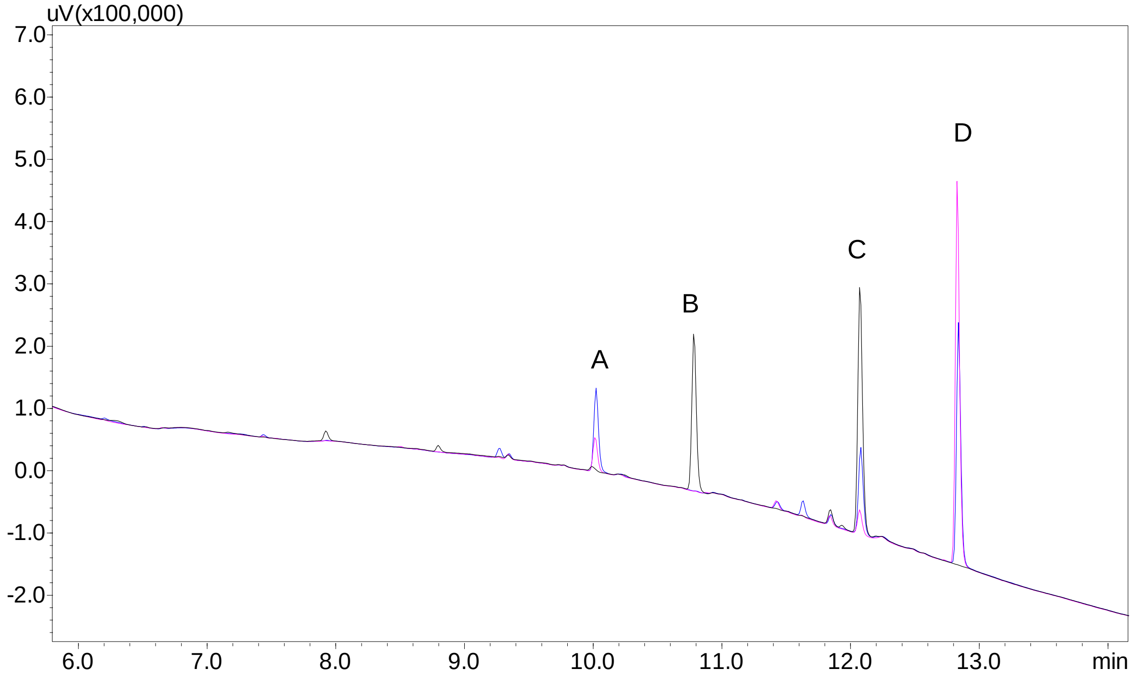

Figure S23: Stacked HPLC chromatograms of reactions of **1b** and **2a**, employing different EREDs. **Black**: none, **pink**: GluER T36A, **blue**: PETNR. Peak assignment (based on standards): A: **4b**, C: **1b**, C: **2a**, D: **3ab**.

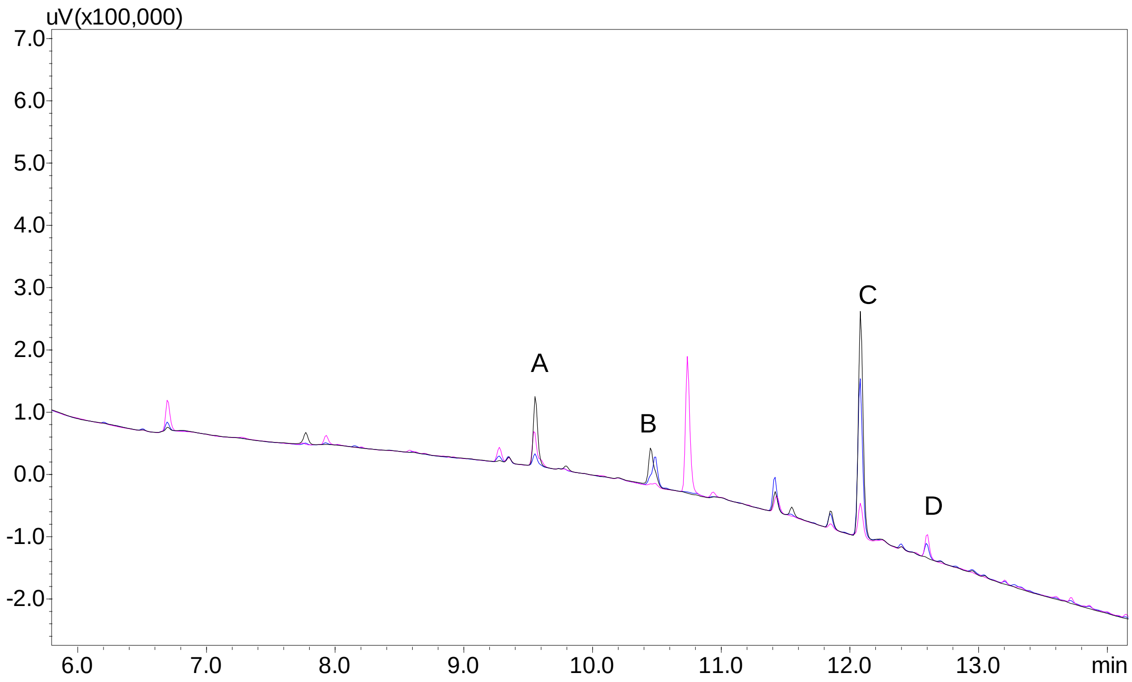

Figure S24: Stacked HPLC chromatograms of reactions of **1c** and **2a**, employing different EREDs. **Black**: none, **pink**: GluER T36A, **blue**: PETNR. Peak assignment (based on standards): A: **4c**, B: **1c**, C: **2a**, D: **3ac**.

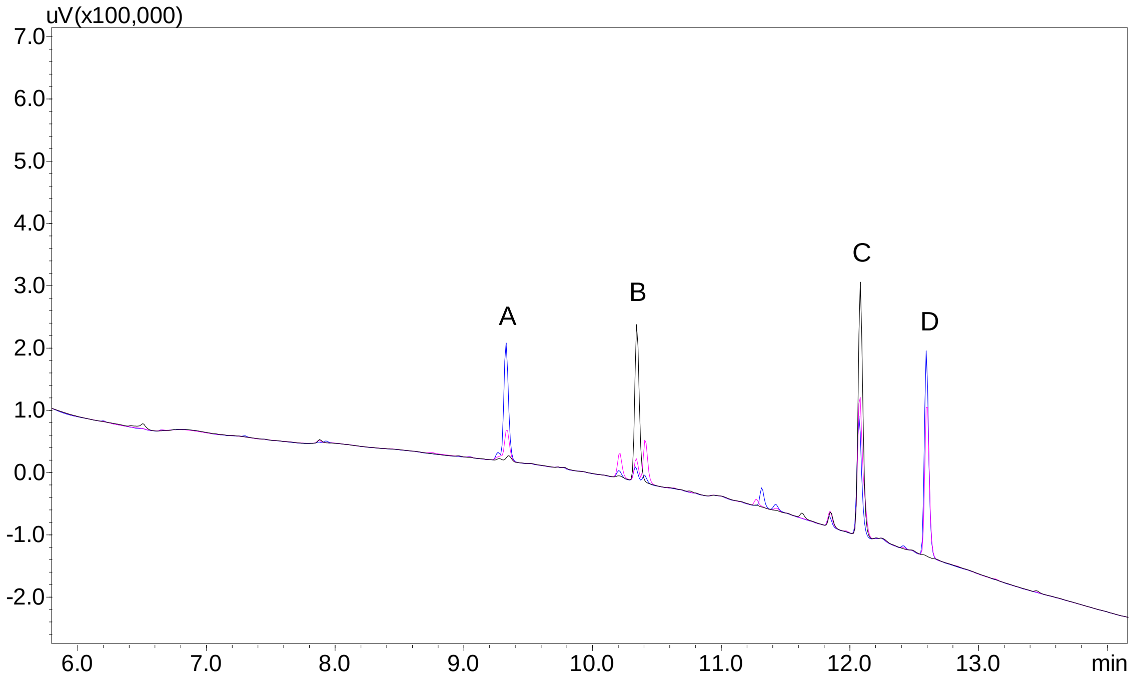

Figure S25: Stacked HPLC chromatograms of reactions of **1d** and **2a**, employing different EREDs. **Black**: none, **pink**: GluER T36A, **blue**: PETNR. Peak assignment (based on standards): A: **4d**, B: **1d**, C: **2a**, D: **3ad**.

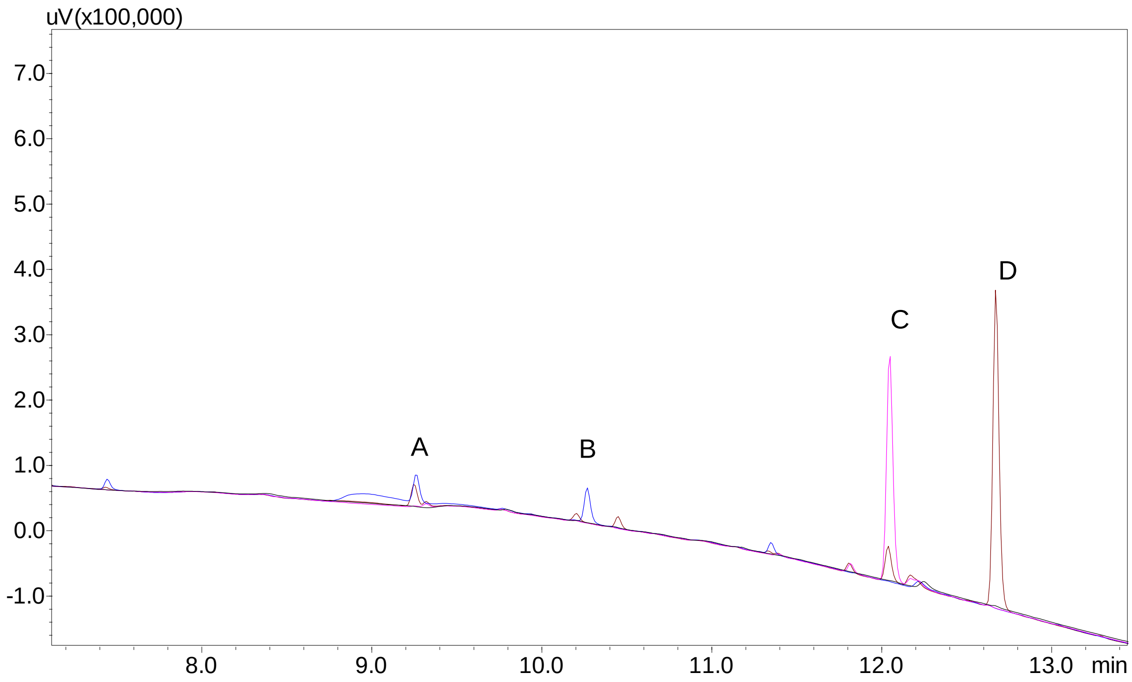

Figure S26: Stacked HPLC chromatograms of reactions of **1a** and **2a** with GluER T36A, leaving out either or both substrates. **Black**: neither substrate added, **pink**: only **2a** added, **blue**: only **1a** added, **brown**: both **1a** and **2a** added. Peak assignment (based on standards): A: **4a**, **C**: **1a**, C: **2a**, D: **3aa**.

Chiral HPLC chromatograms

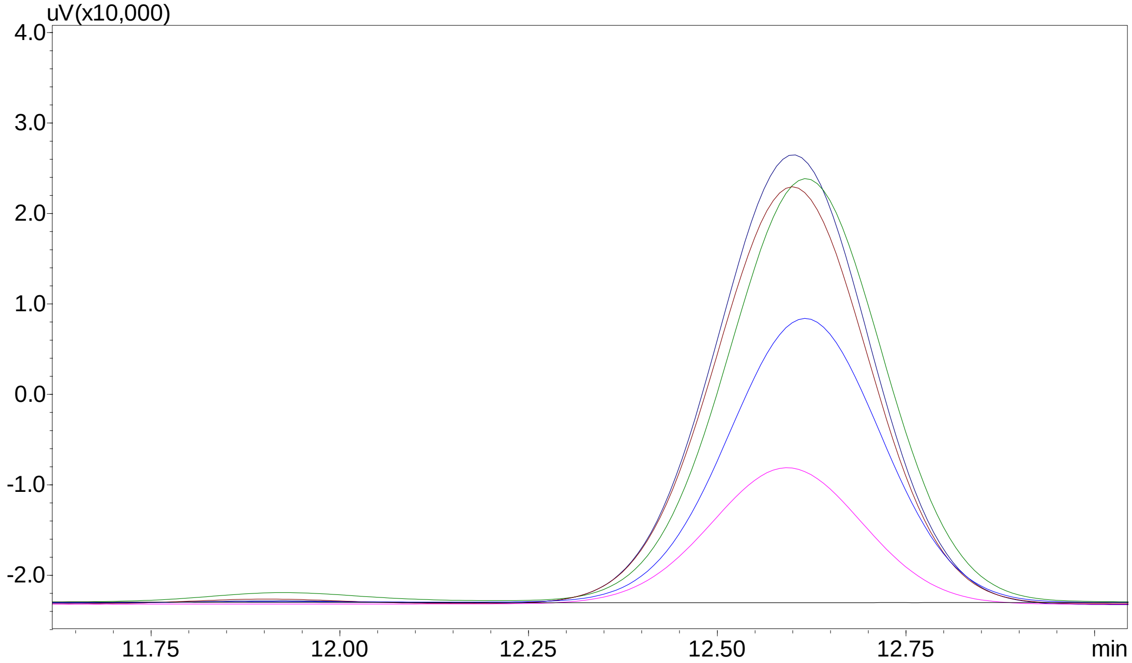

Figure S27: Stacked chiral HPLC chromatograms of reactions of **3aa**, from a time course with GluER T36A. **Black**: t=0h, **pink**: t=1h, **blue**: t=3h, **brown**: t=7h, **green**: t=16h, **dark blue**: t=24h.

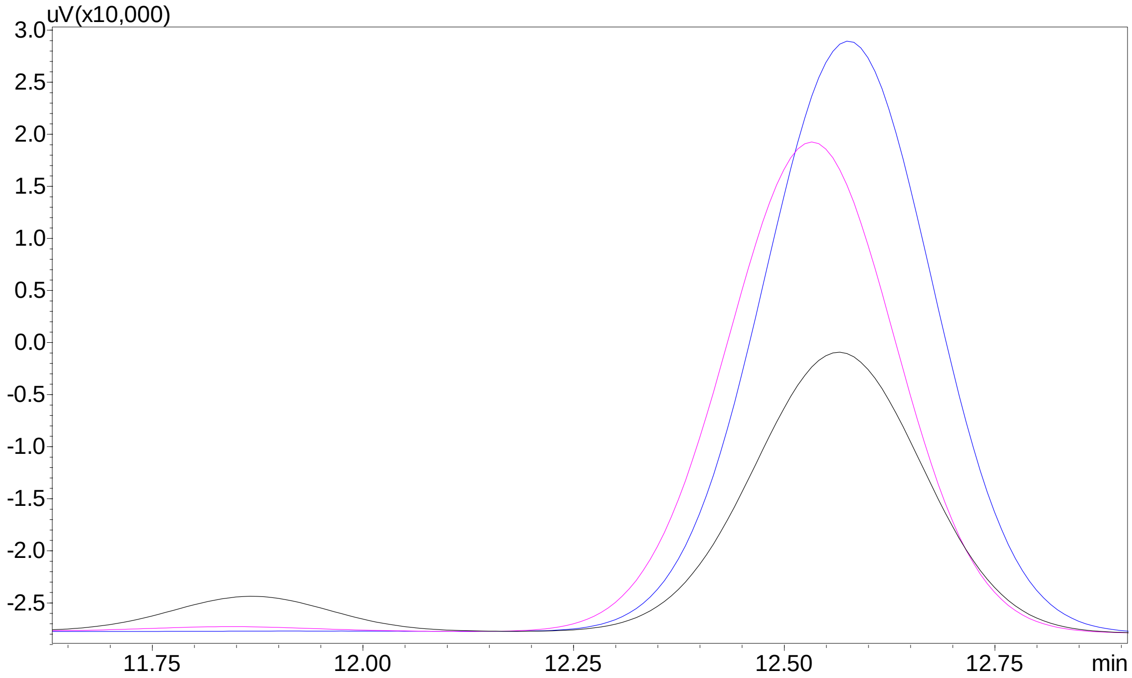

Figure S28: Stacked chiral HPLC chromatograms of reactions of **3aa**, with different amounts of GluER T36A. **Black**: 0.8 mol%, **pink**: 1.4 mol%, **blue**: 2.0 mol%.

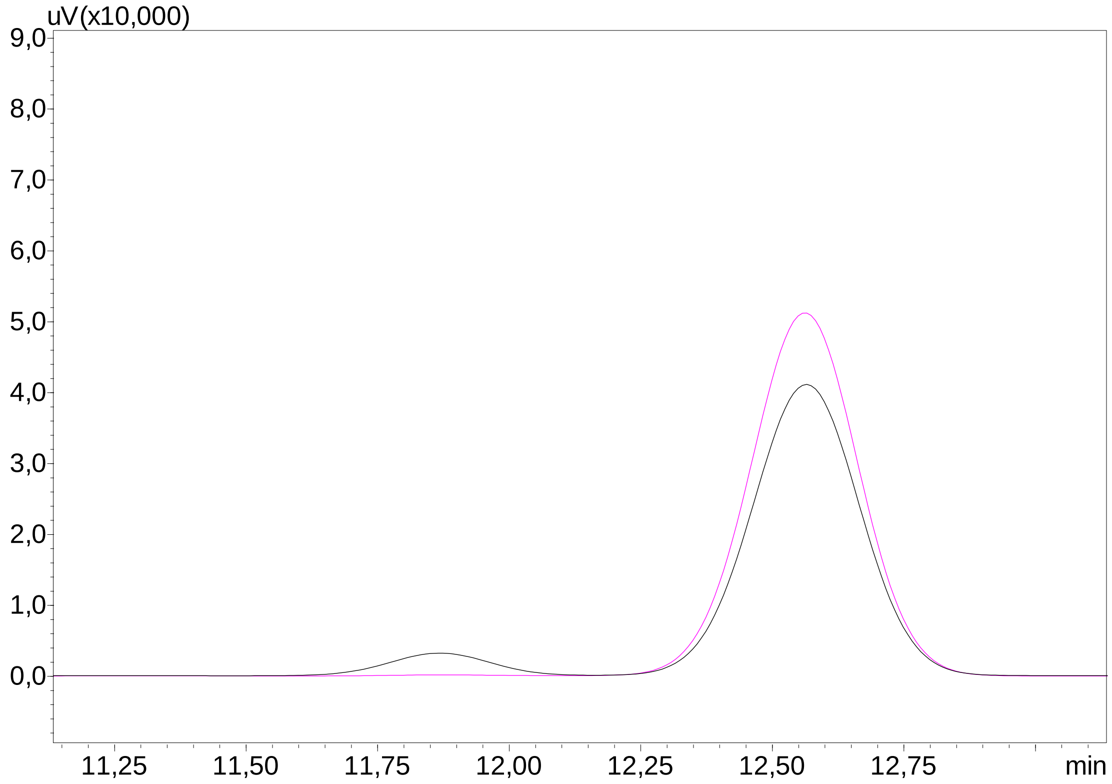

Figure S29: Stacked chiral HPLC chromatograms of reactions of **3aa** at 20 mM scale, with different amounts of GluER T36A. **Black**: 0.7 mol%, **pink**: 1.4 mol%.

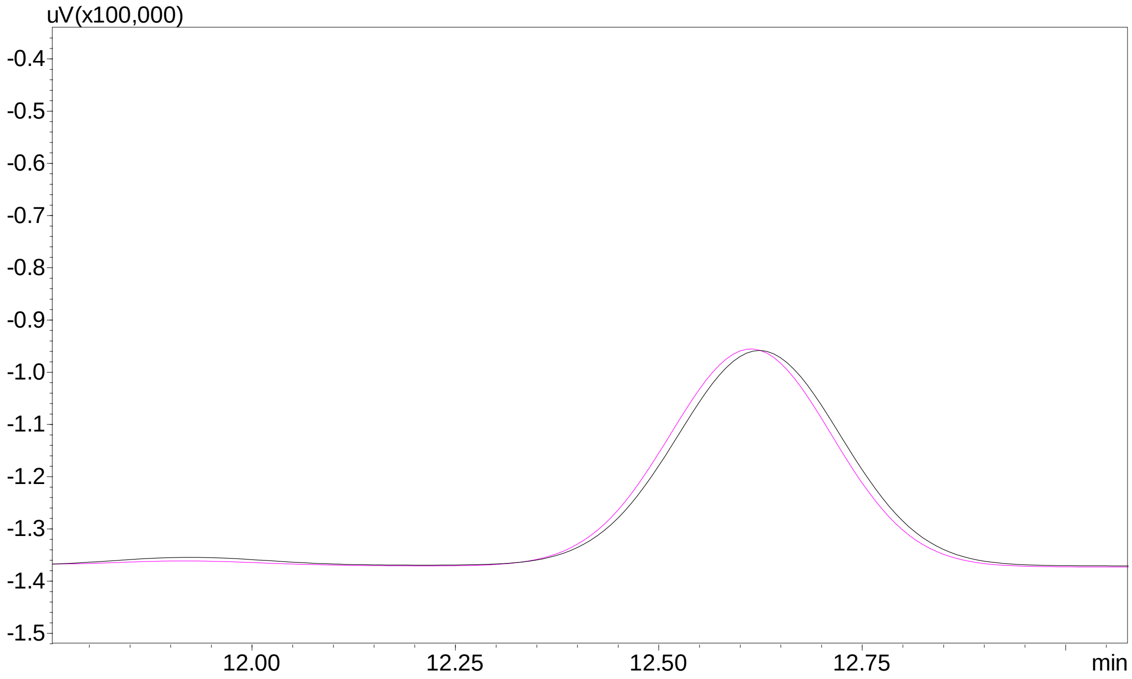

Figure S30: Stacked chiral HPLC chromatograms of isolated **3aa** (10 mM) incubated with **1a** (10 mM) under reaction conditions, without ERED. **Black**: isolated **3aa**. **Pink**: **3aa** incubated with **1a**.

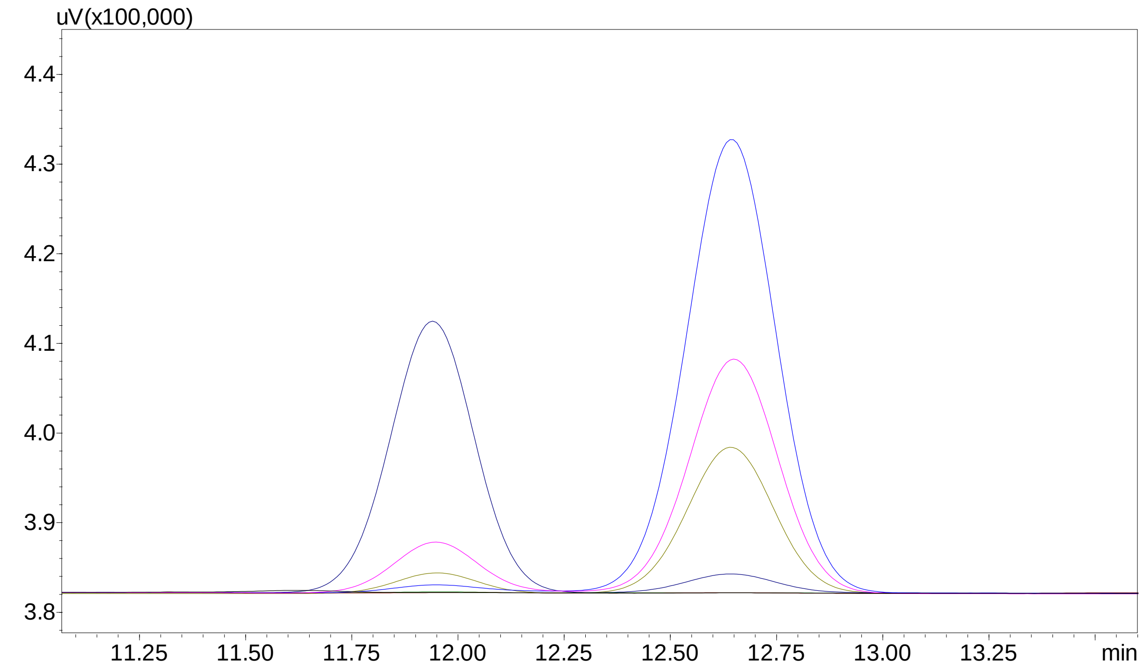

Figure S31: Stacked chiral HPLC chromatograms of reactions of **3aa**, from reactions with different EREDs. **Black**: FMN, **Pink**: NCR, **blue**: GluER T36A, **brown**: GluER T36A in the presence of oxygen, **green**: OYE3, **dark blue**: PETNR, **olive**: purified compound of NCR reaction.

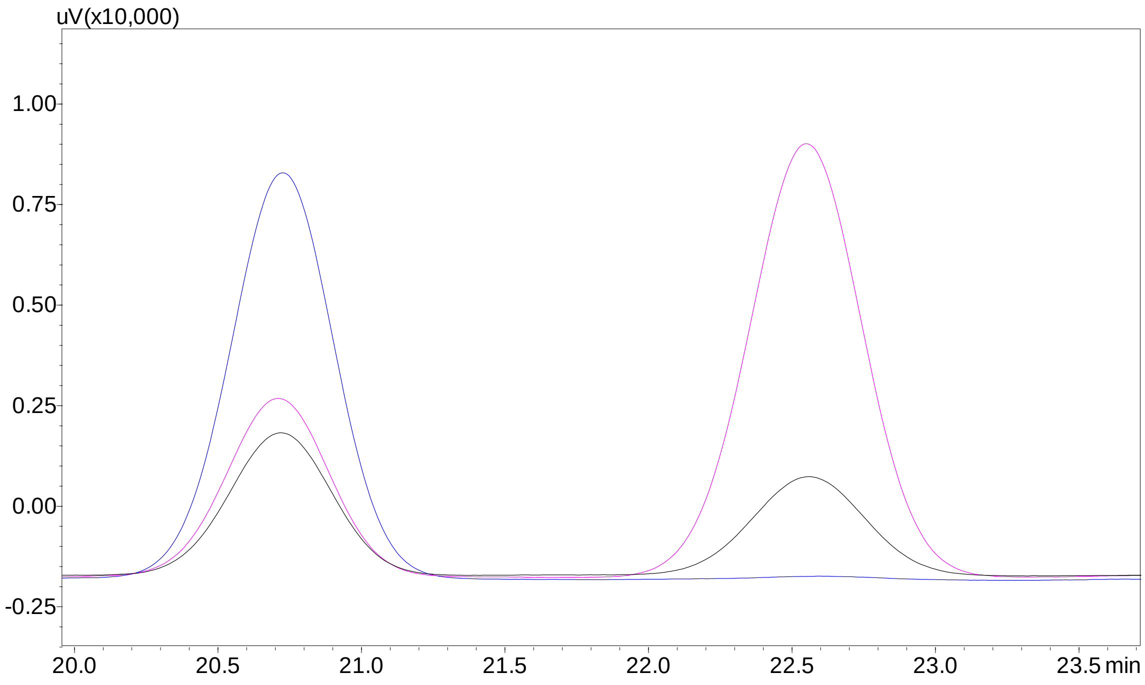

Figure S32: Stacked chiral HPLC chromatograms of reactions of **3ba**, from reactions with different EREDs. **Black**: NCR, **pink**: GluER T36A, **blue**: PETNR.

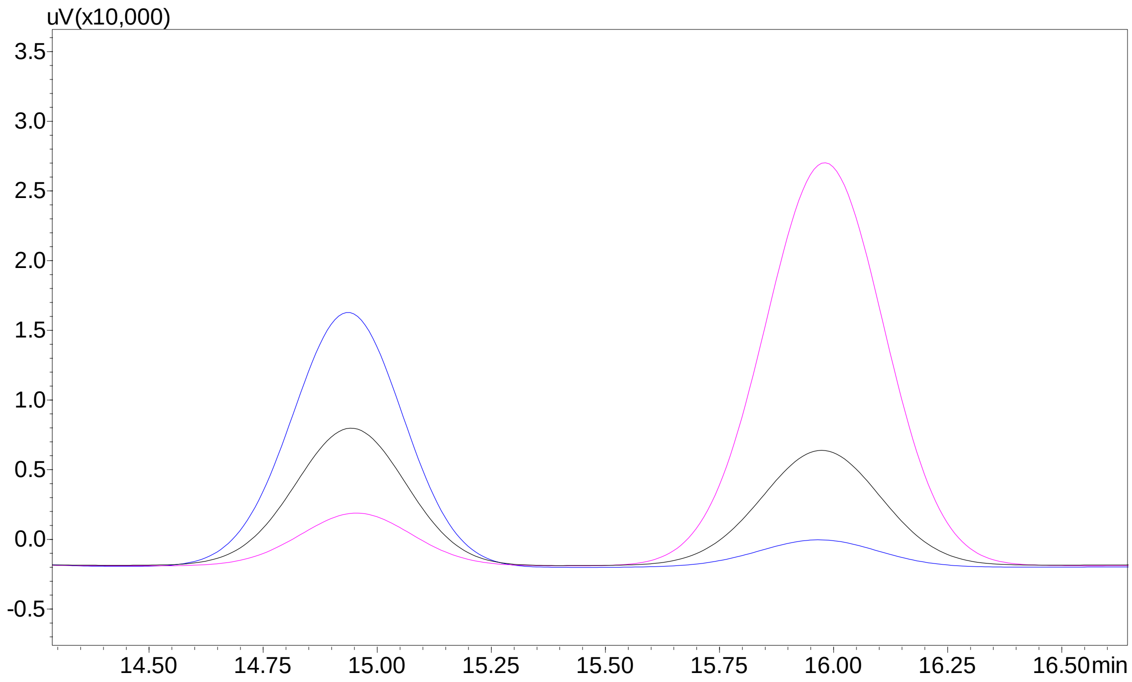

Figure S33: Stacked chiral HPLC chromatograms of reactions of **3ca**, from reactions with different EREDs. **Black**: NCR, **pink**: GluER T36A, **blue**: PETNR.

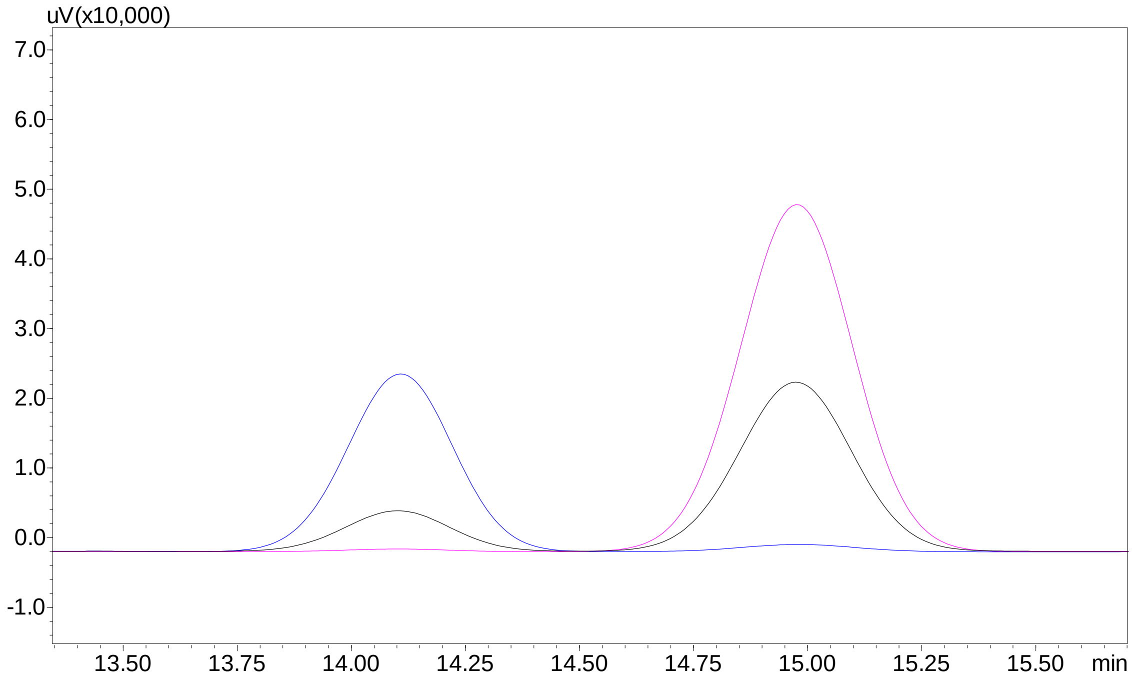

Figure S34: Stacked chiral HPLC chromatograms of reactions of **3da**, from reactions with different EREDs. **Black**: NCR, **pink**: GluER T36A, **blue**: PETNR.

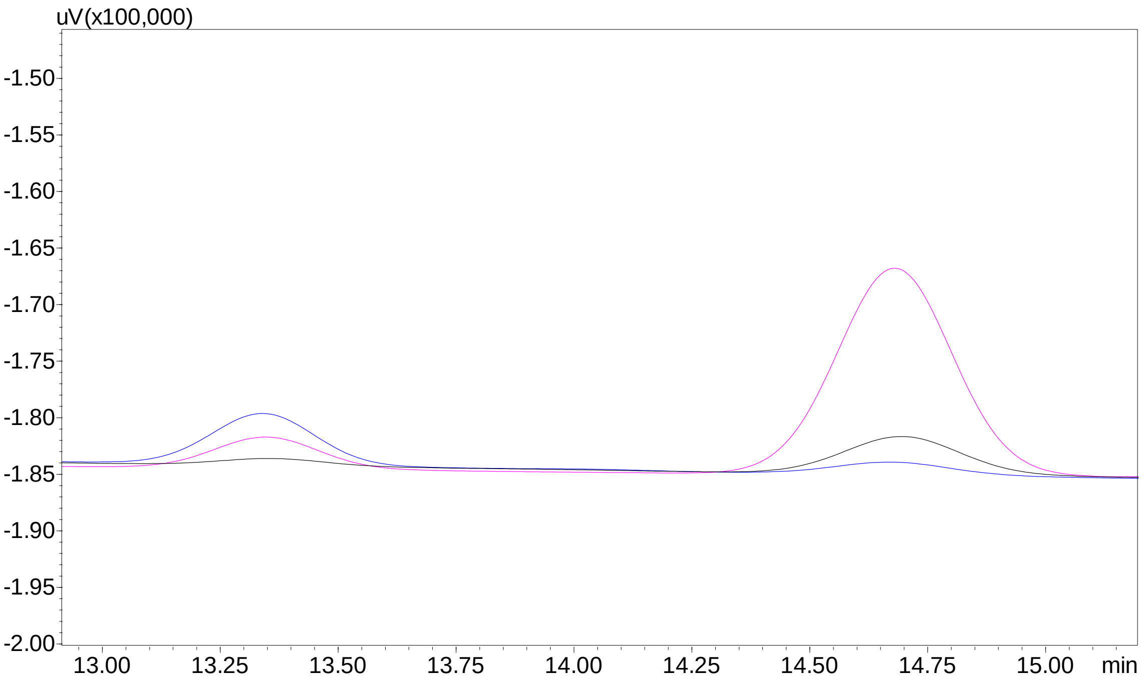

Figure S35: Stacked chiral HPLC chromatograms of reactions of **3ea**, from reactions with different EREDs. **Black**: NCR, **pink**: GluER T36A, **blue**: PETNR.

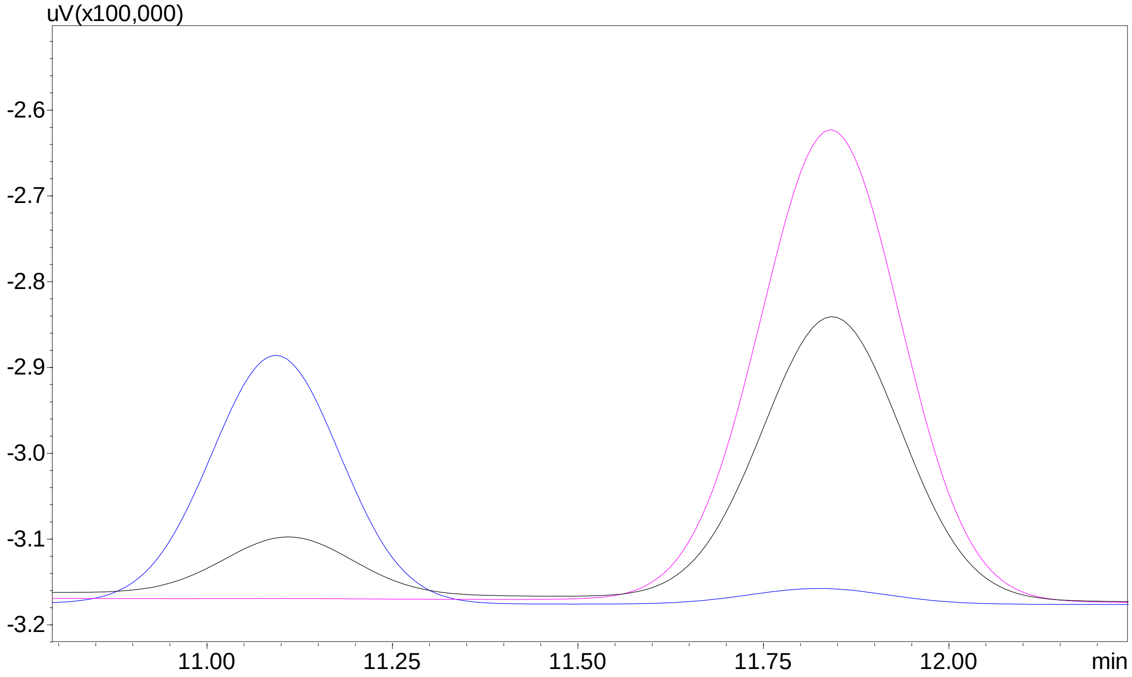

Figure S36: Stacked chiral HPLC chromatograms of reactions of **3fa**, from reactions with different EREDs. **Black**: NCR, **pink**: GluER T36A, **blue**: PETNR.

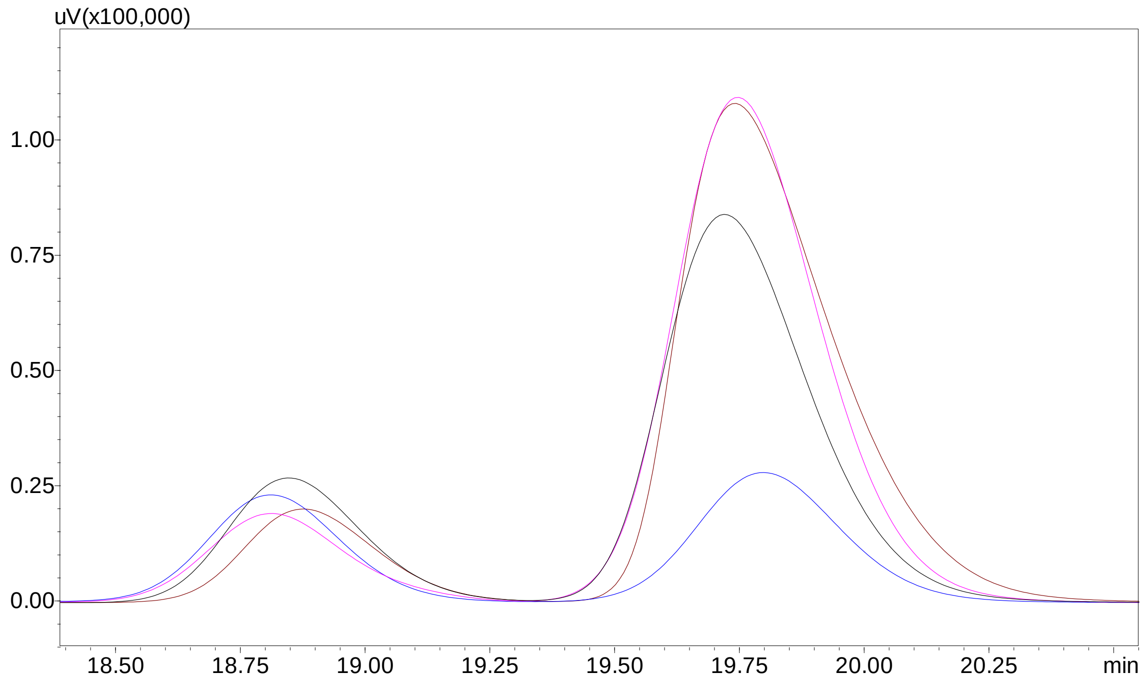

Figure S37: Stacked chiral HPLC chromatograms of reactions of **3ha**, from reactions with different EREDs. **Black**: NCR, **pink**: GluER T36A, **blue**: PETNR, **brown**: purified **3ha** from reaction of GluER T36A.

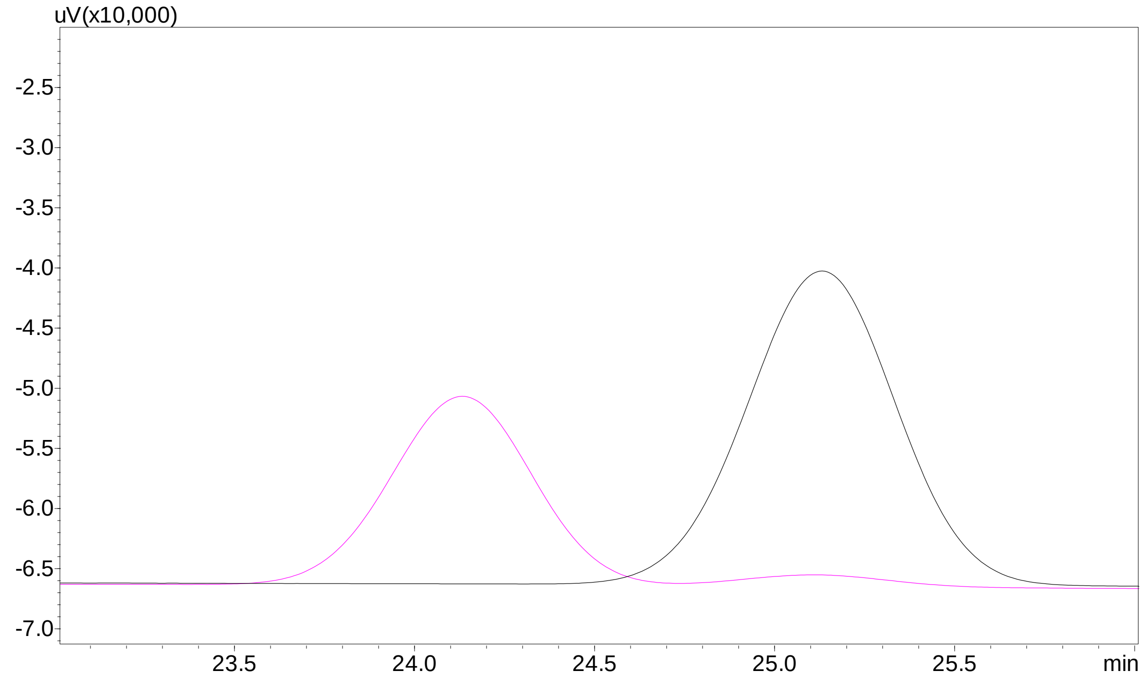

Figure S38: Stacked chiral HPLC chromatograms of reactions of **3ab**, from reactions with different EREDs. **Black**: GluER T36A, **pink**: PETNR.

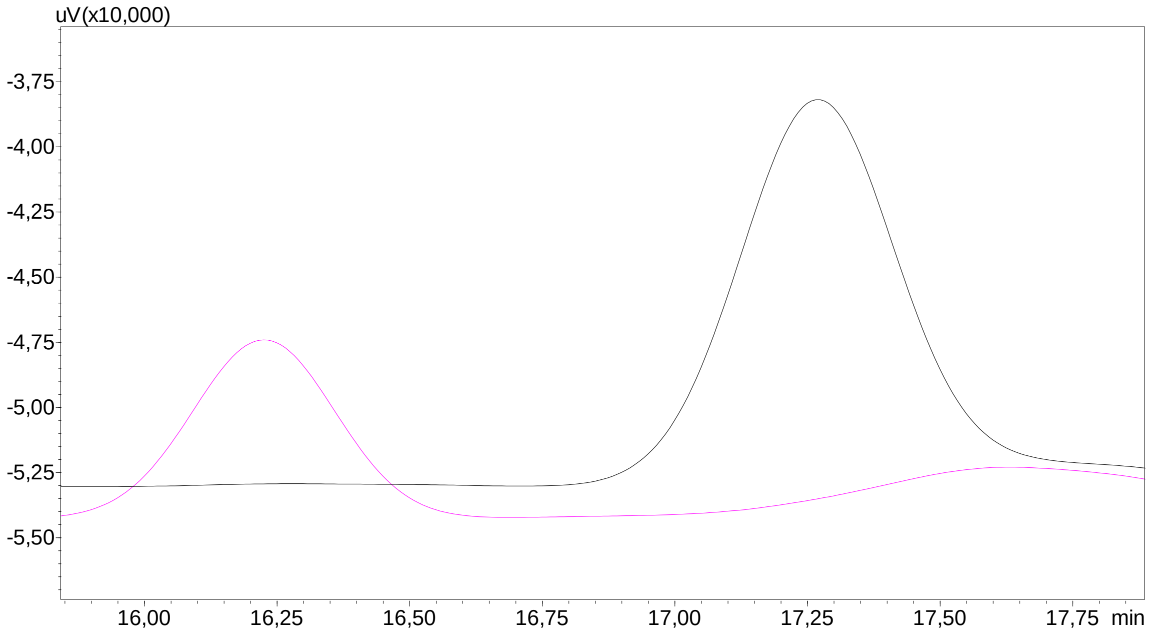

Figure S39: Stacked chiral HPLC chromatograms of reactions of **3ac**, from reactions with different EREDs. **Black**: GluER T36A, **pink**: PETNR.

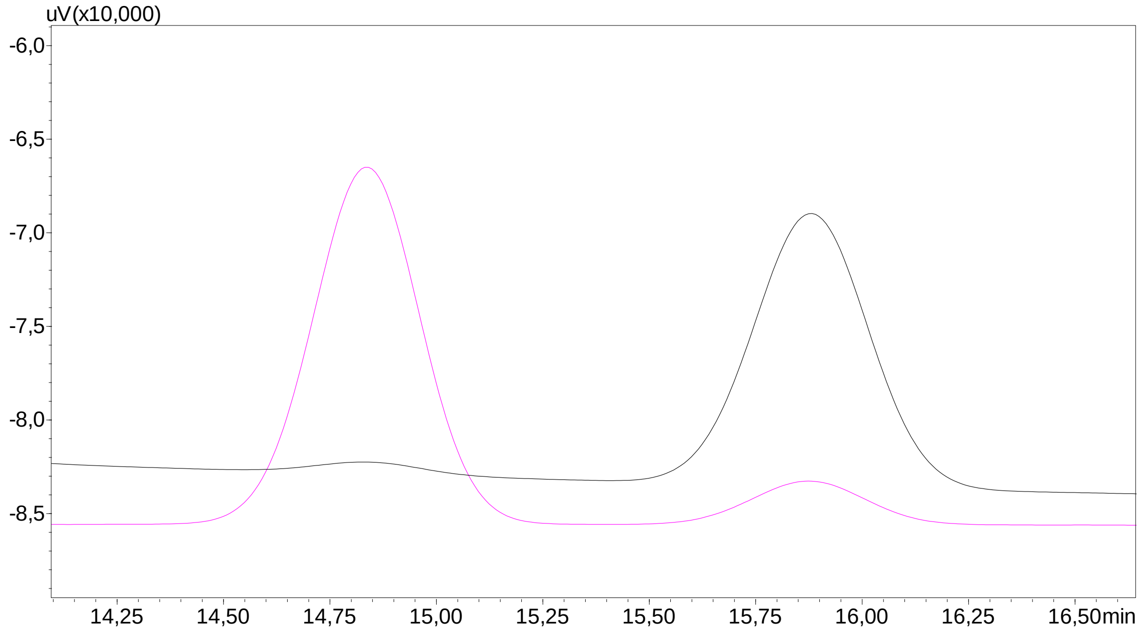

Figure S40: Stacked chiral HPLC chromatograms of reactions of **3ad**, from reactions with different EREDs. **Black**: GluER T36A, **pink**: PETNR.

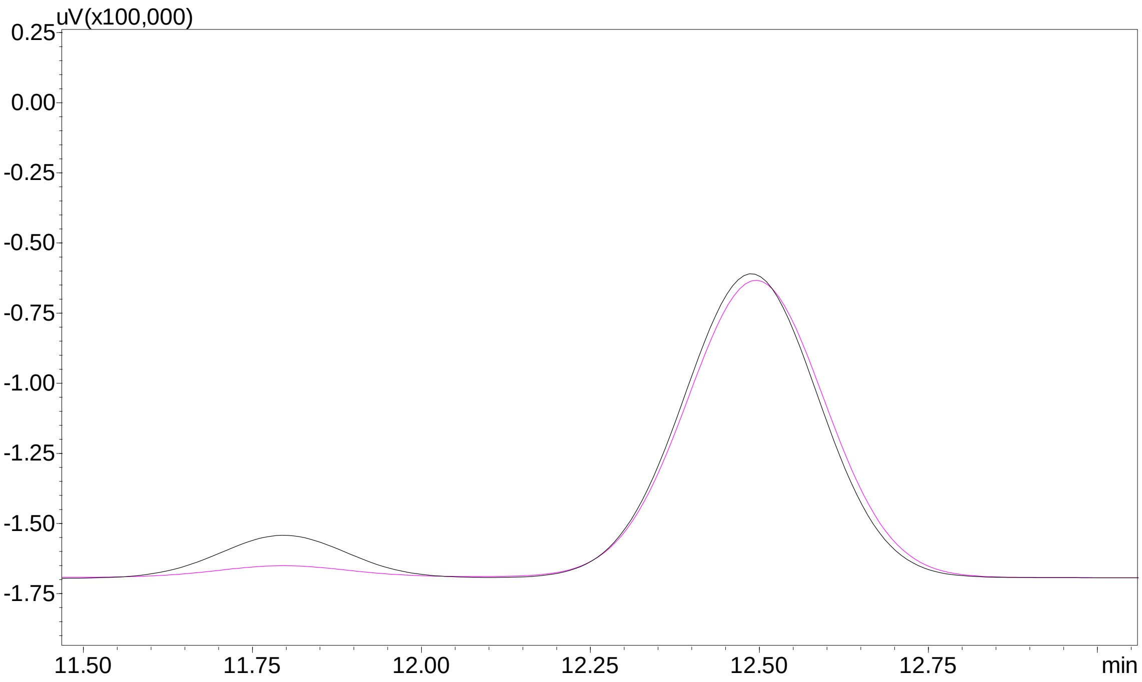

Figure S41: Stacked chiral HPLC chromatograms of isolated **3aa**. **Black**: analytical scale reaction with NCR. **Pink**: preparative scale reaction with GluER T36A.

GC-MS chromatograms

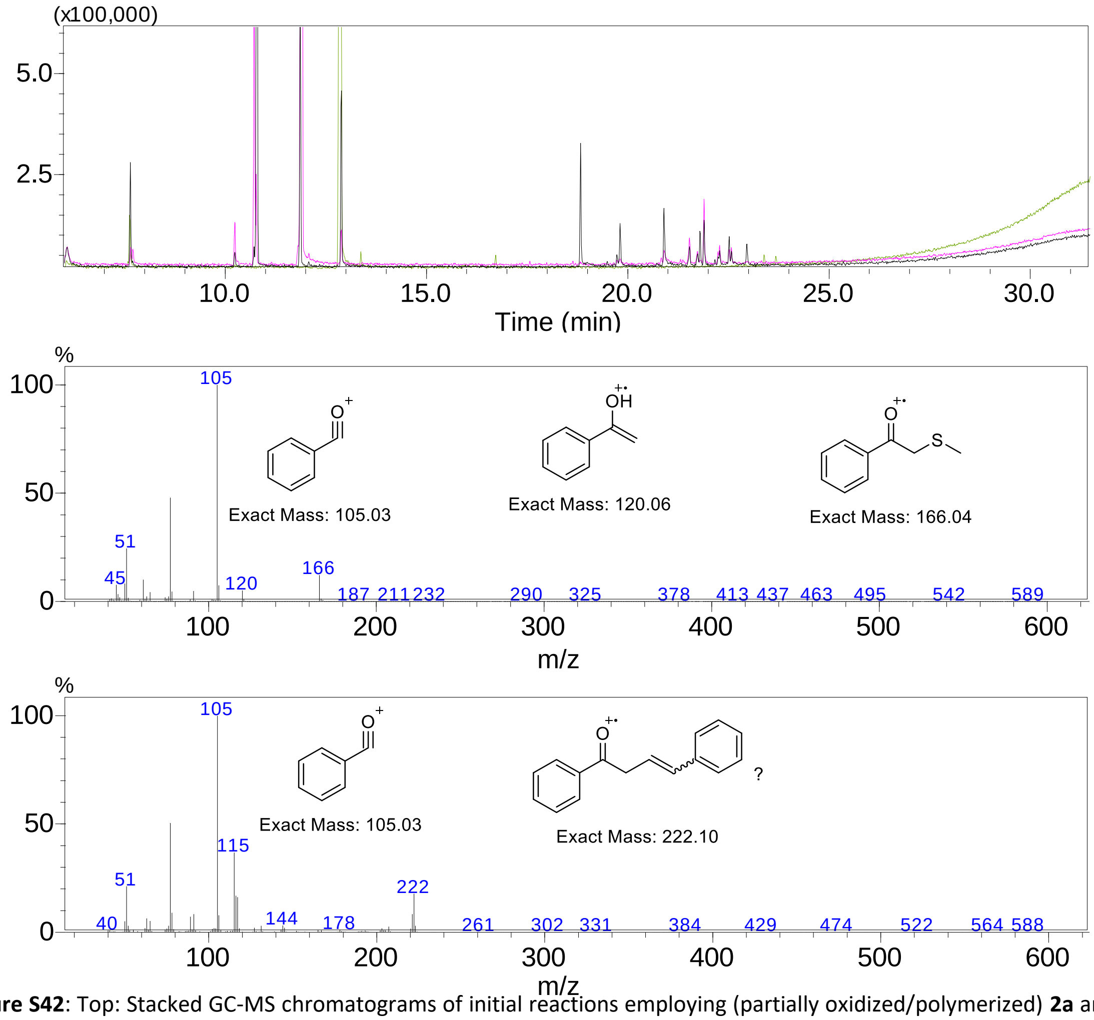

Figure S42: Top: Stacked GC-MS chromatograms of initial reactions employing (partially oxidized/polymerized) **2a** and an excess (3eq.) of **1a**. **Pink**: no ERED, **black**: GluER T36A (NaCl in enzyme preparation); **green**: standard of **6a**. Middle: m/z for peak at 12.9 min in GluER T36A reaction. Bottom: m/z for peak at 18.8 min in GluER T36A reaction.

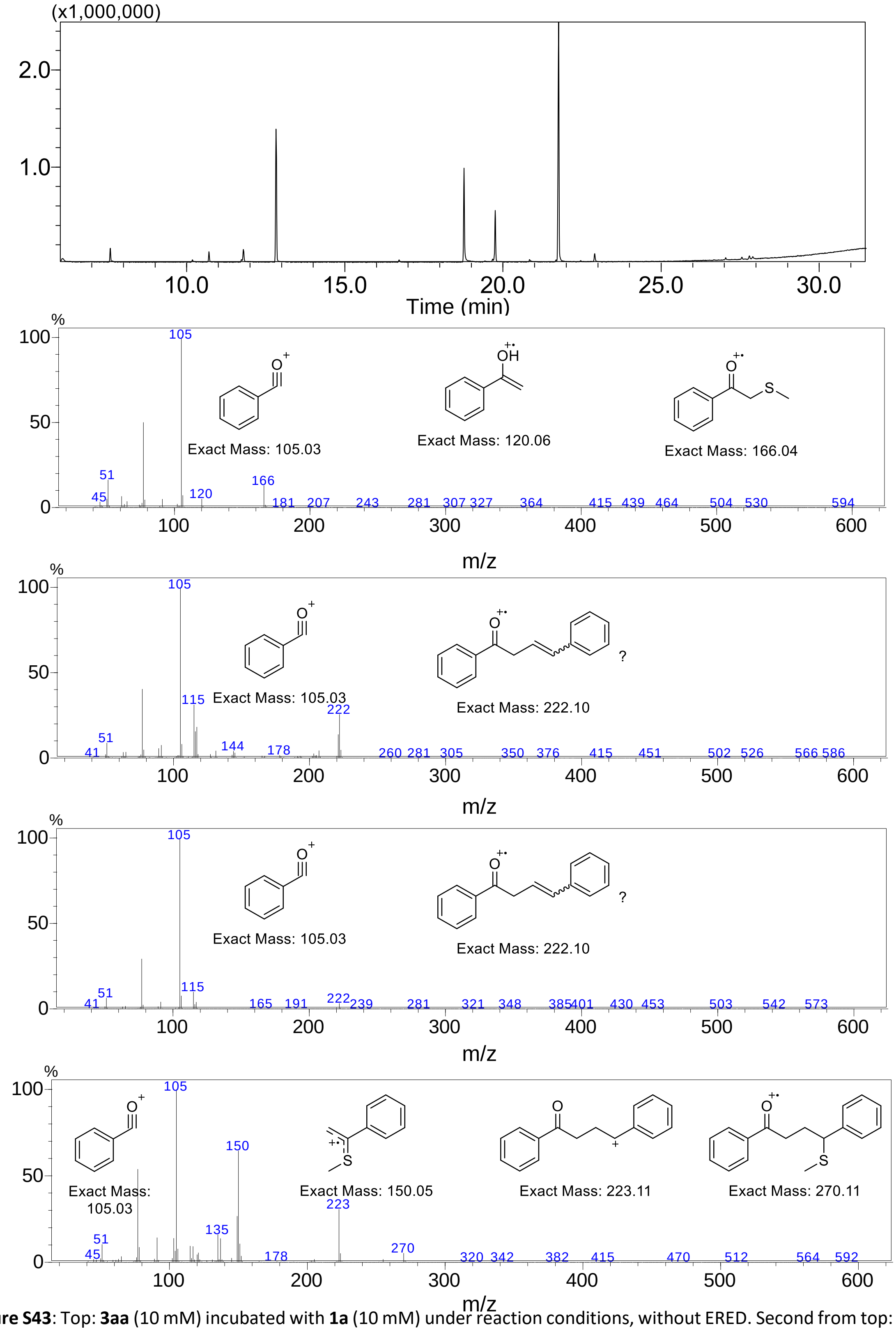

Figure S43: Top: **3aa** (10 mM) incubated with **1a** (10 mM) under reaction conditions, without ERED. Second from top: m/z for peak at 12.9 min. Middle m/z for peak at 18.8 min. Second from bottom: m/z for peak at 19.8 min Bottom: m/z for peak at 21.9 min.

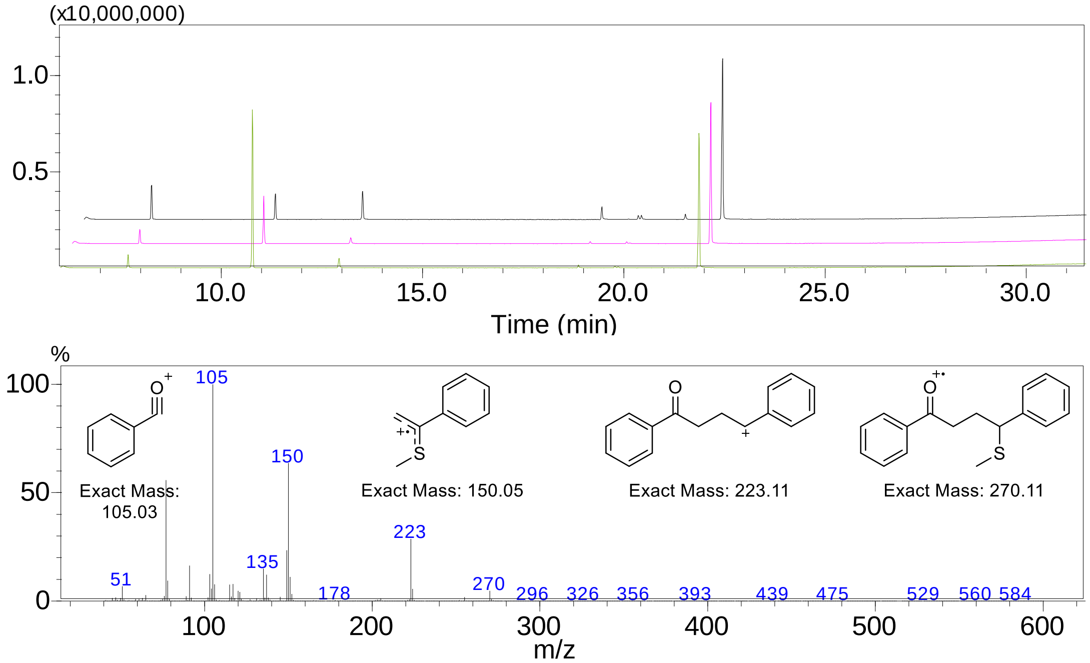

Figure S44: Top: Offset GC-MS chromatograms of reactions employing different amounts of **2a** and **1a**. **Green**: 1.5 eq. **2a**, **pink**: Equal amounts **1a** and **2a**, **black**: 1.5 eq. **1a**. Bottom: m/z for peak at 21.9 min.

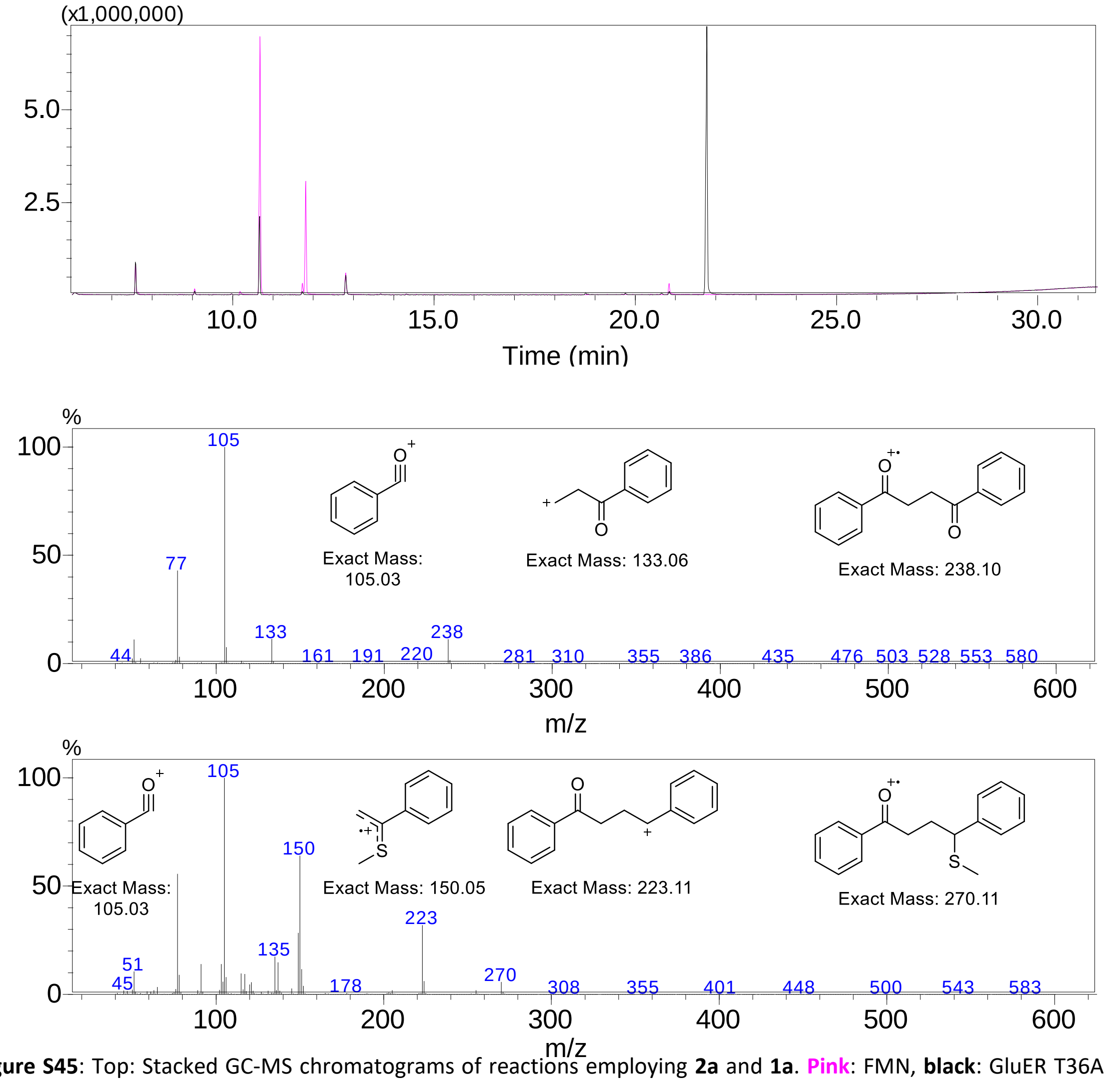

Figure S45: Top: Stacked GC-MS chromatograms of reactions employing **2a** and **1a**. **Pink**: FMN, **black**: GluER T36A **1a**. Middle: Peak at 20.8 min (FMN). Bottom: m/z for peak at 21.8 min (GluER T36A).

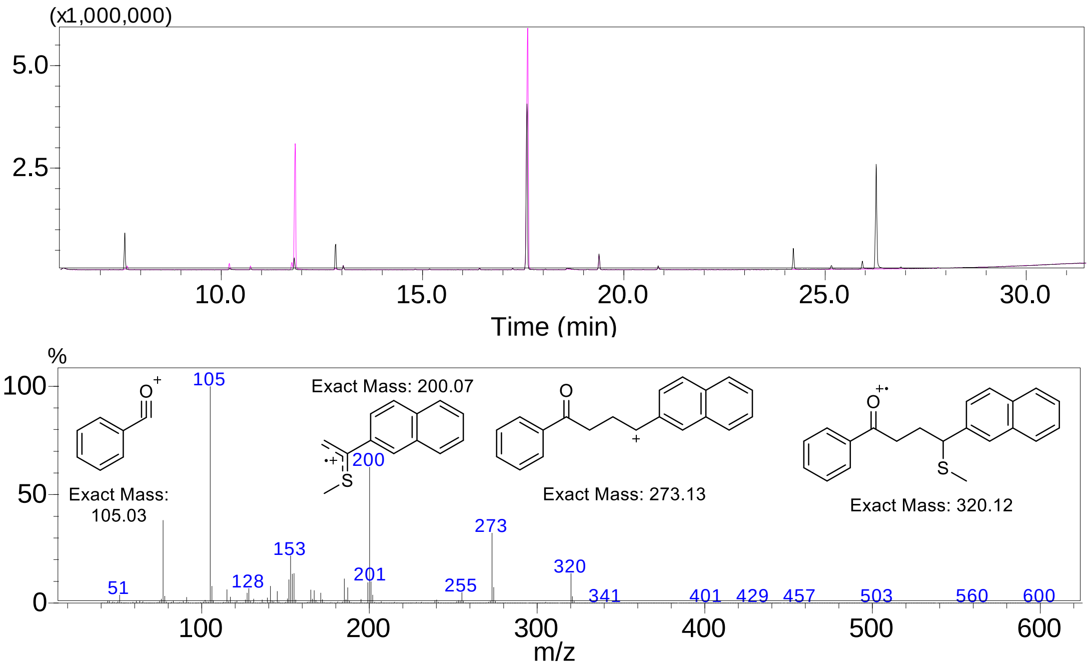

Figure S46: Top: Stacked GC-MS chromatograms of reactions employing **2b** and **1a**. **Pink**: no ERED, **black**: GluER T36A **1a**. Bottom: m/z for peak at 26.3 min.

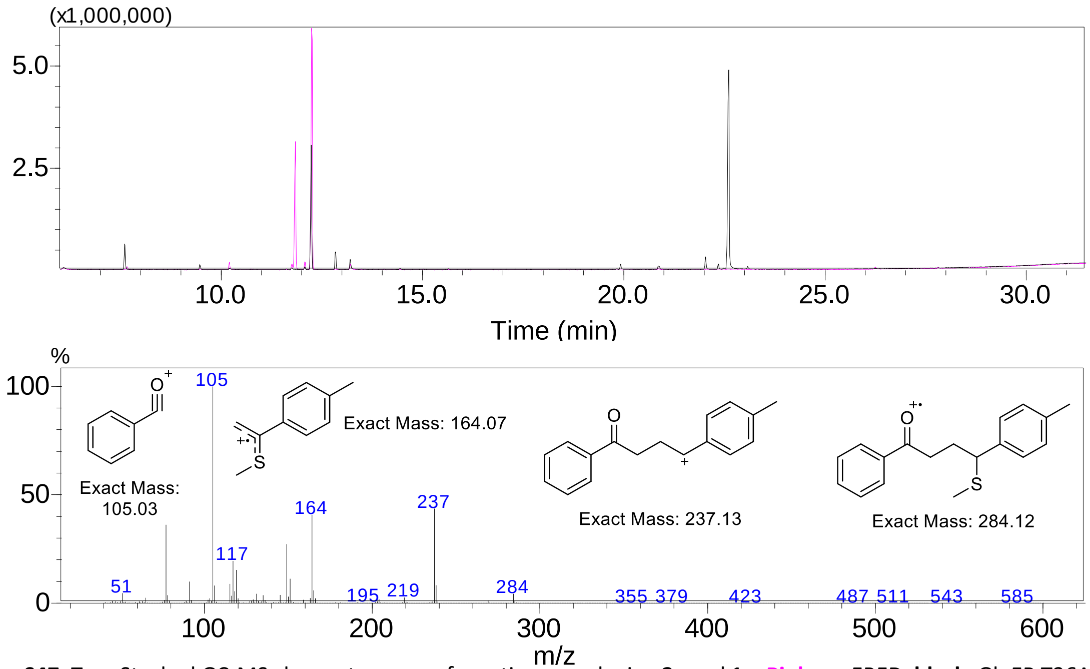

Figure S47: Top: Stacked GC-MS chromatograms of reactions employing **2c** and **1a**. **Pink**: no ERED, **black**: GluER T36A **1a**. Bottom: m/z for peak at 22.6 min.

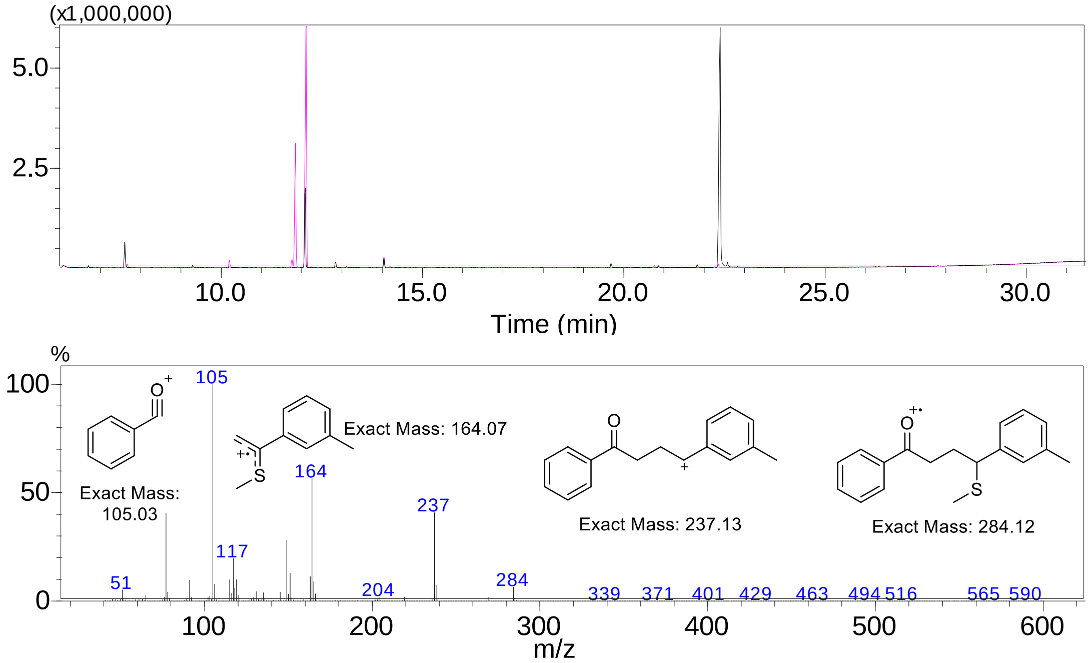

Figure S48: Top: Stacked GC-MS chromatograms of reactions employing **2d** and **1a**. **Pink**: no ERED, **black**: GluER T36A **1a**. Bottom: m/z for peak at 22.4 min.

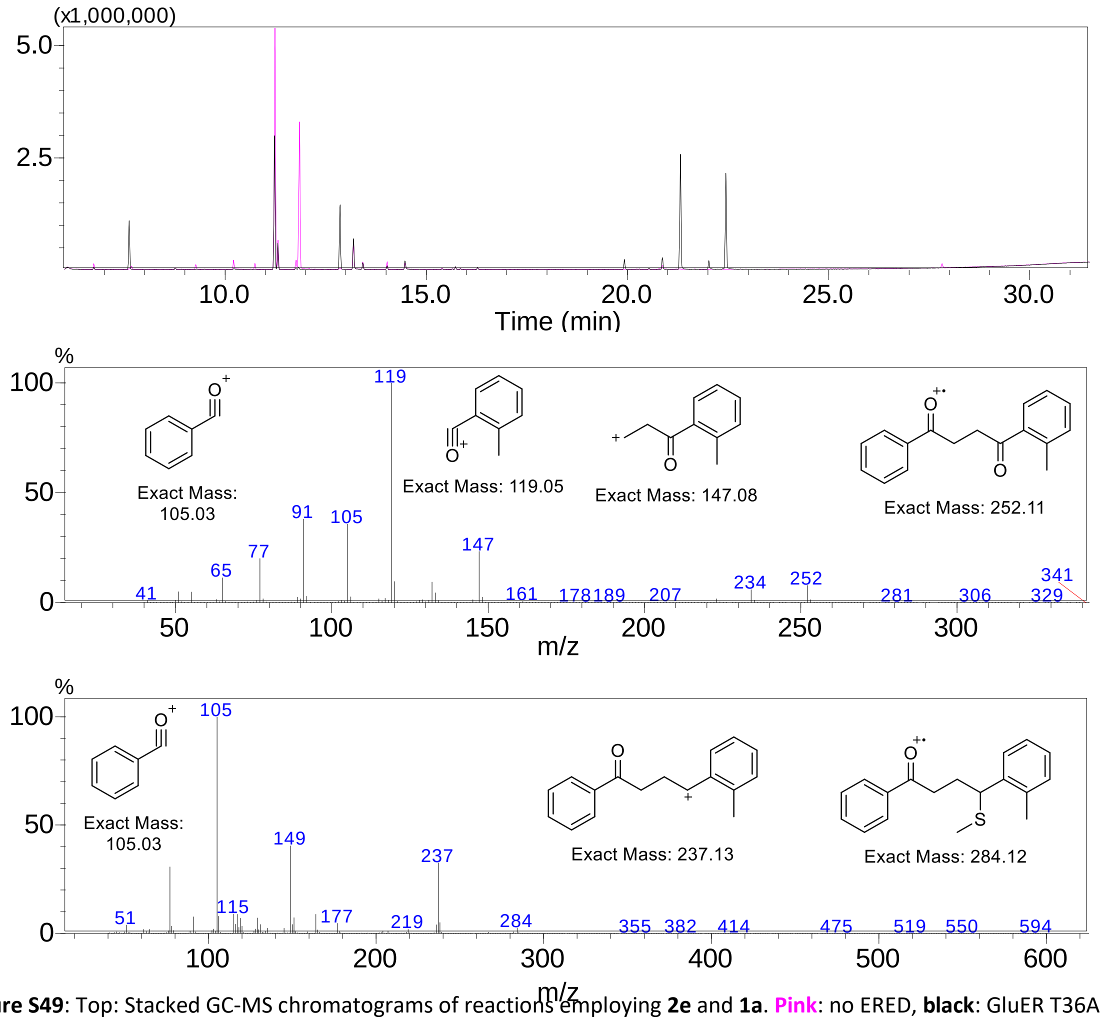

Figure S49: Top: Stacked GC-MS chromatograms of reactions employing **2e** and **1a**. **Pink**: no ERED, **black**: GluER T36A **1a**. Middle: m/z for peak at 21.3 min Bottom: m/z for peak at 22.4 min.

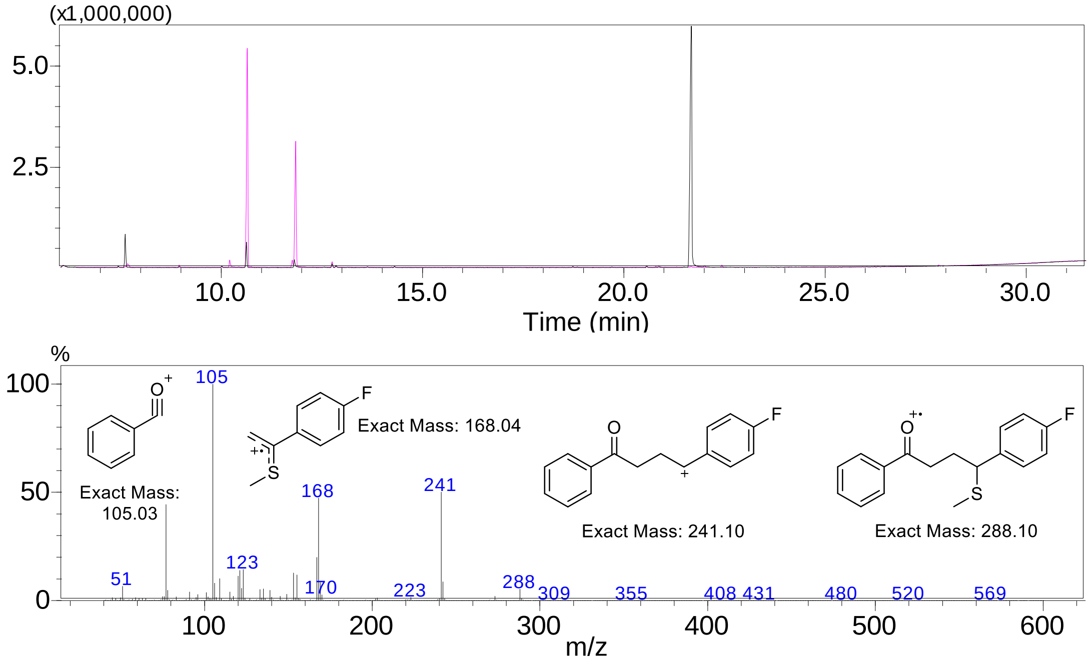

Figure S50: Top: Stacked GC-MS chromatograms of reactions employing **2f** and **1a**. **Pink**: no ERED, **black**: GluER T36A **1a**. Bottom: m/z for peak at 21.7 min.

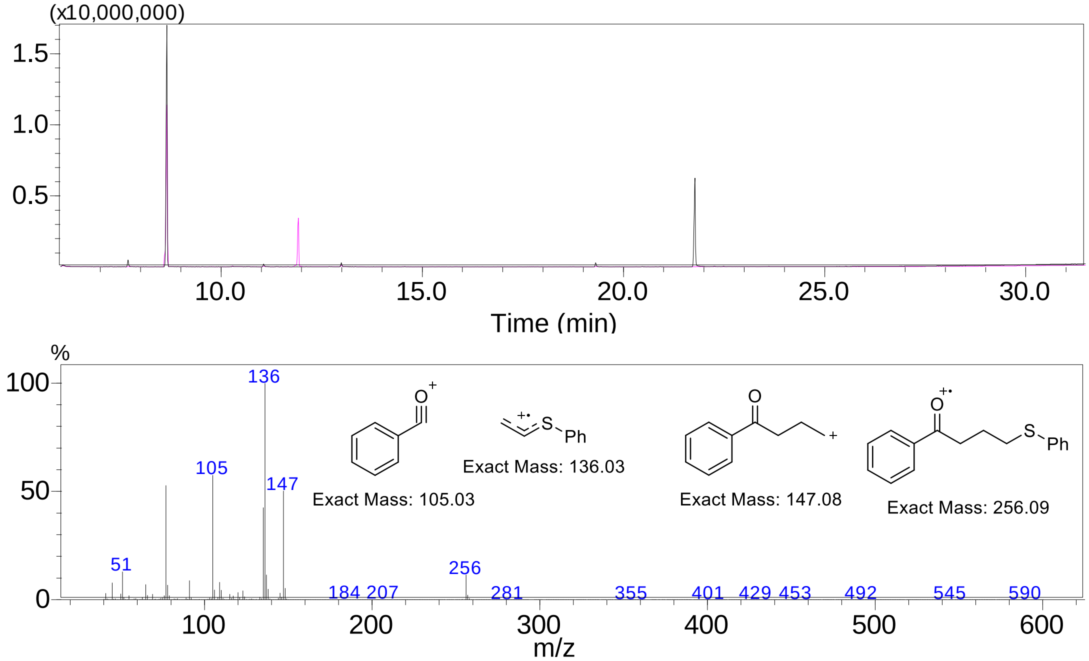

Figure S51: Top: Stacked GC-MS chromatograms of reactions employing **2g** (3 eq.) and **1a**. **Pink**: no ERED, **black**: GluER T36A **1a**. Bottom: m/z for peak at 21.8 min.

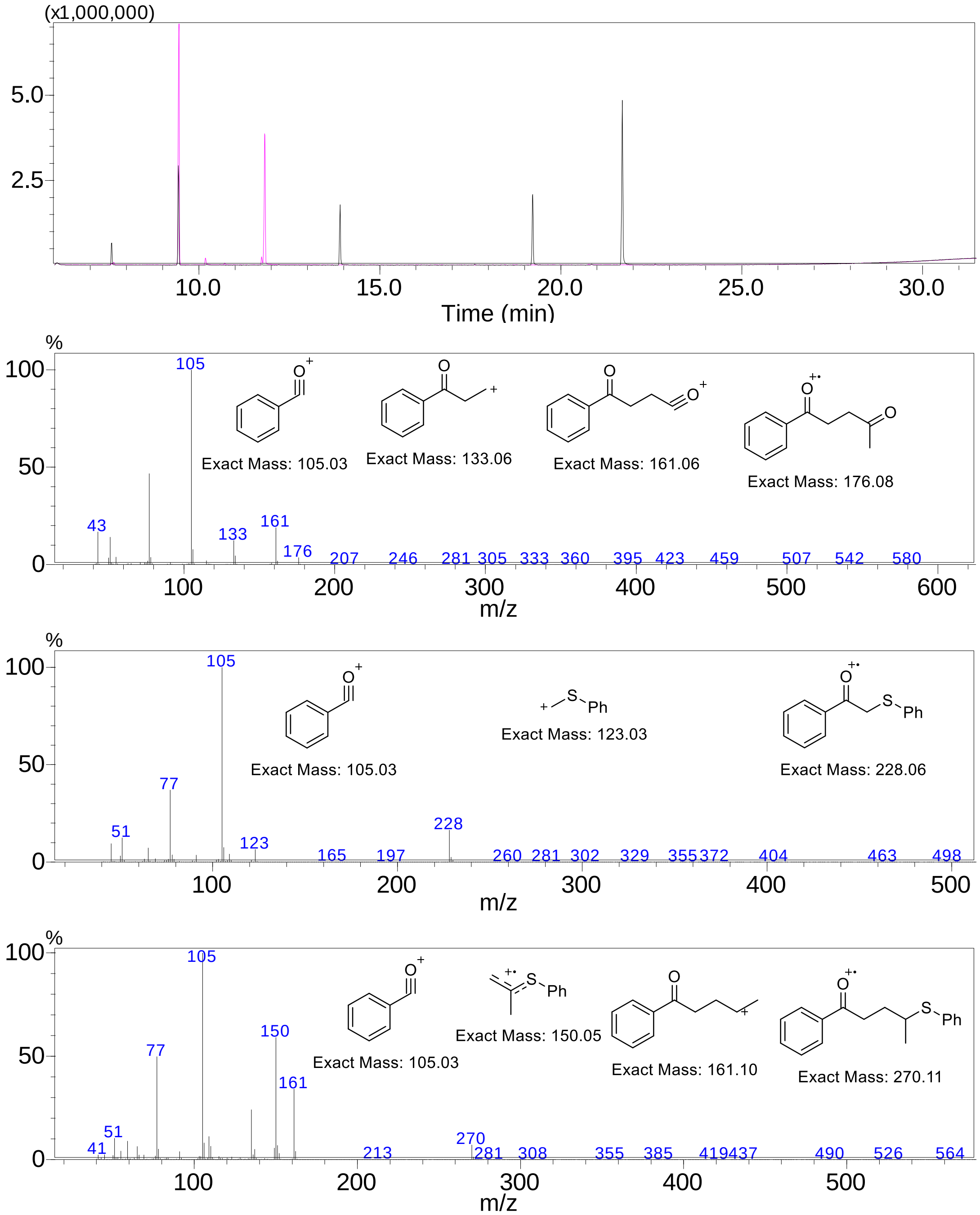

Figure S52: Top: Stacked GC-MS chromatograms of reactions employing **2h** and **1a**. Pink: no ERED, **black**: GluER T36A **1a**. Second from top: m/z for peak at 13.9 min. Second from Bottom: m/z for peak at 19.2 min. Bottom: m/z for peak at 21.7 min.

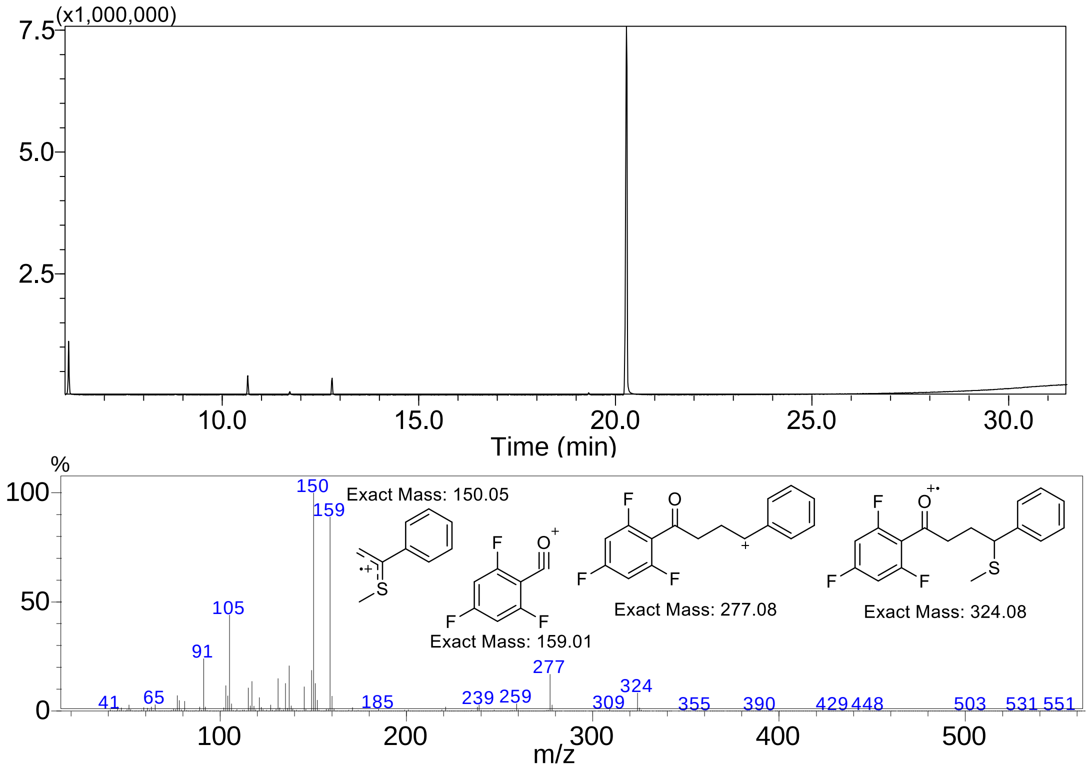

Figure S53: Top: GC-MS chromatograms of reactions employing **2a** and **1b** with GluER T36A **1a**. Bottom: m/z for peak at 20.3 min.

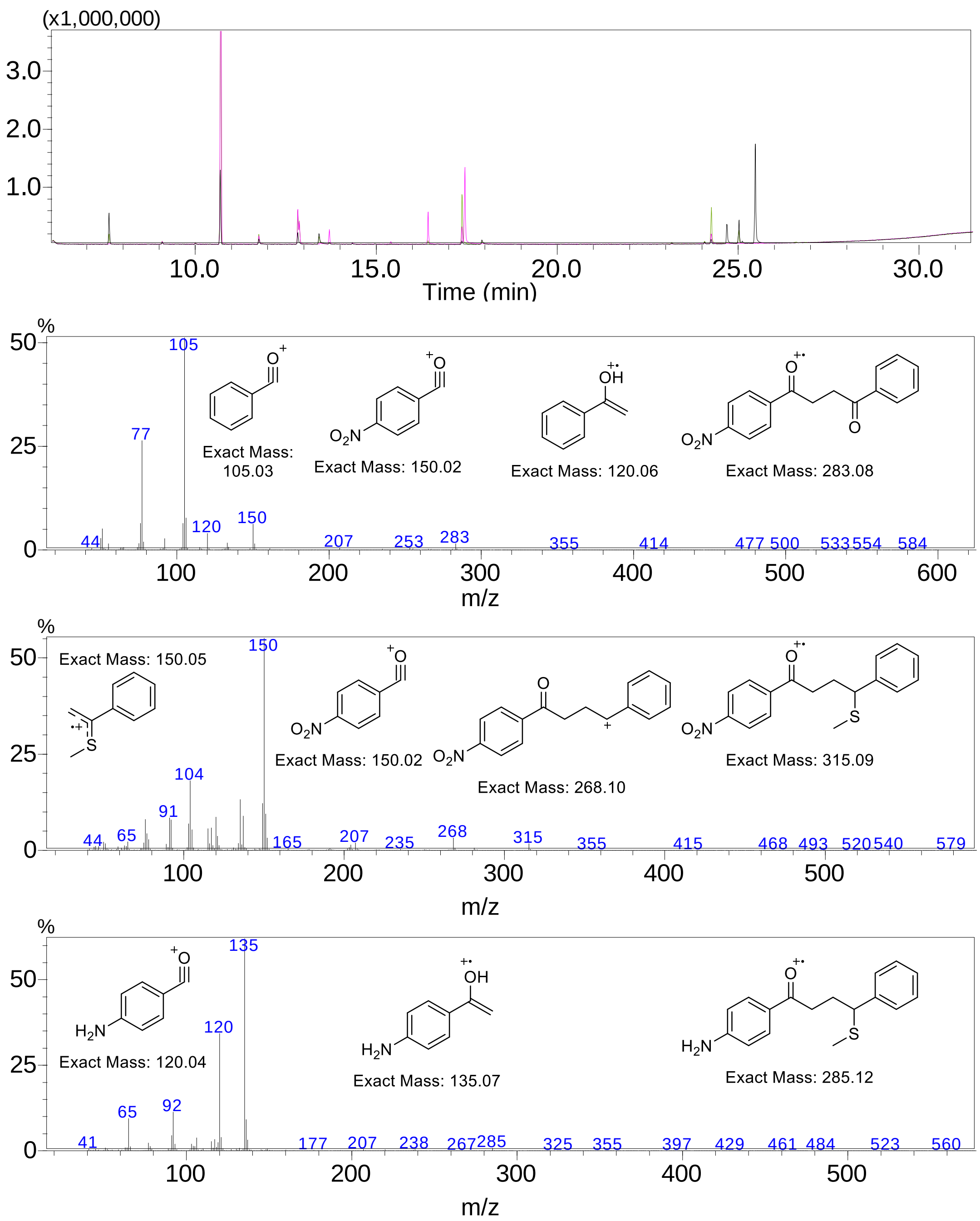

Figure S54: Top: Stacked GC-MS chromatograms of reactions employing **2a** and **1c**. Pink: no ERED, **black**: GluER T36A. **1a**. Second from top: m/z for peak at 24.3 min (PETNR). Second from bottom: m/z for peak at 25.0 min (GluER T36A). Bottom: m/z for peak at 25.5 min (GluER T36A).

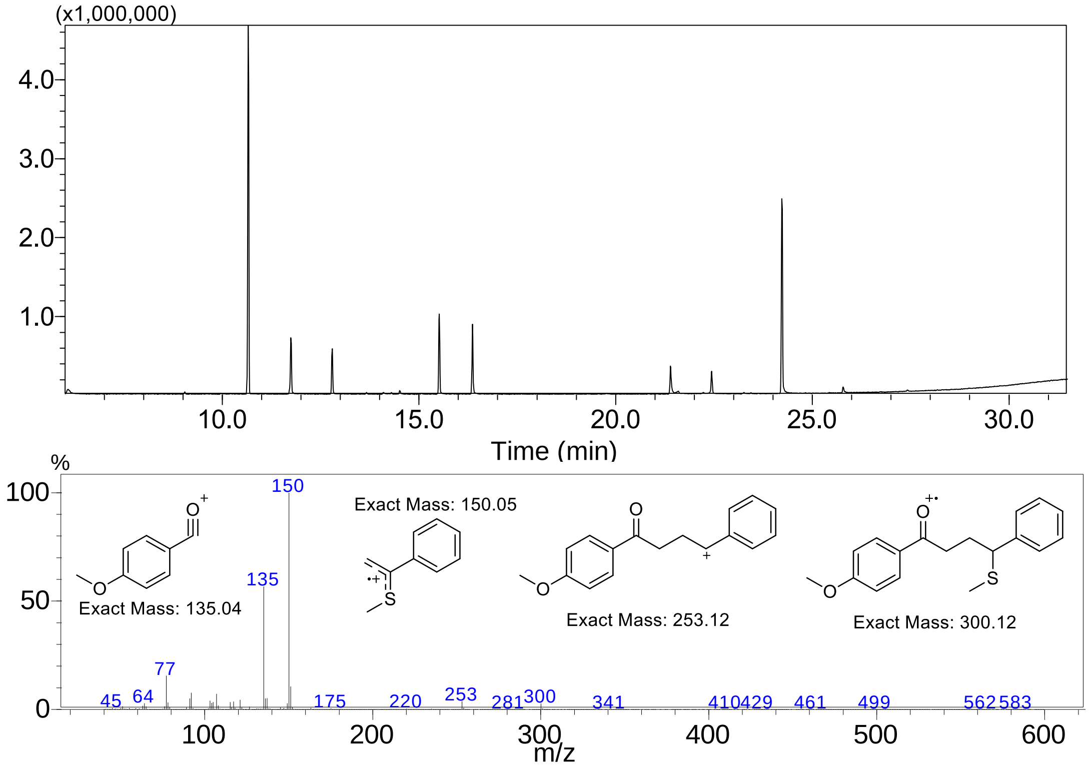

Figure S55: Top: GC-MS chromatograms of reactions employing **2a** and **1d** with GluER T36A **1a**. Bottom: m/z for peak at 20.3 min.

NMR spectra

*^{1}H-NMR of **2a***

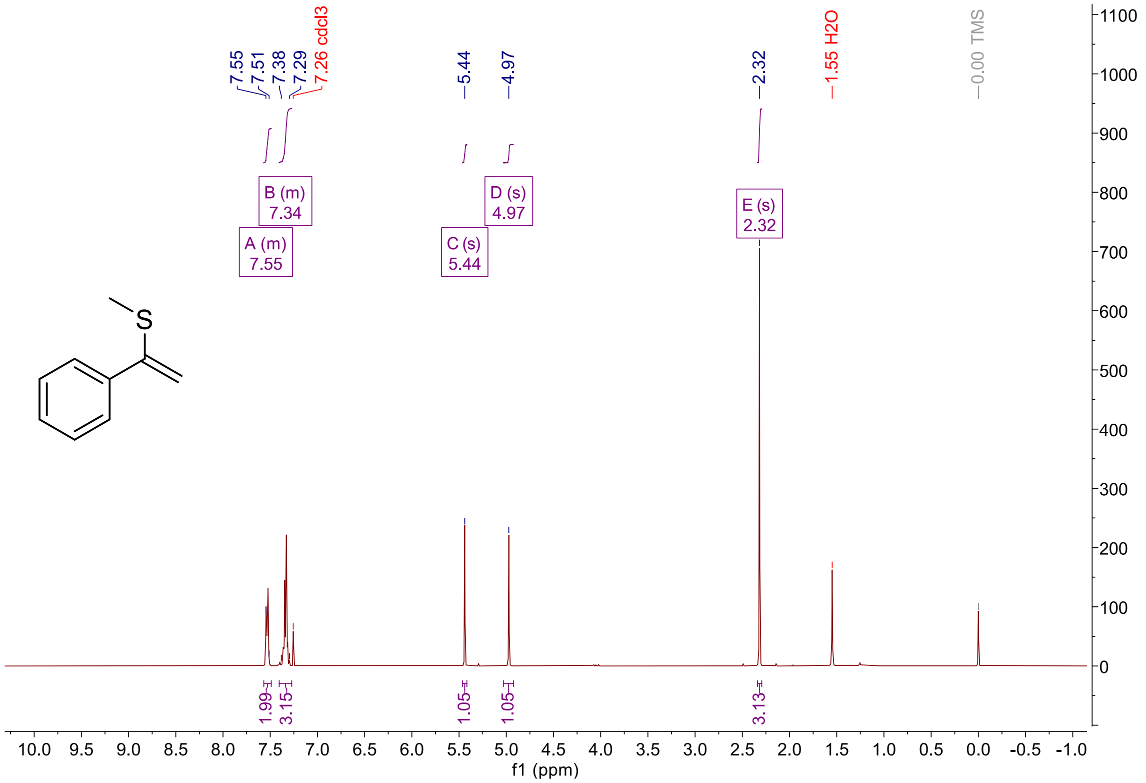

*^{13}C{^{1}H}-NMR of **2a***

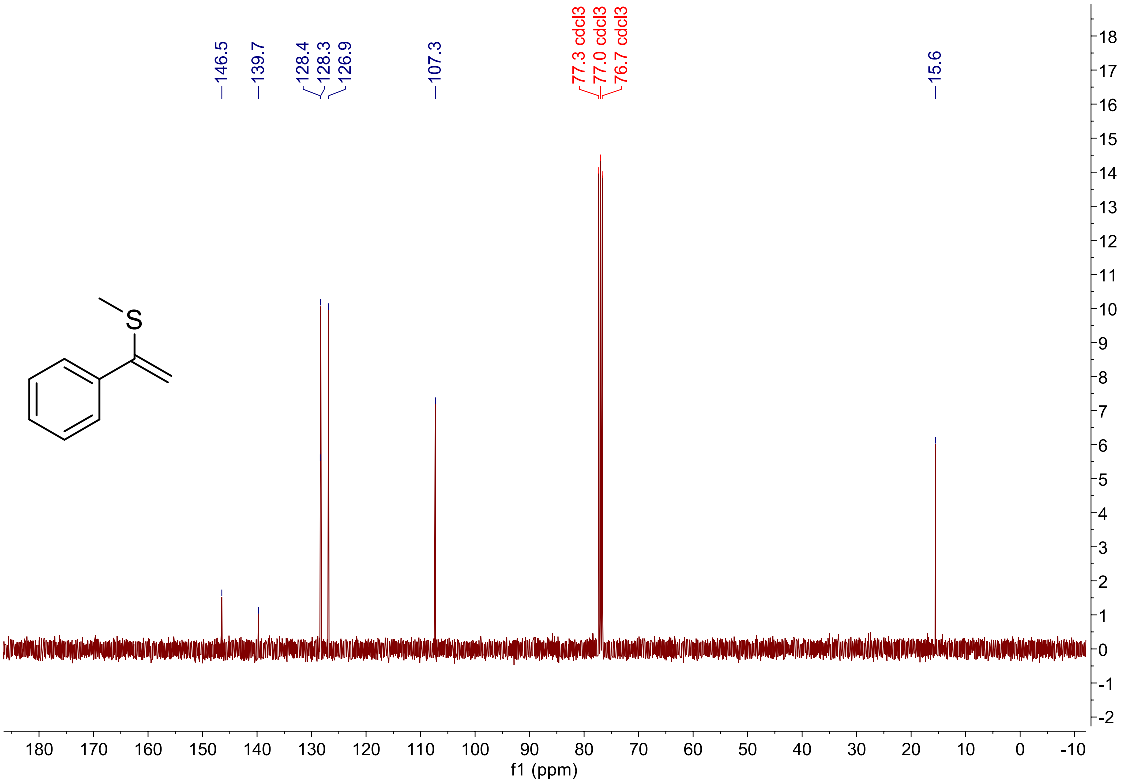

*Multiplicity-edited ^{1}H-^{13}C-HSQC NMR of **2a***

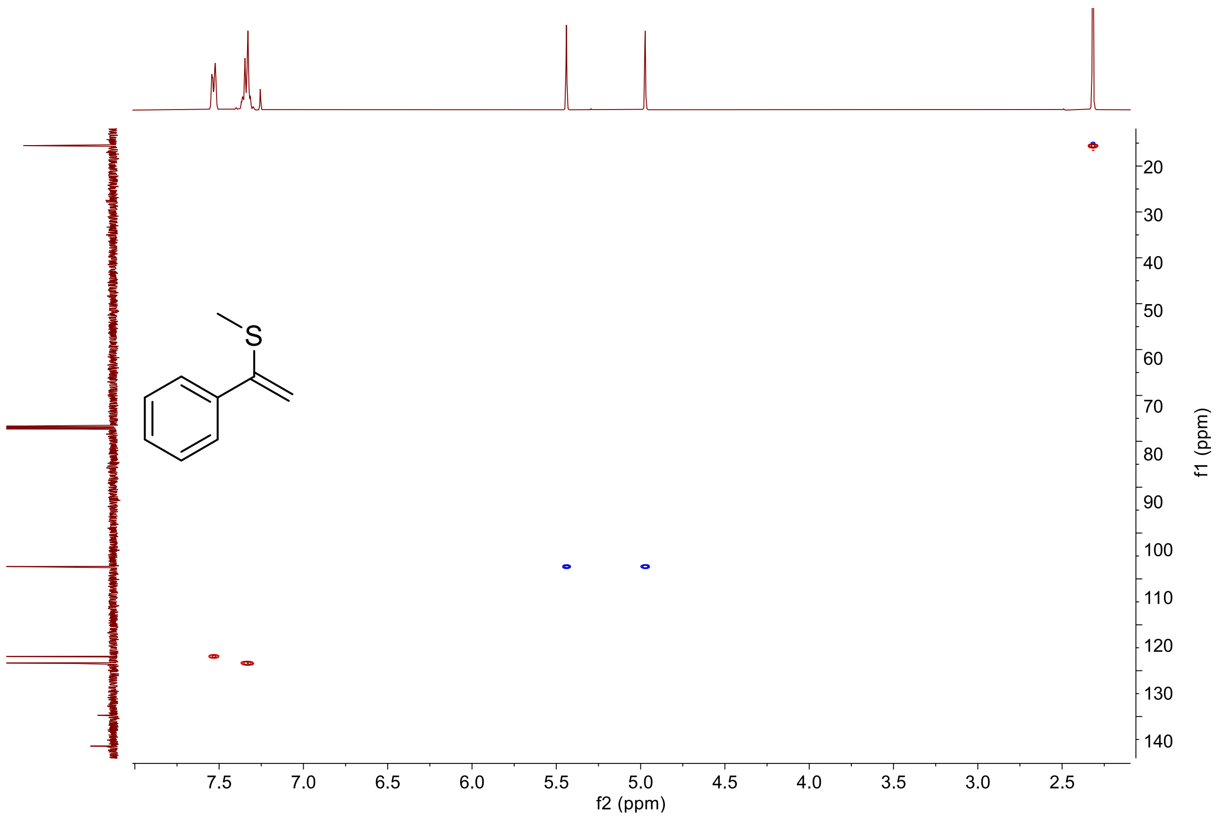

*^{1}H-NMR of **2b***

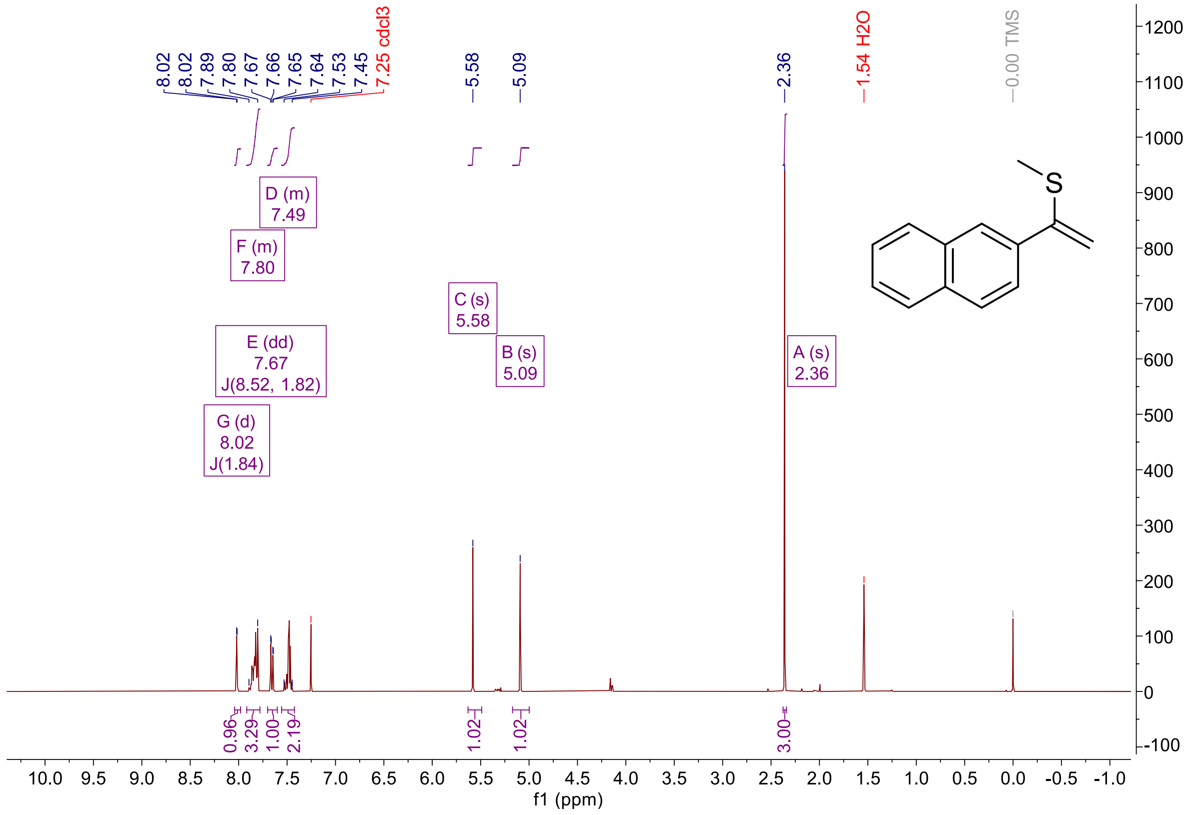

$^{13}C\{^1H\}$-NMR of **2b**

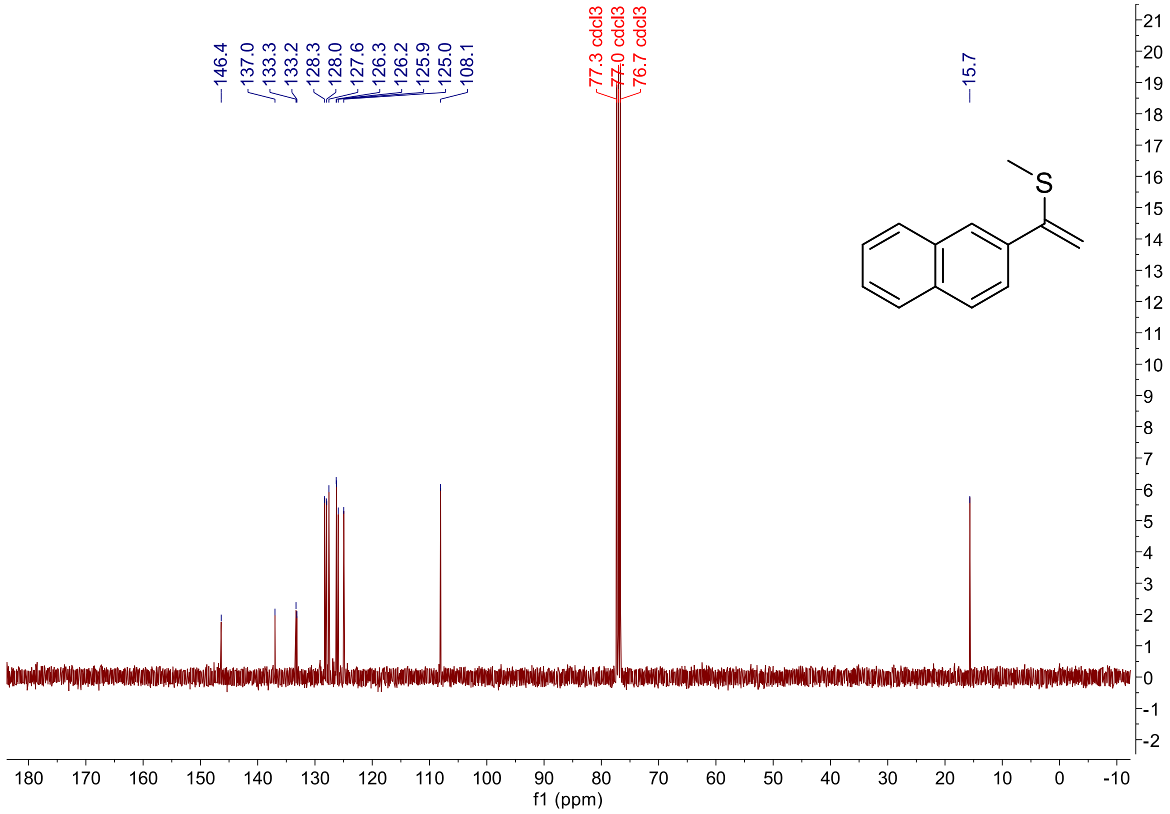

Multiplicity-edited ^1H-^{13}C-HSQC NMR of **2b**

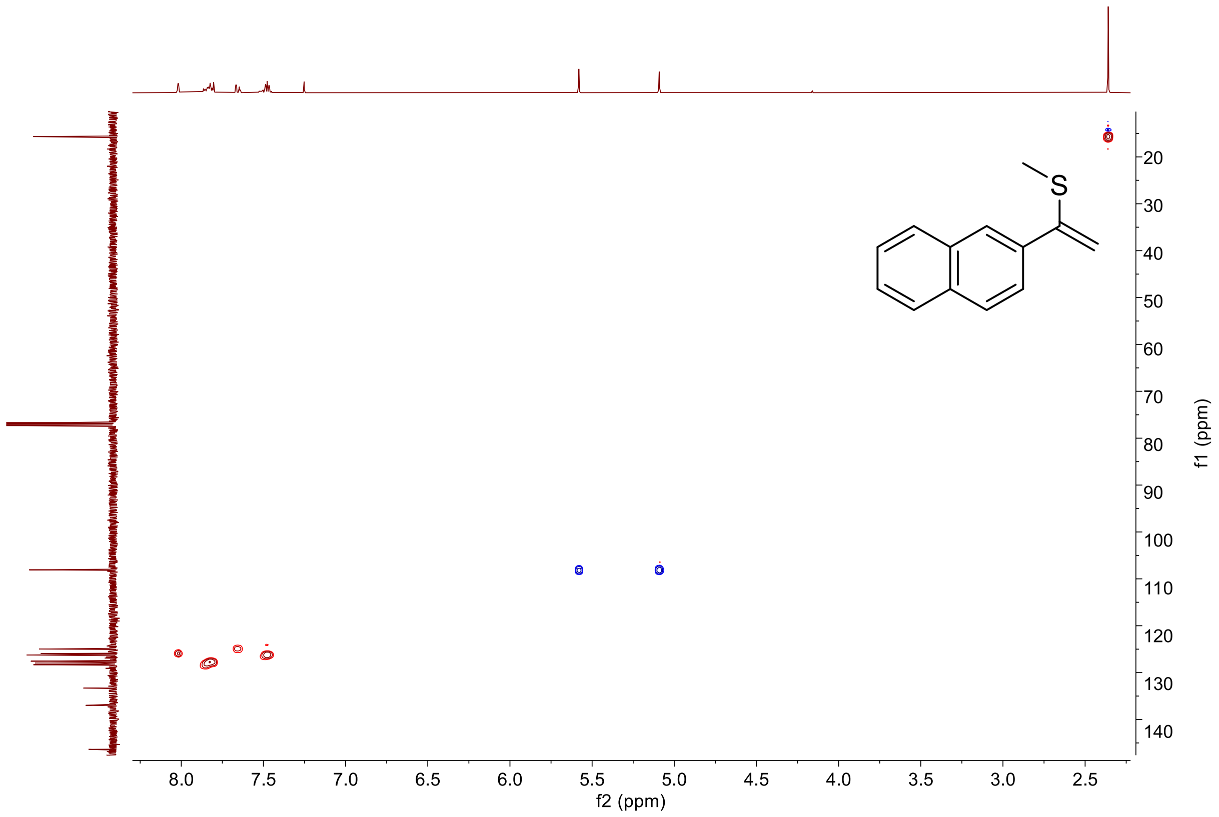

^{1}*H-NMR of **2c***

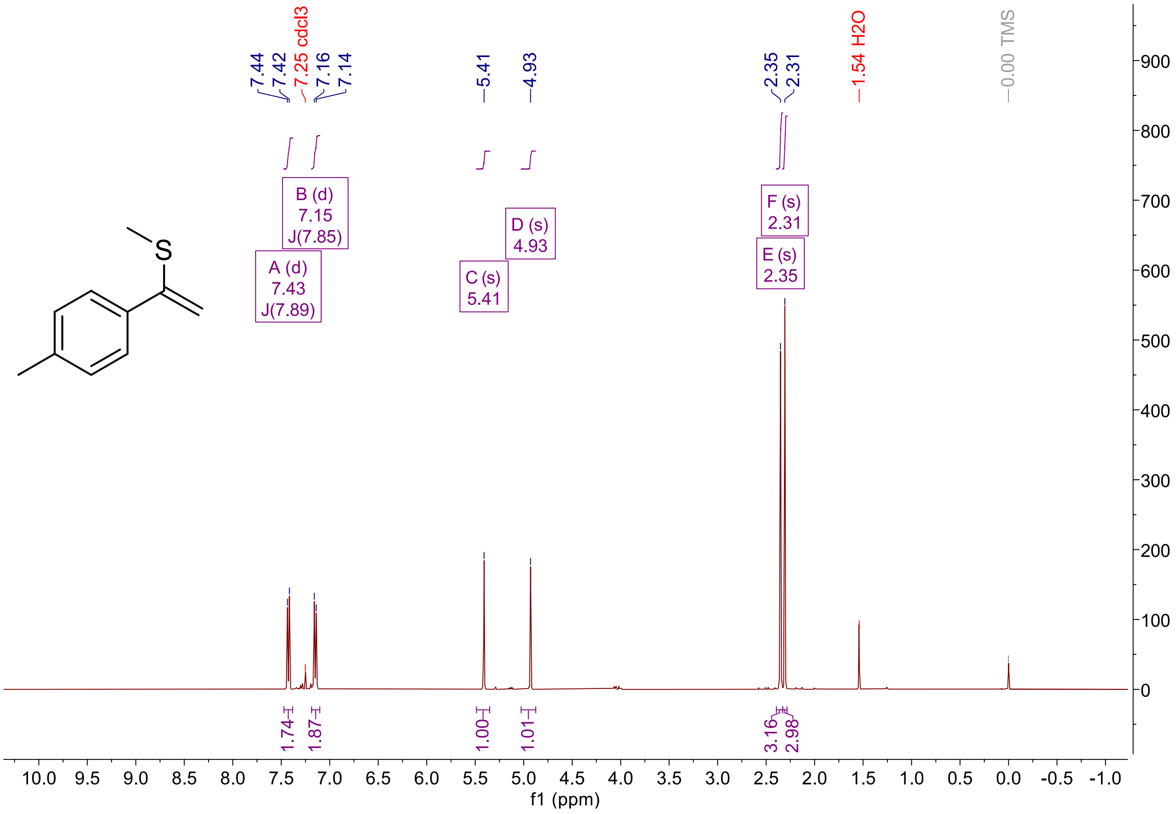

^{13}*C{^{1}H}-NMR of **2c***

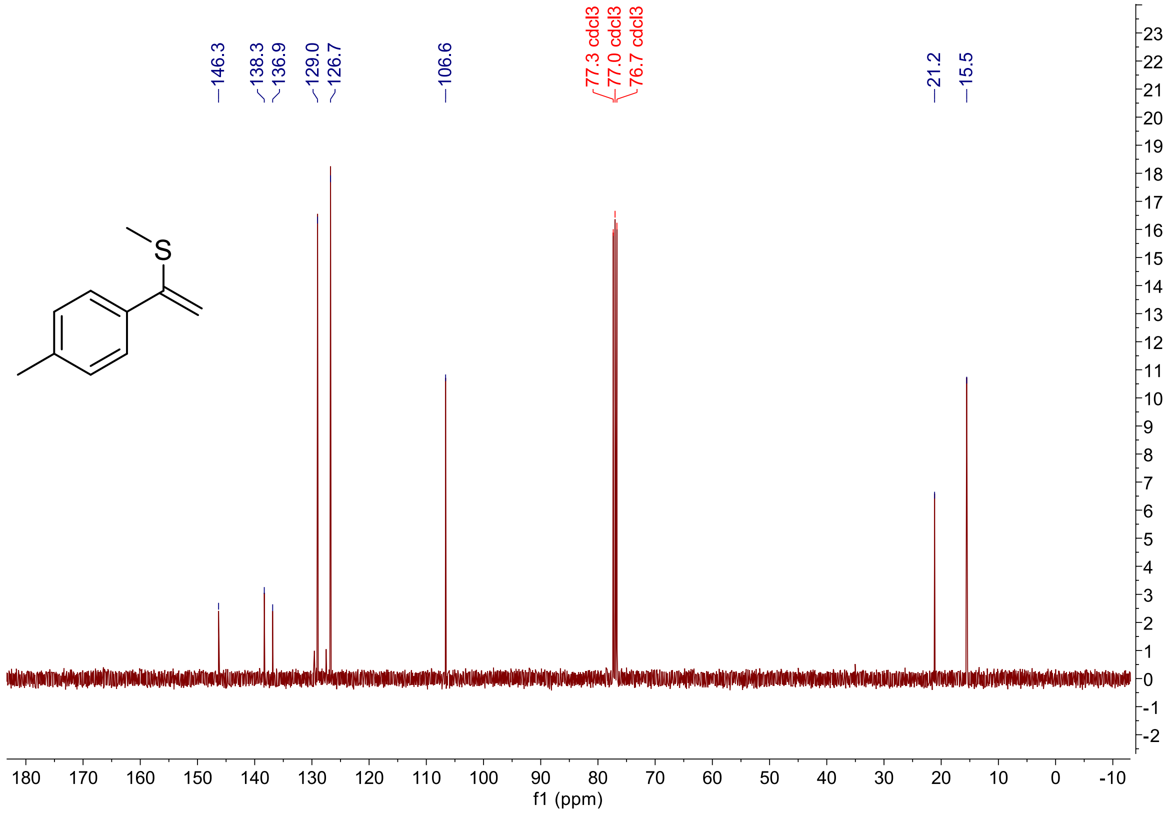

Multiplicity-edited ^{1}H-^{13}C*-HSQC NMR of* ***2c***

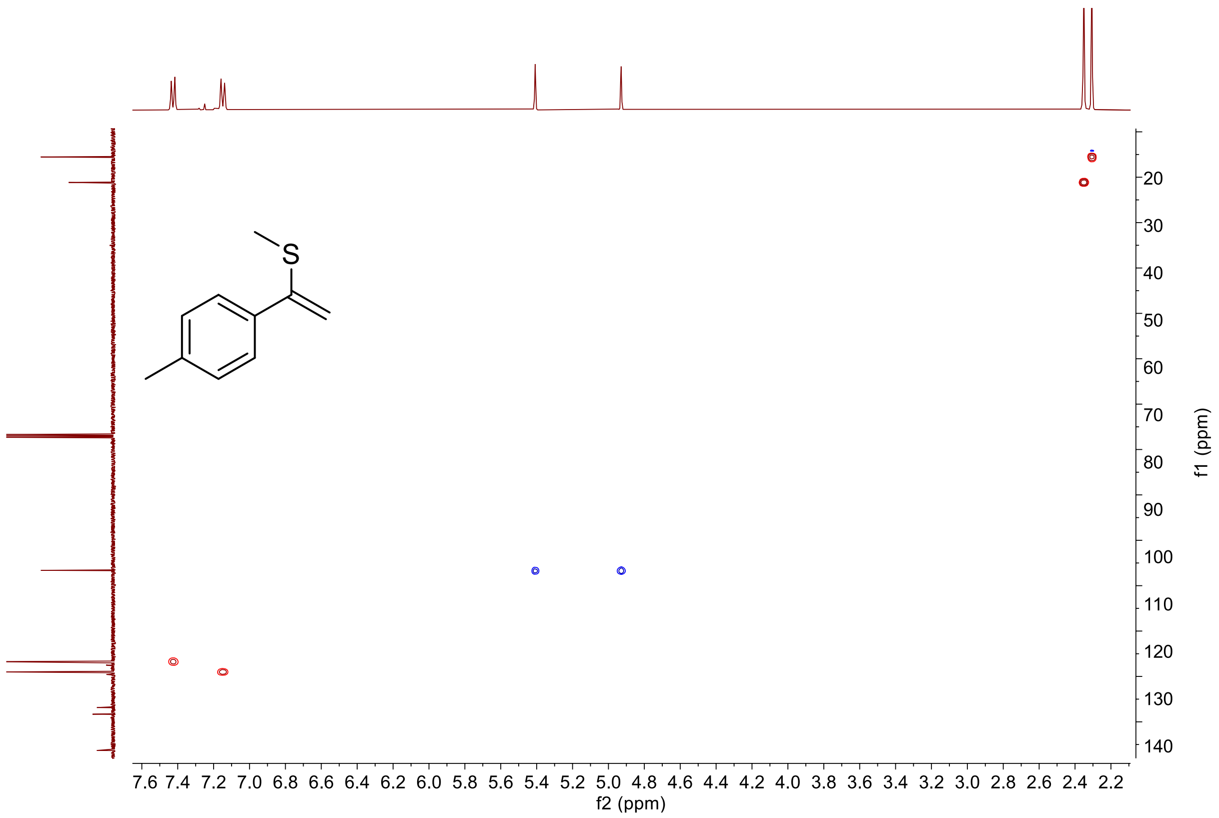

^{1}H*-NMR of* ***2d***

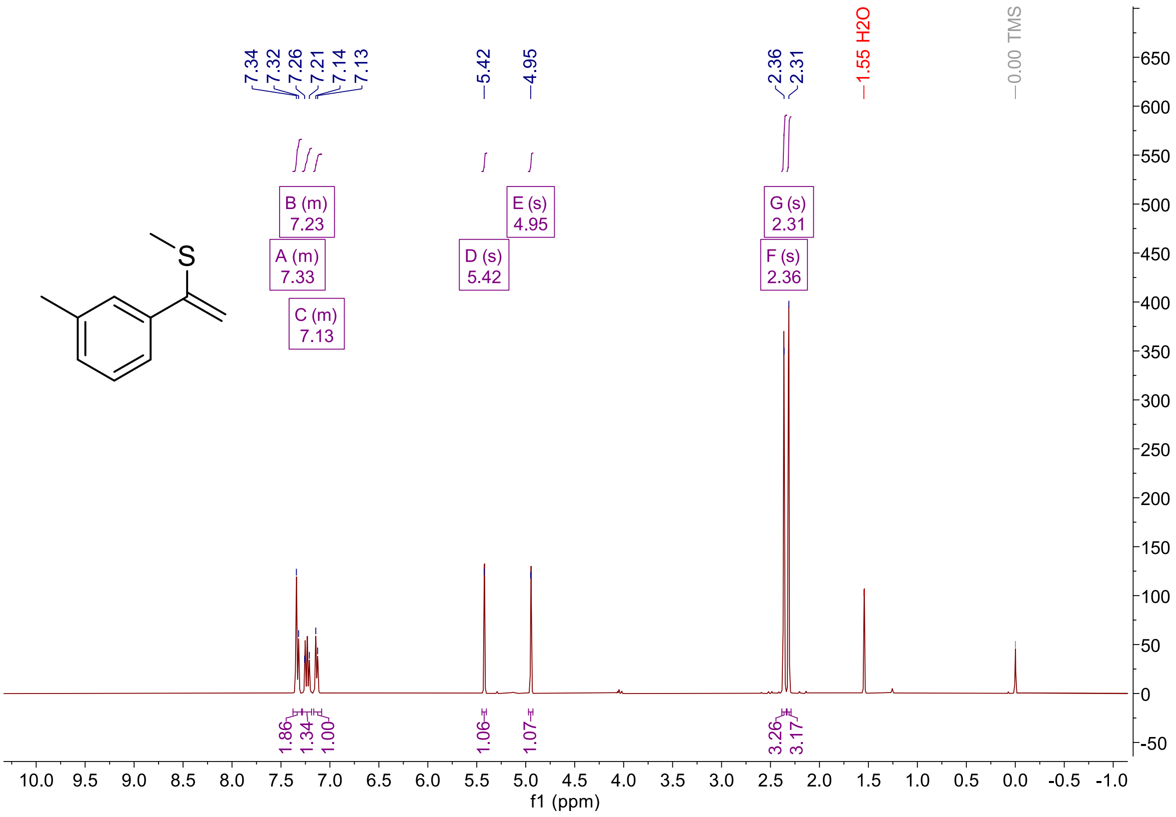

*$^{13}C\{^{1}H\}$-NMR of **2d***

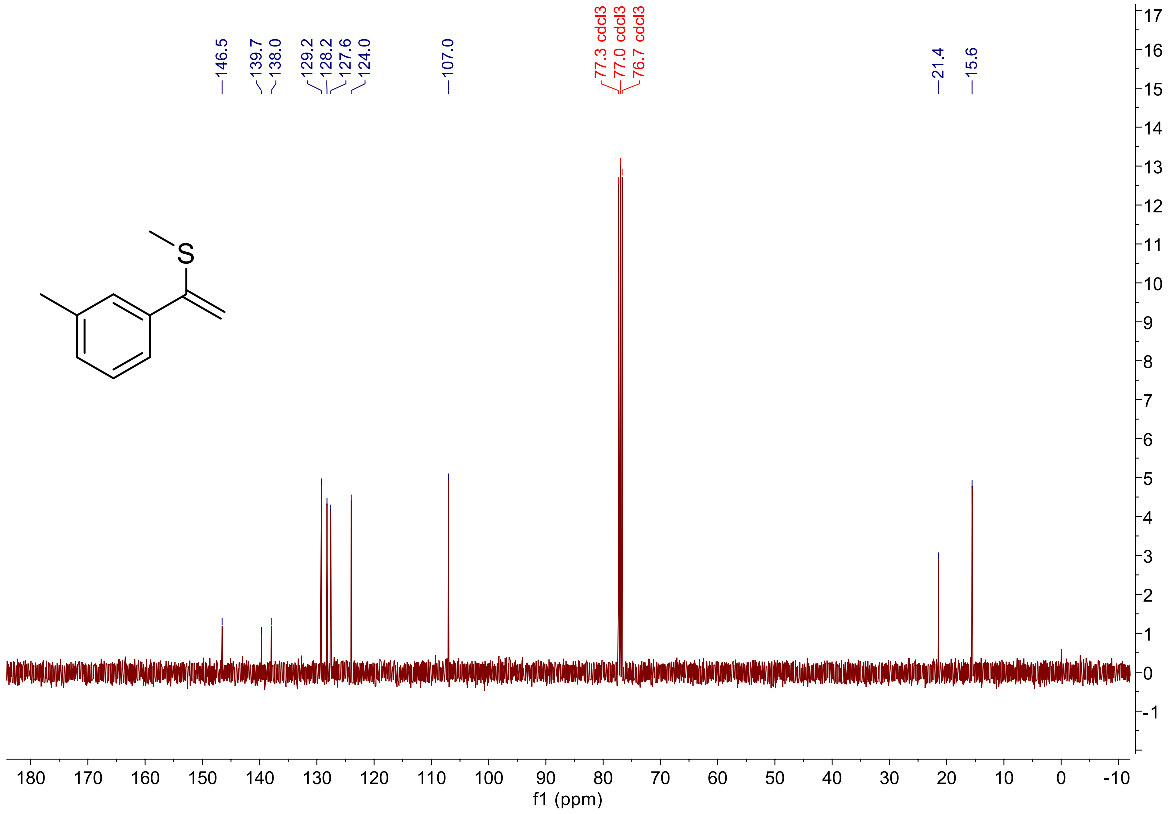

*Multiplicity-edited ^{1}H-^{13}C-HSQC NMR of **2d***

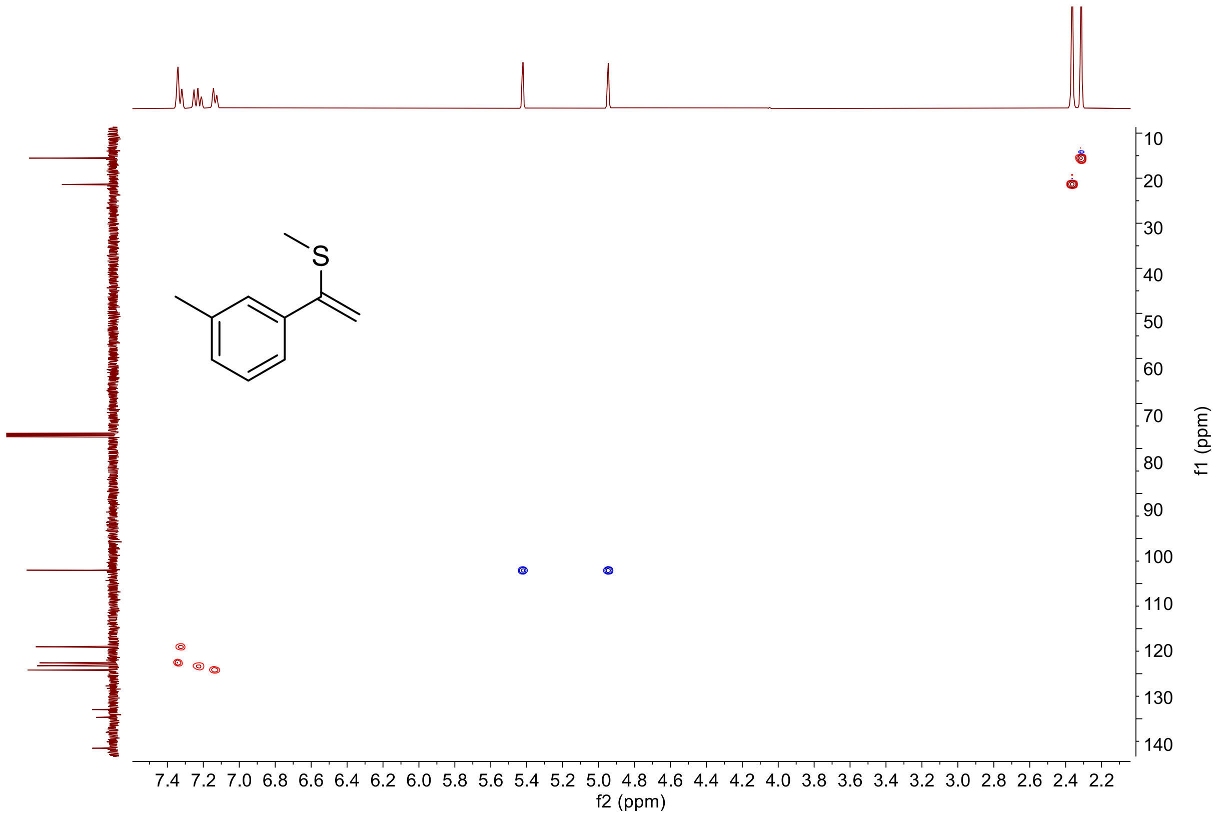

*^{1}H-NMR of **2e***

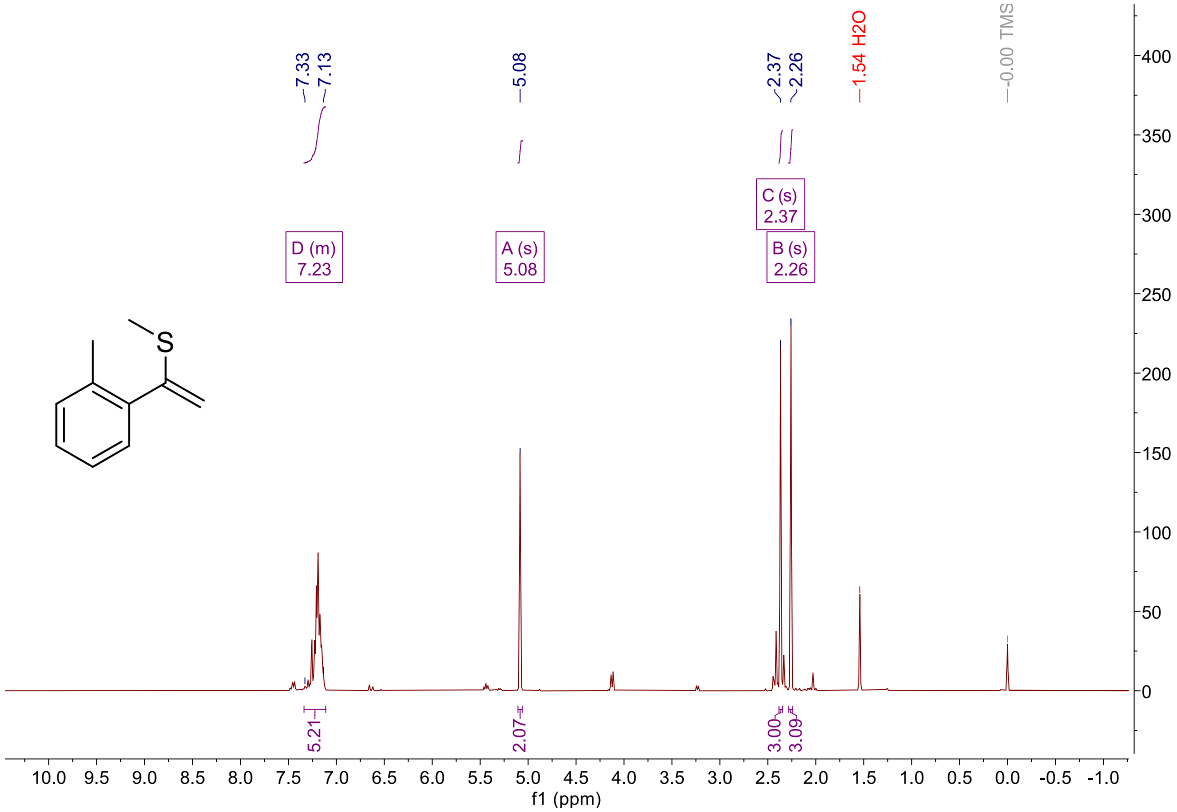

*^{13}C{^{1}H}-NMR of **2e***

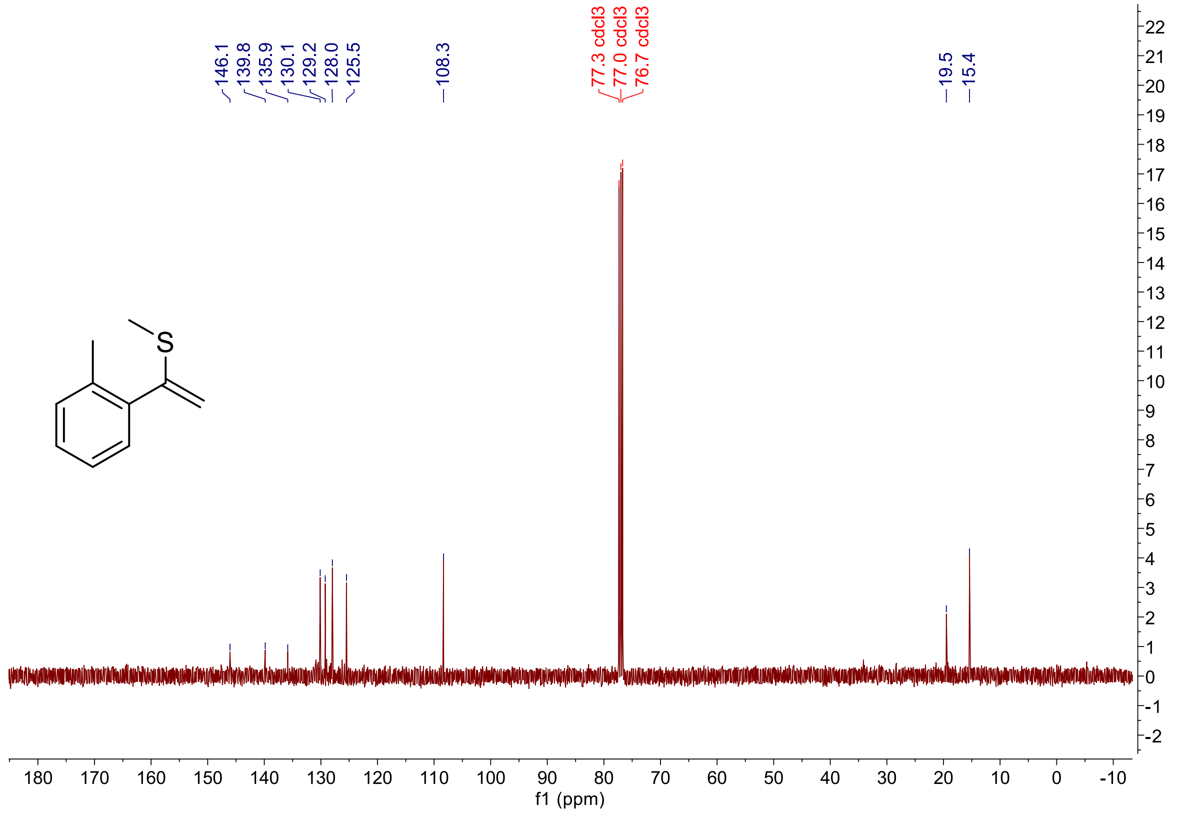

*Multiplicity-edited ^{1}H-^{13}C-HSQC NMR of **2e***

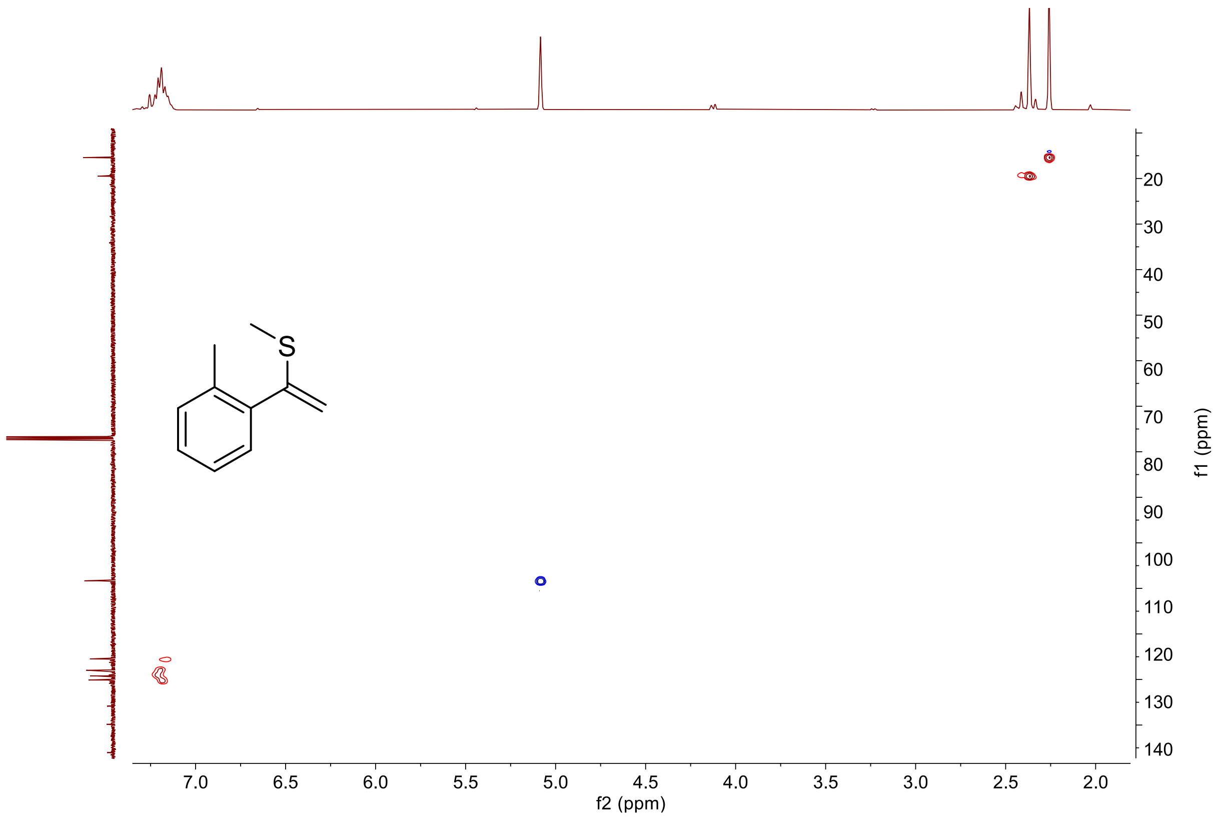

*^{1}H-NMR of **2f***

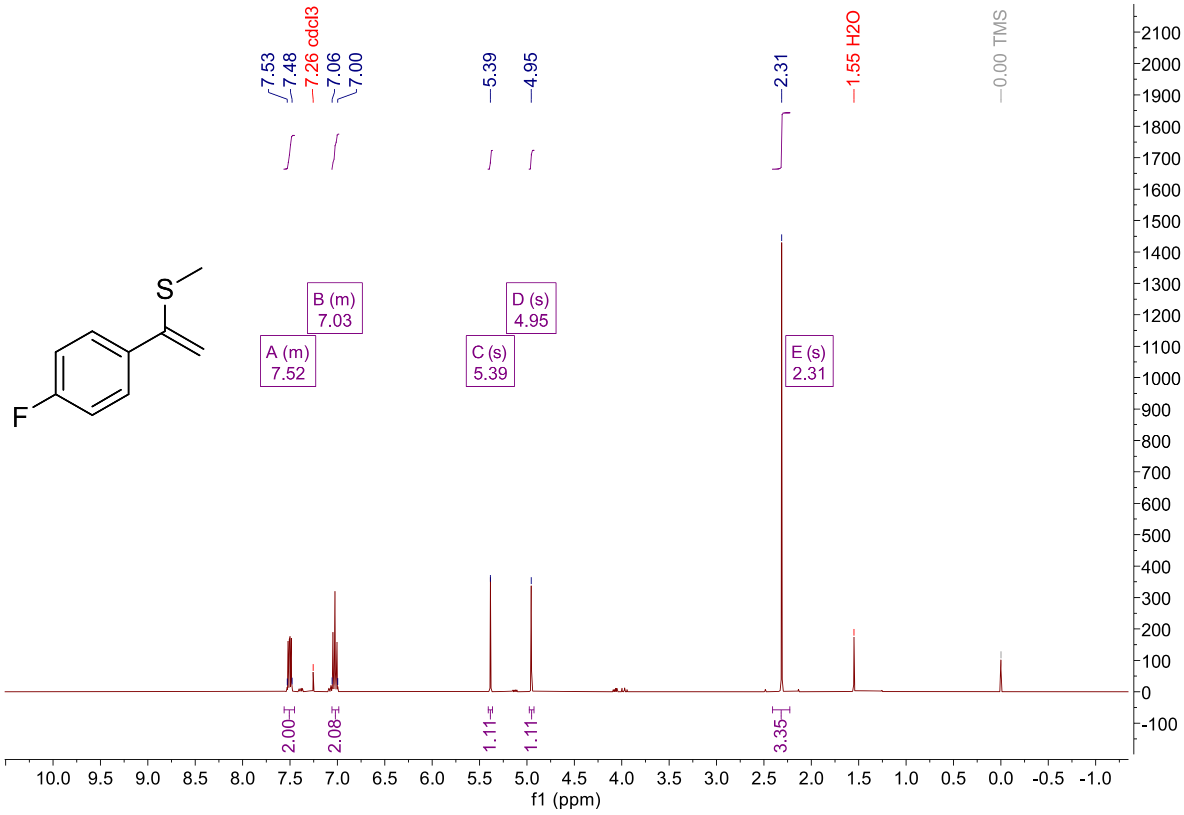

$^{13}C\{^{1}H\}$-NMR of **2f**

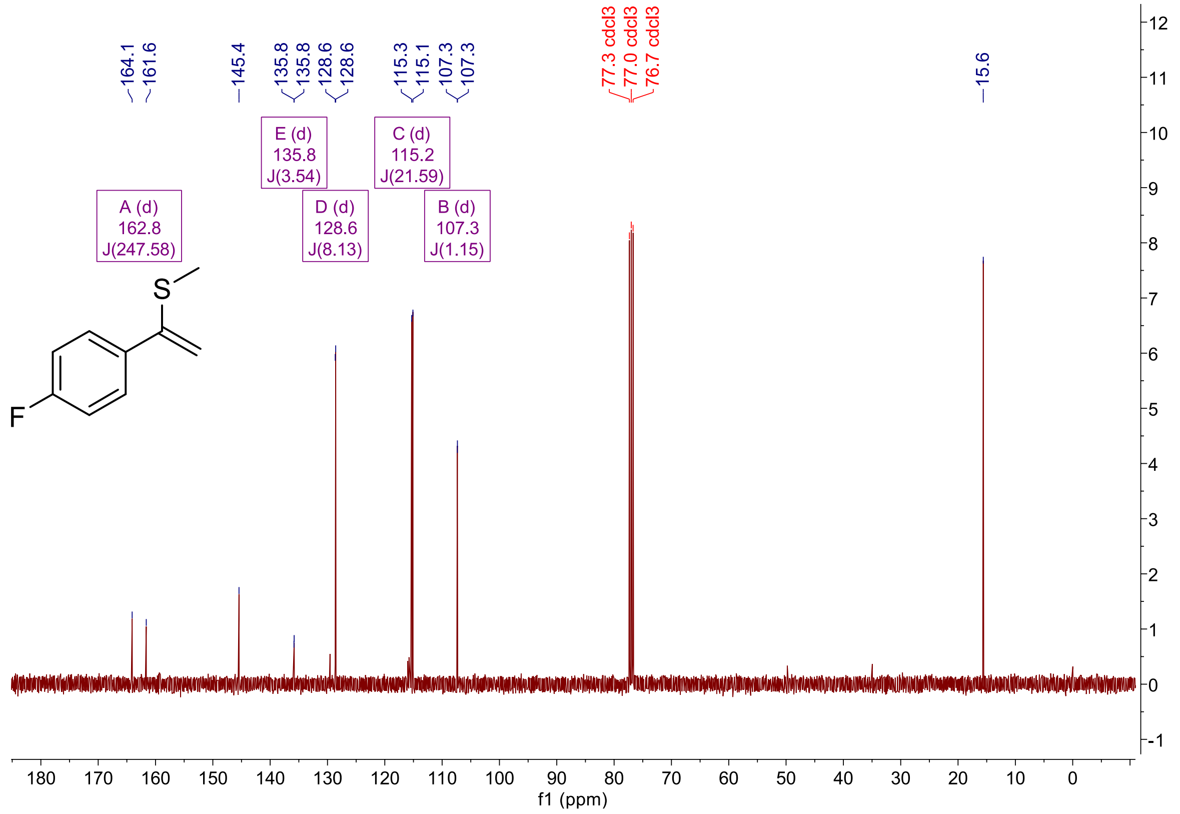

Multiplicity-edited ^{1}H-^{13}C-HSQC NMR of **2f**

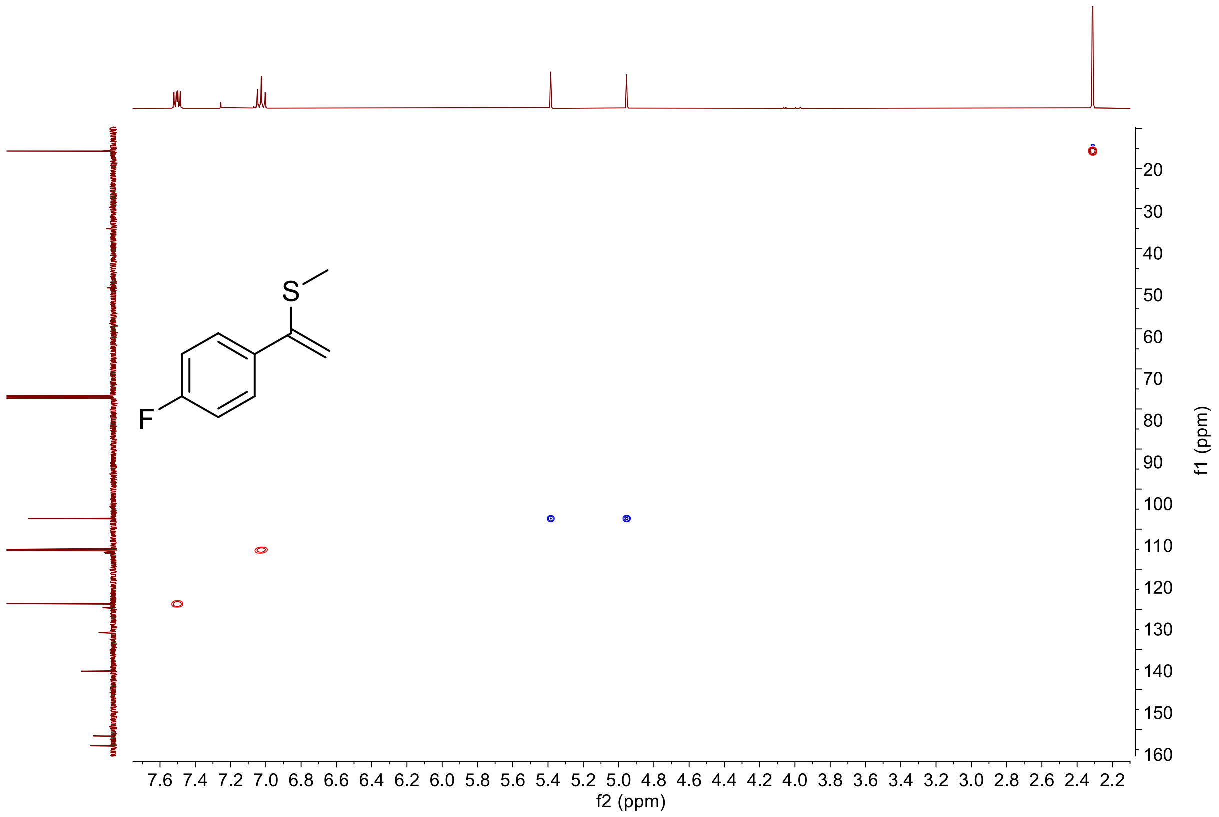

*^{1}H-NMR of **2h***

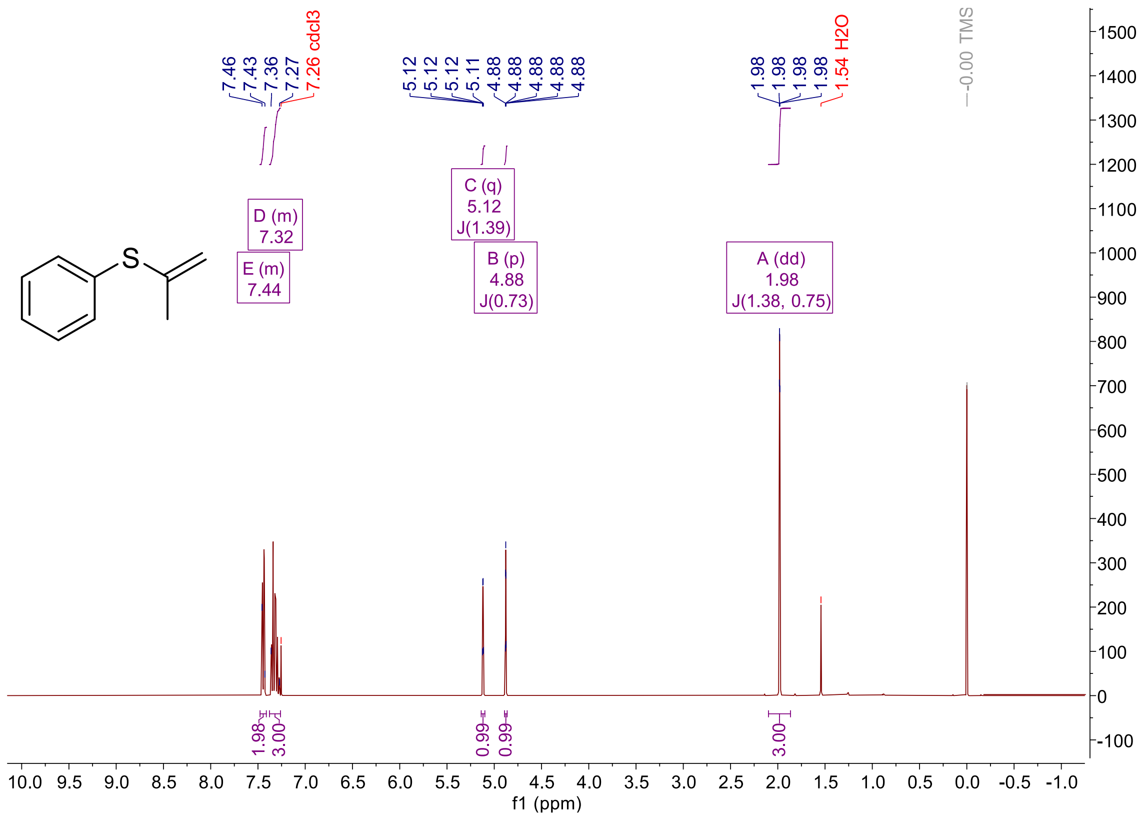

*$^{13}C\{^{1}H\}$-NMR of **2h***

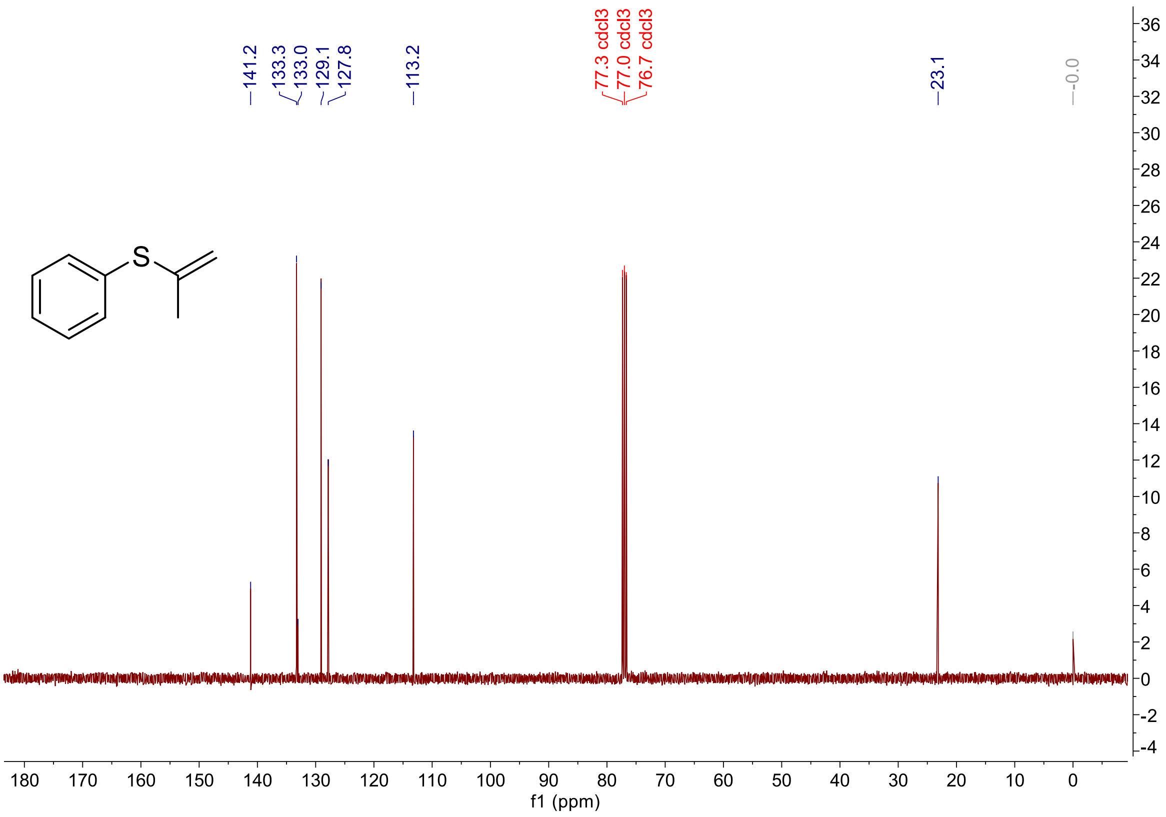

*Multiplicity-edited ¹H-¹³C-HSQC NMR of **2h***

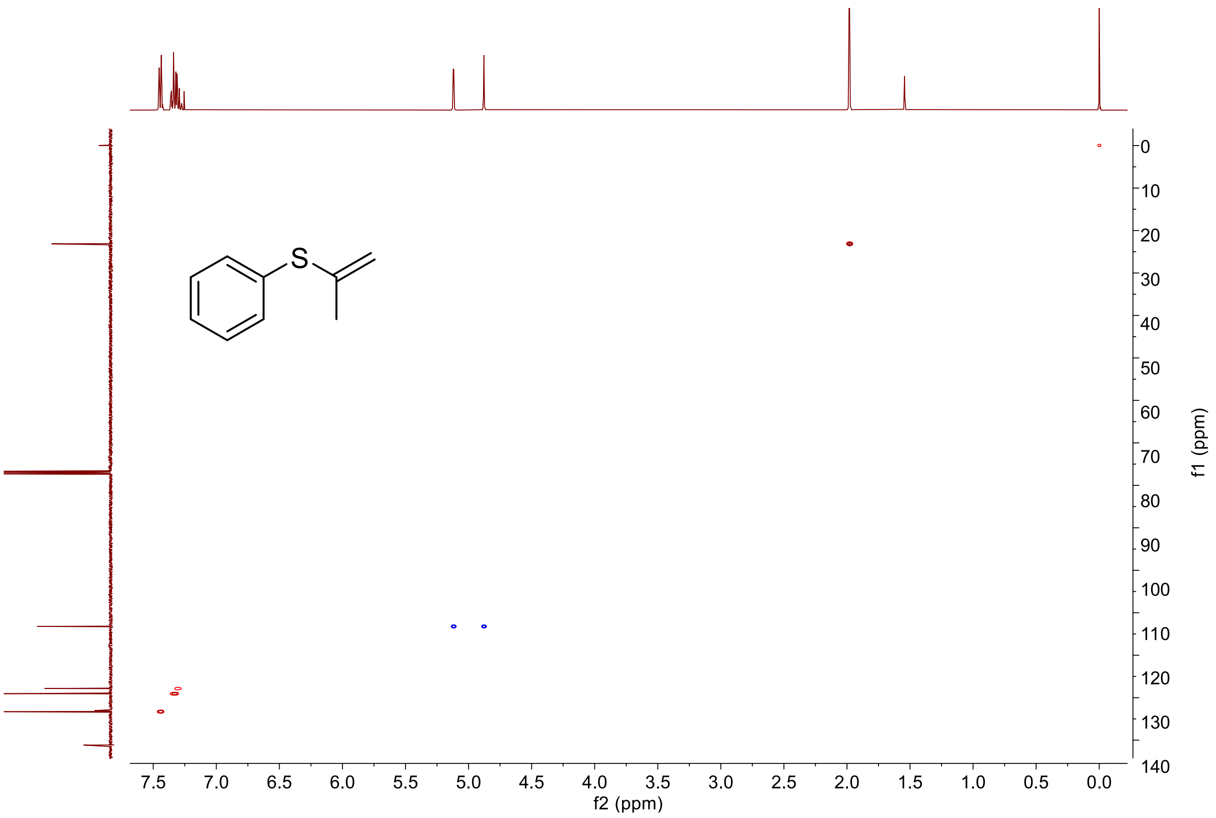

*¹H-NMR of **3aa** (produced with NCR)*

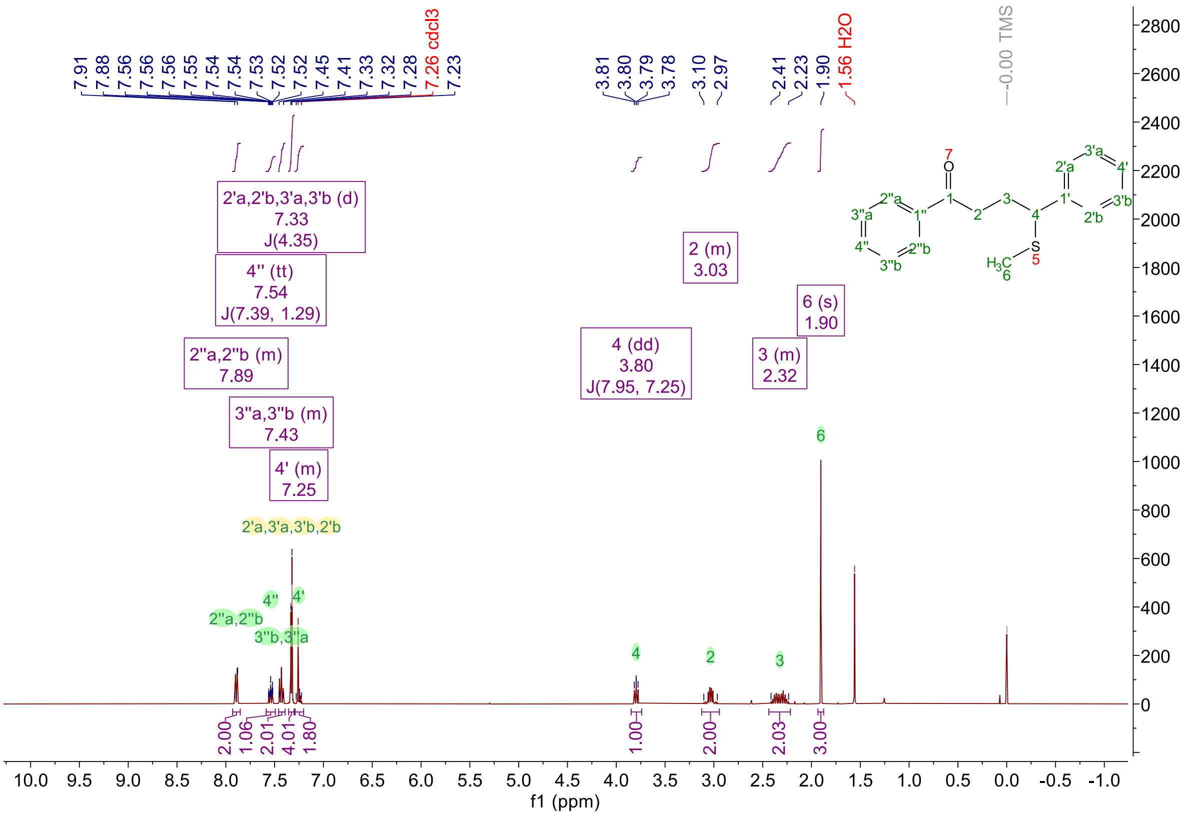

*^{13}C{^{1}H}-NMR of **3aa** (produced with NCR)*

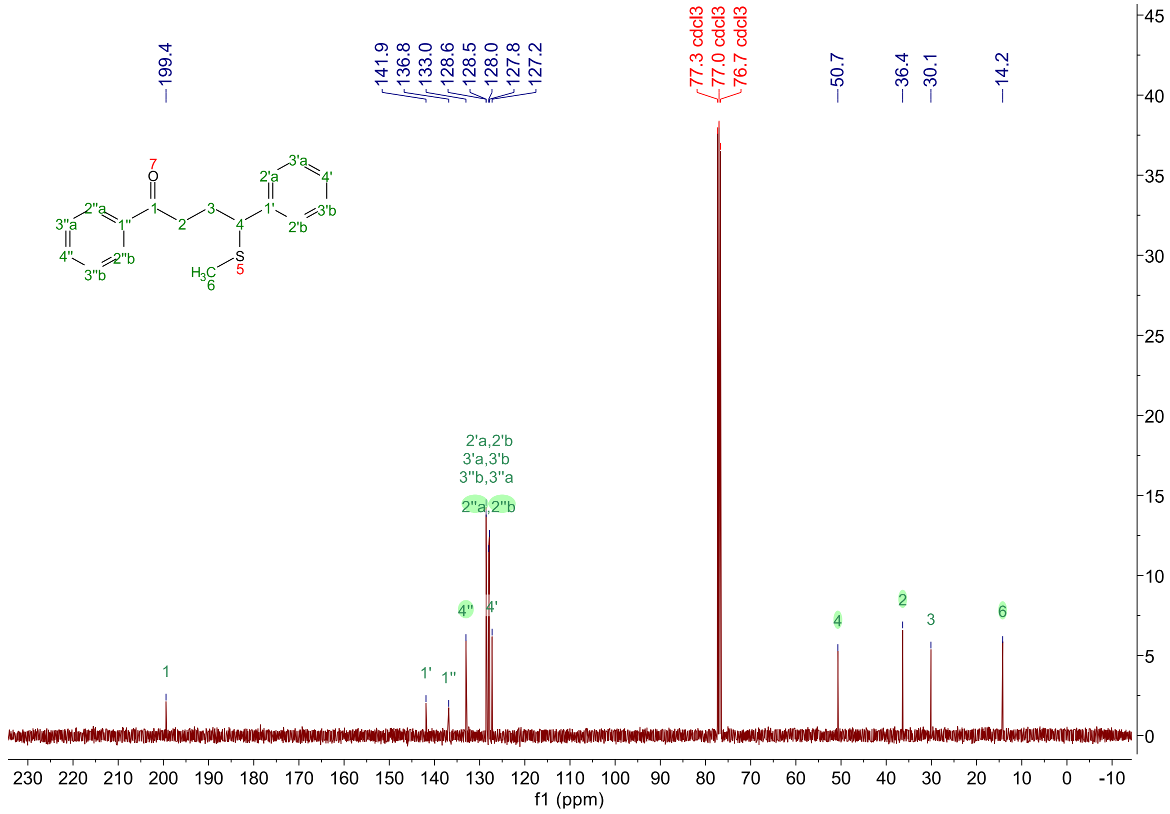

*^{1}H-^{1}H-COSY NMR of **3aa** (produced with NCR)*

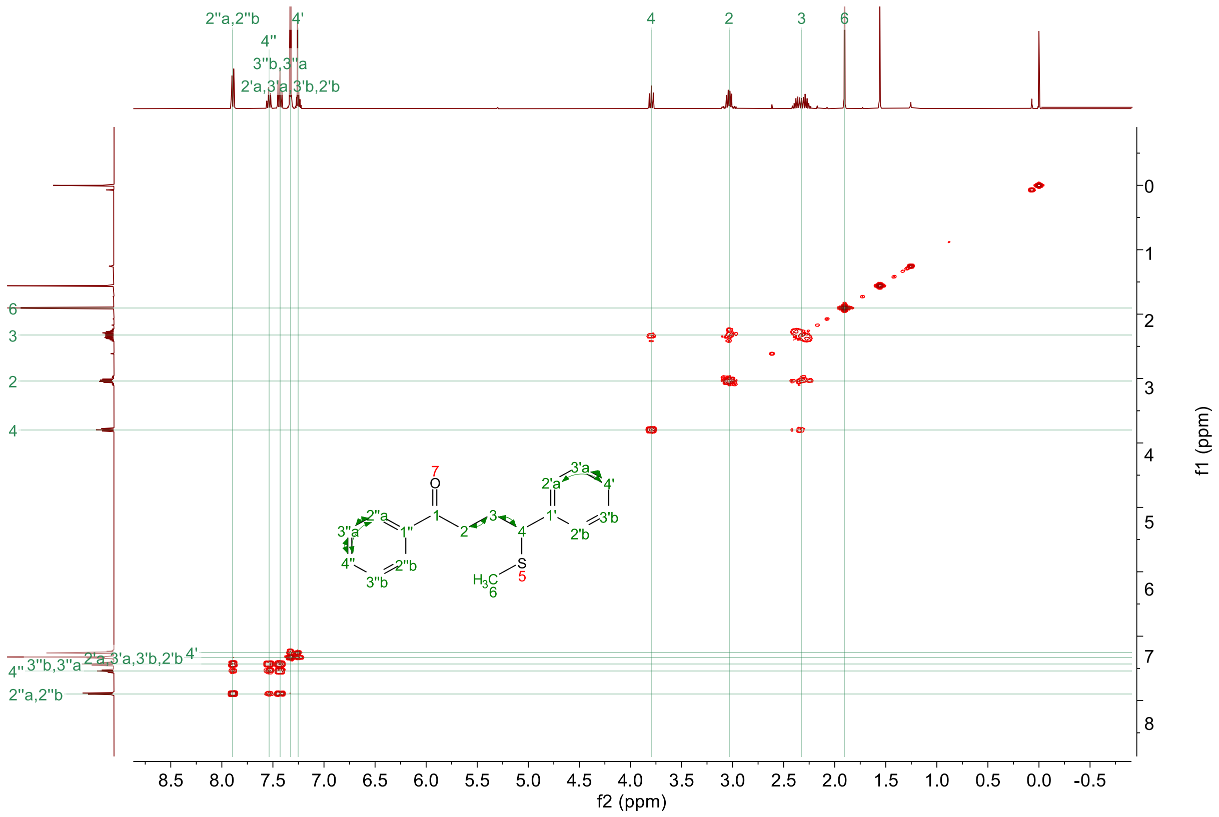

*Multiplicity-edited ^{1}H-^{13}C-HSQC NMR of **3aa** (produced with NCR)*

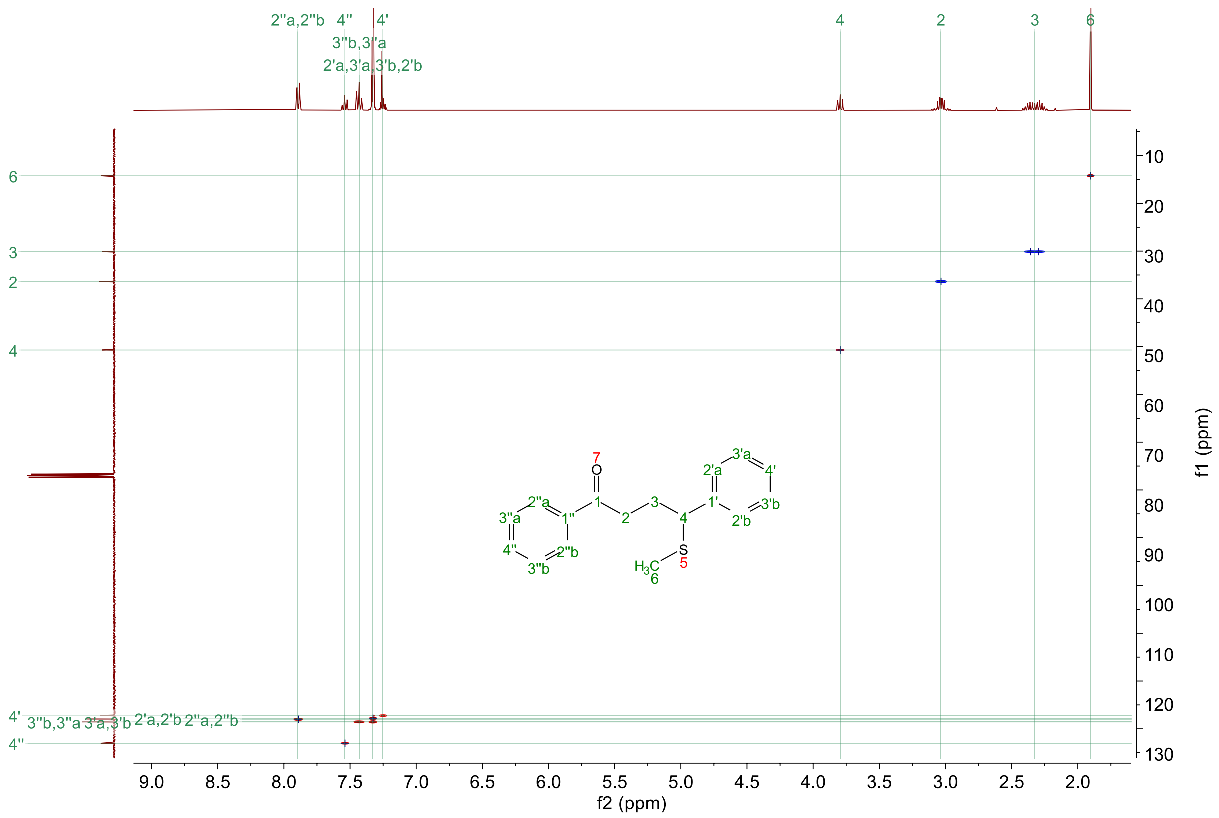

*^{1}H-^{13}C-HMBC NMR of **3aa** (produced with NCR)*

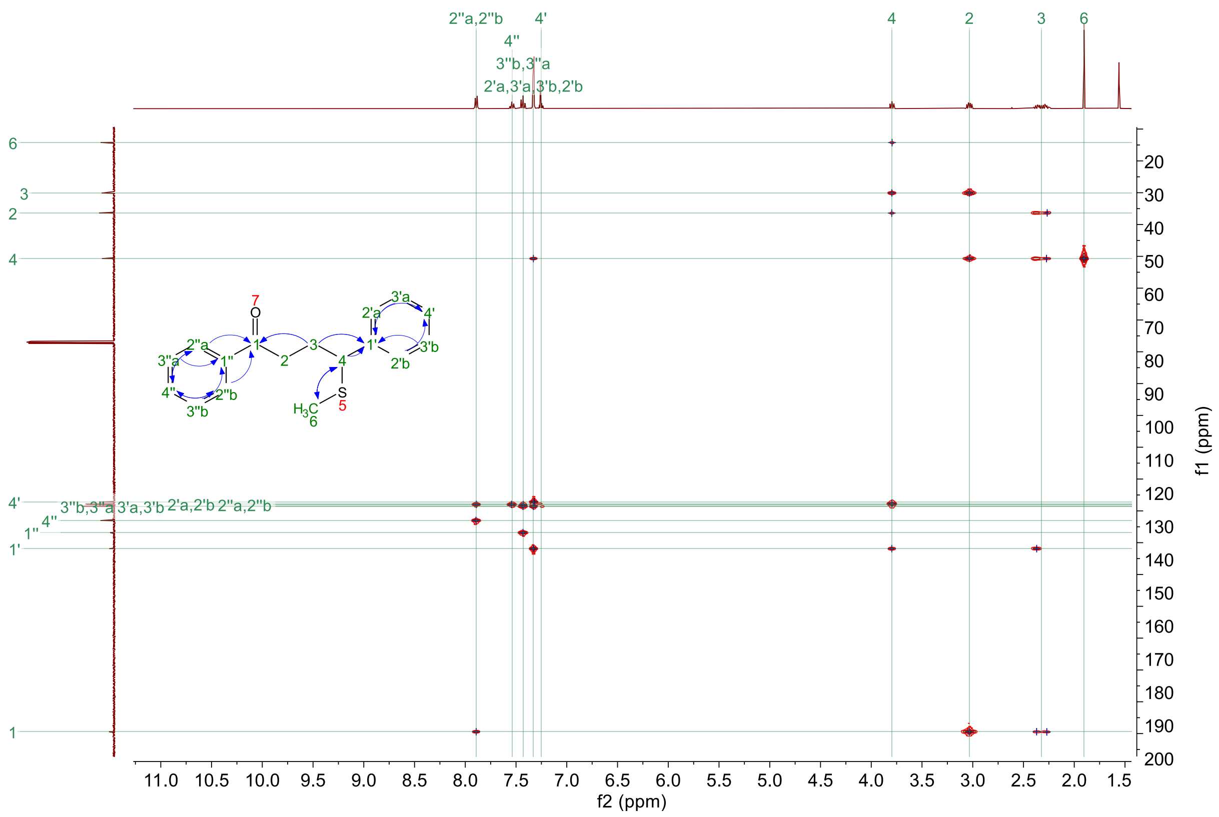

*^{1}H-NMR of **3aa** (produced with GluER T36A)*

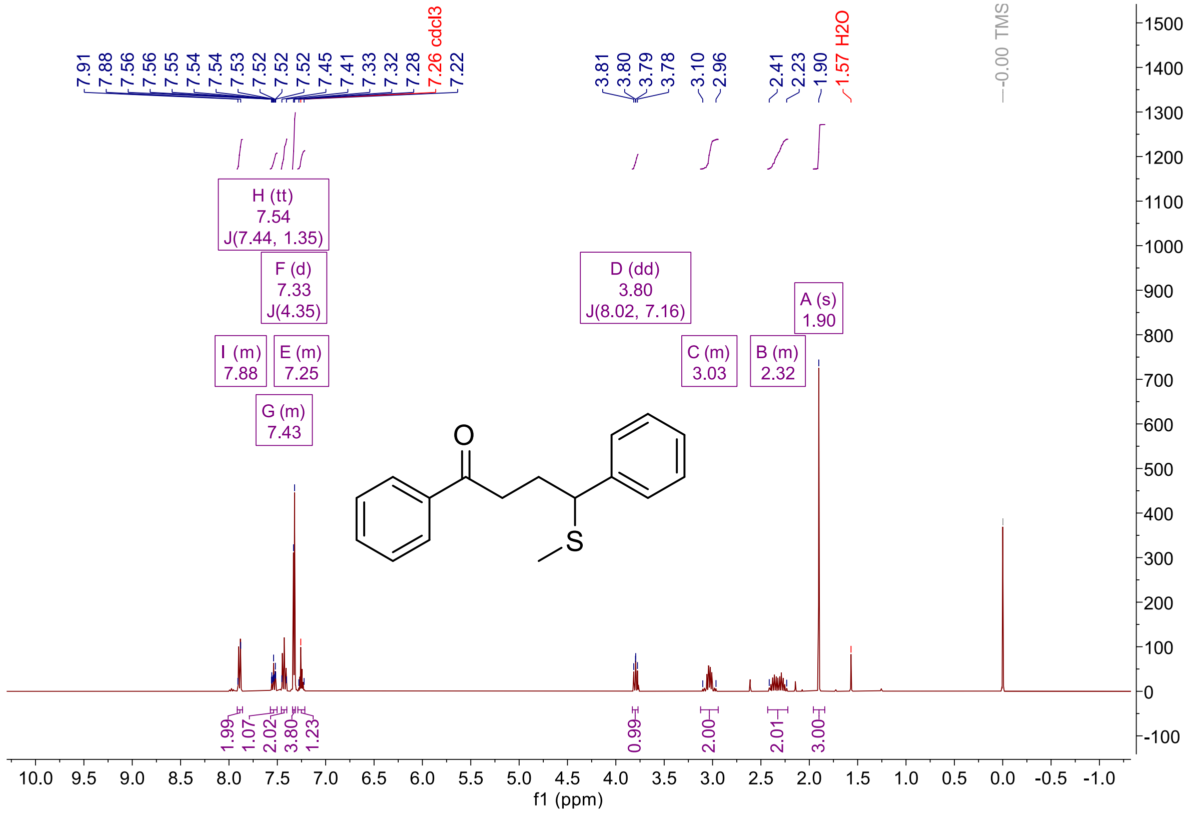

*^{13}C{^{1}H}-NMR of **3aa** (produced with GluER T36A)*

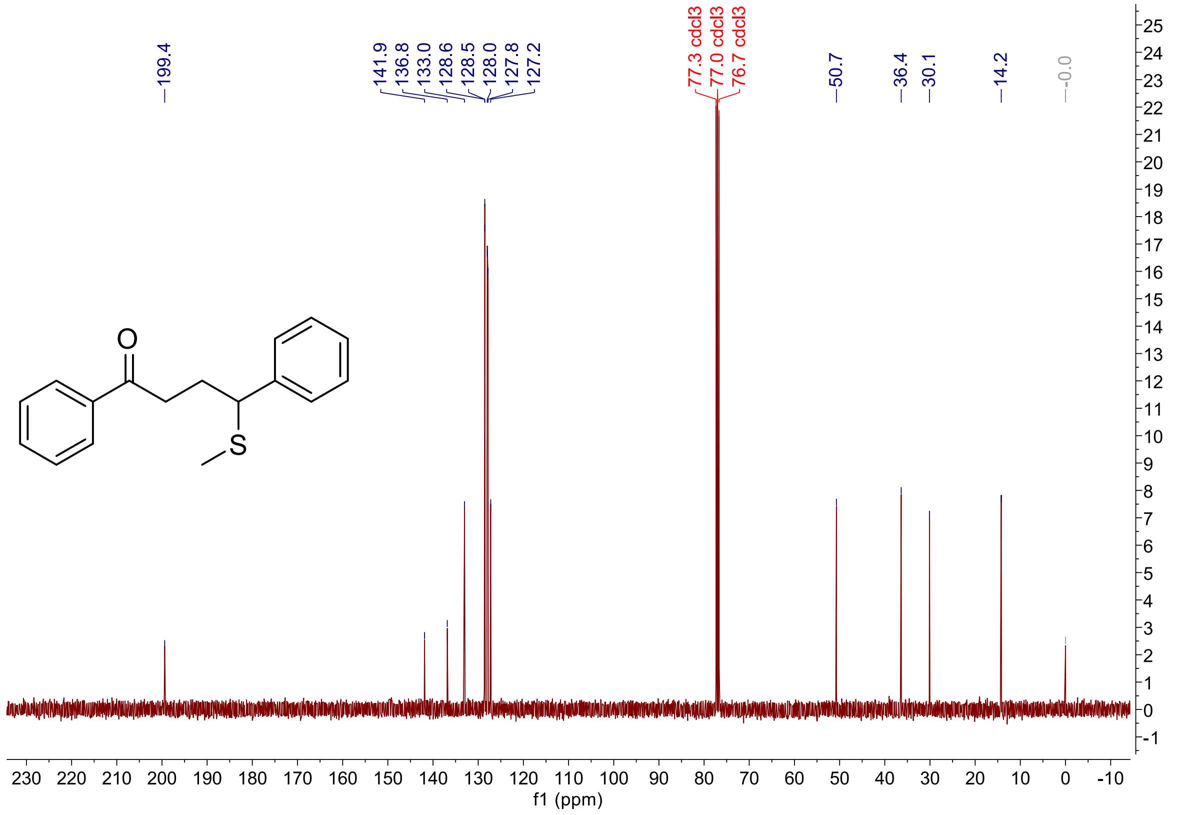

*^{1}H-NMR of GluER T36A catalyzed reaction of **1a** and **2a** in D_2O*

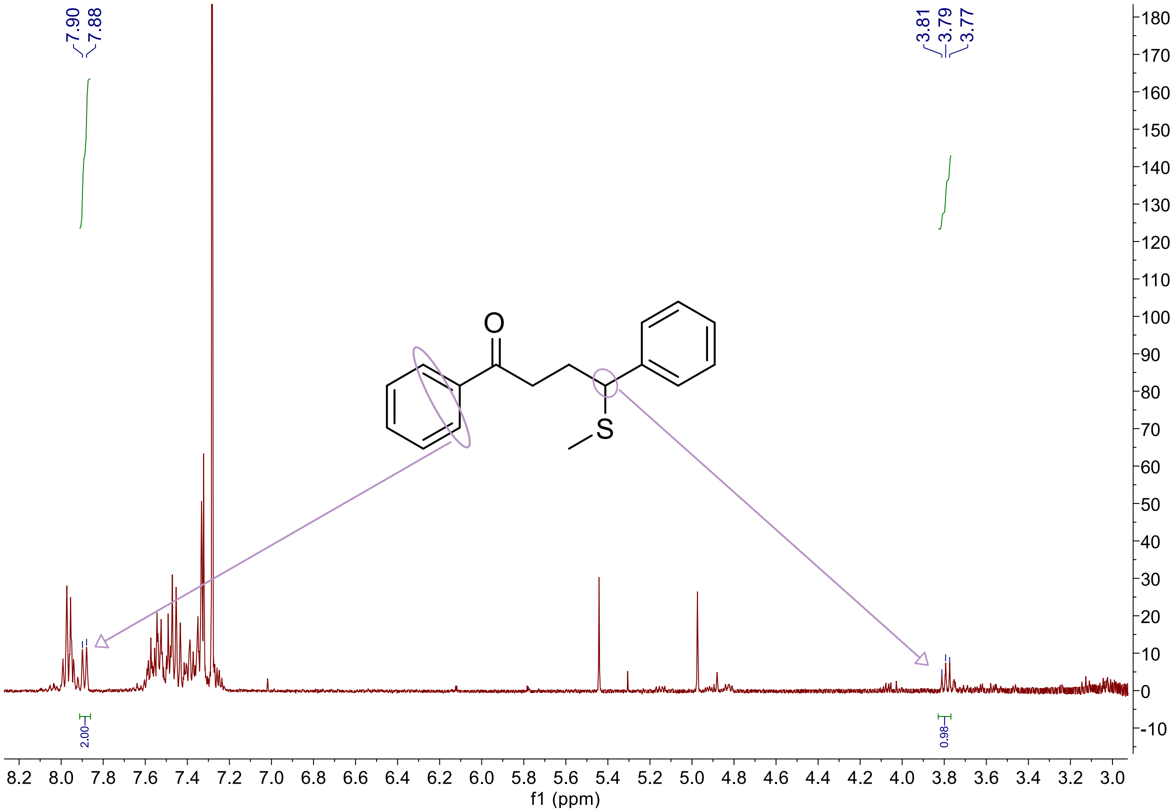

*^{1}H-NMR of GluER T36A catalyzed reaction of **1a** and **2a** using D-glucose-1-d_1*

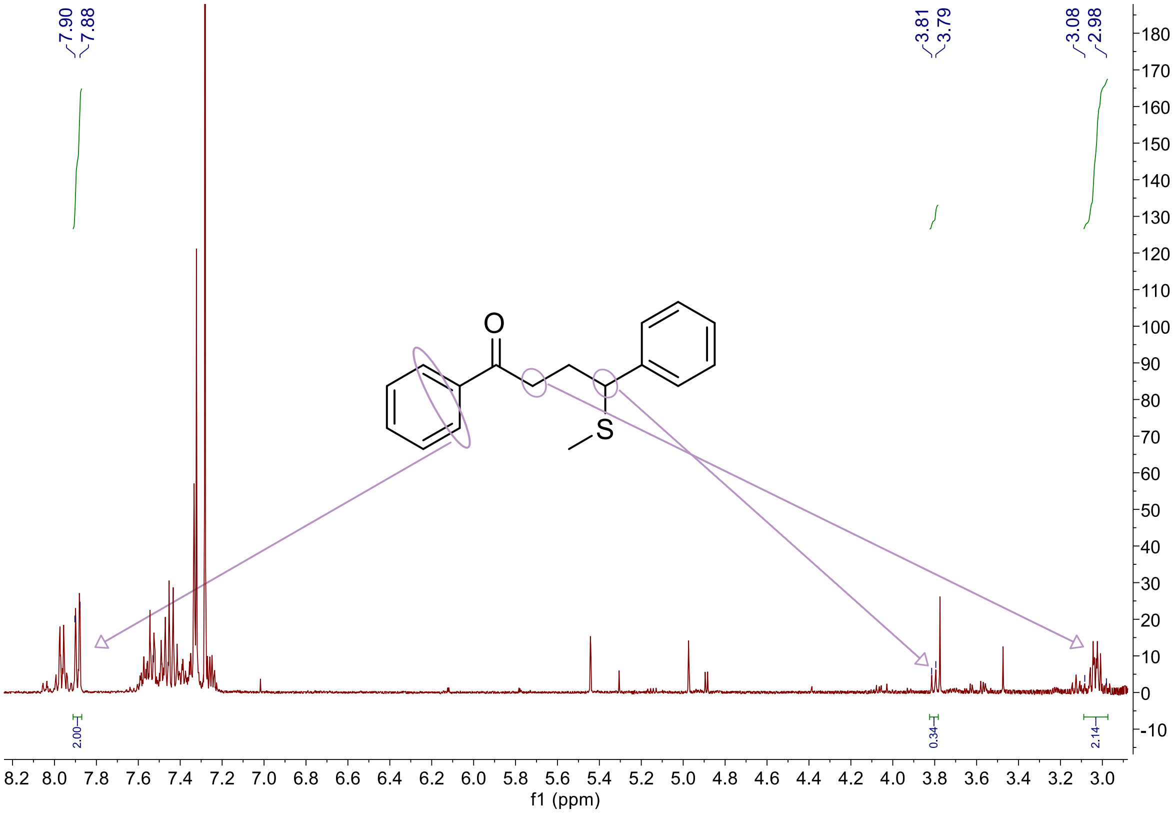

*^{1}H-NMR of **3ha** (produced with GluER T36A)*

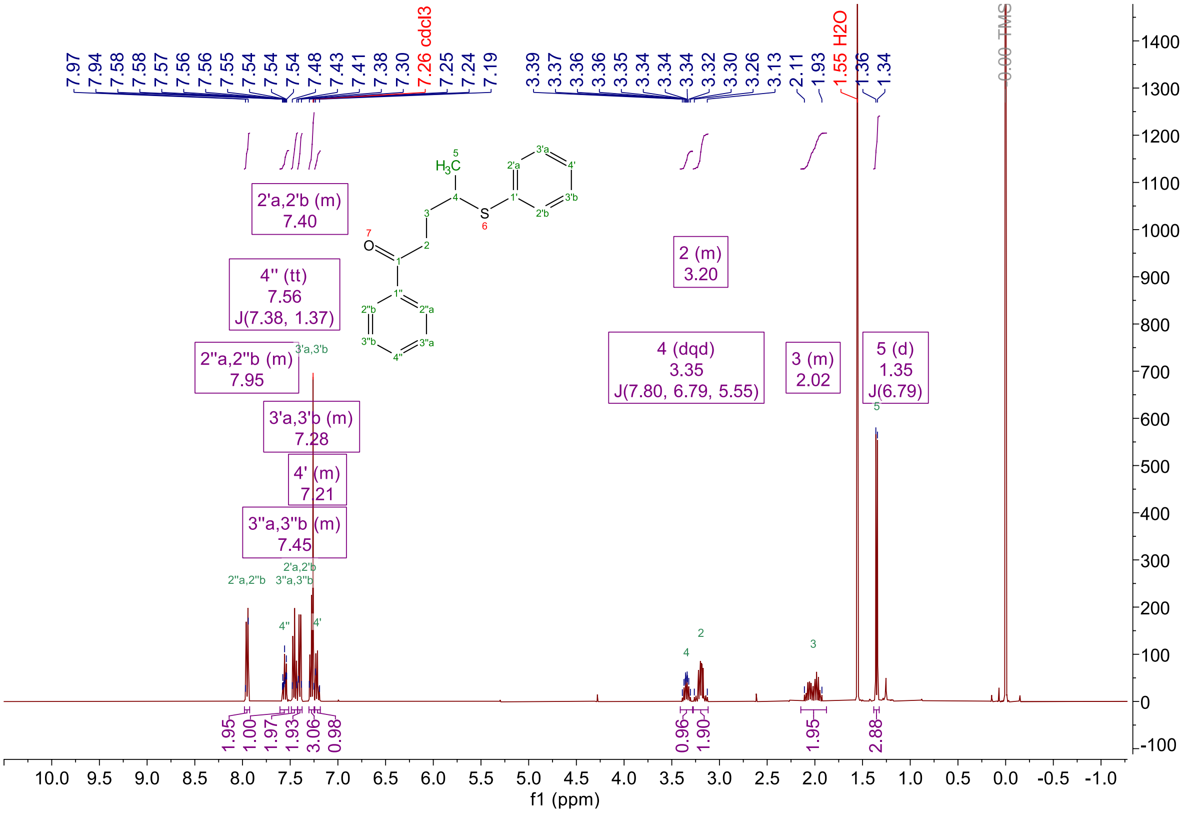

*^{13}C{^{1}H}-NMR of **3ha** (produced with GluER T36A)*

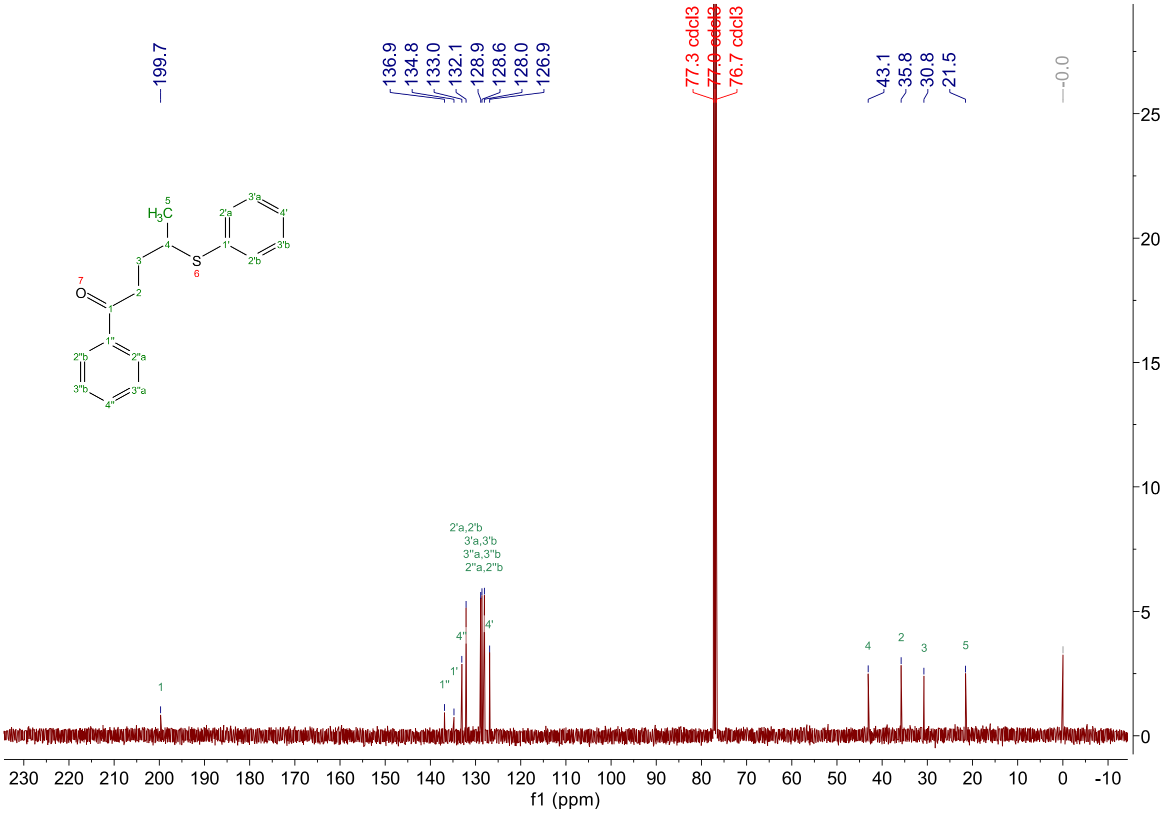

*^{1}H-^{1}H-COSY NMR of **3ha** (produced with GluER T36A)*

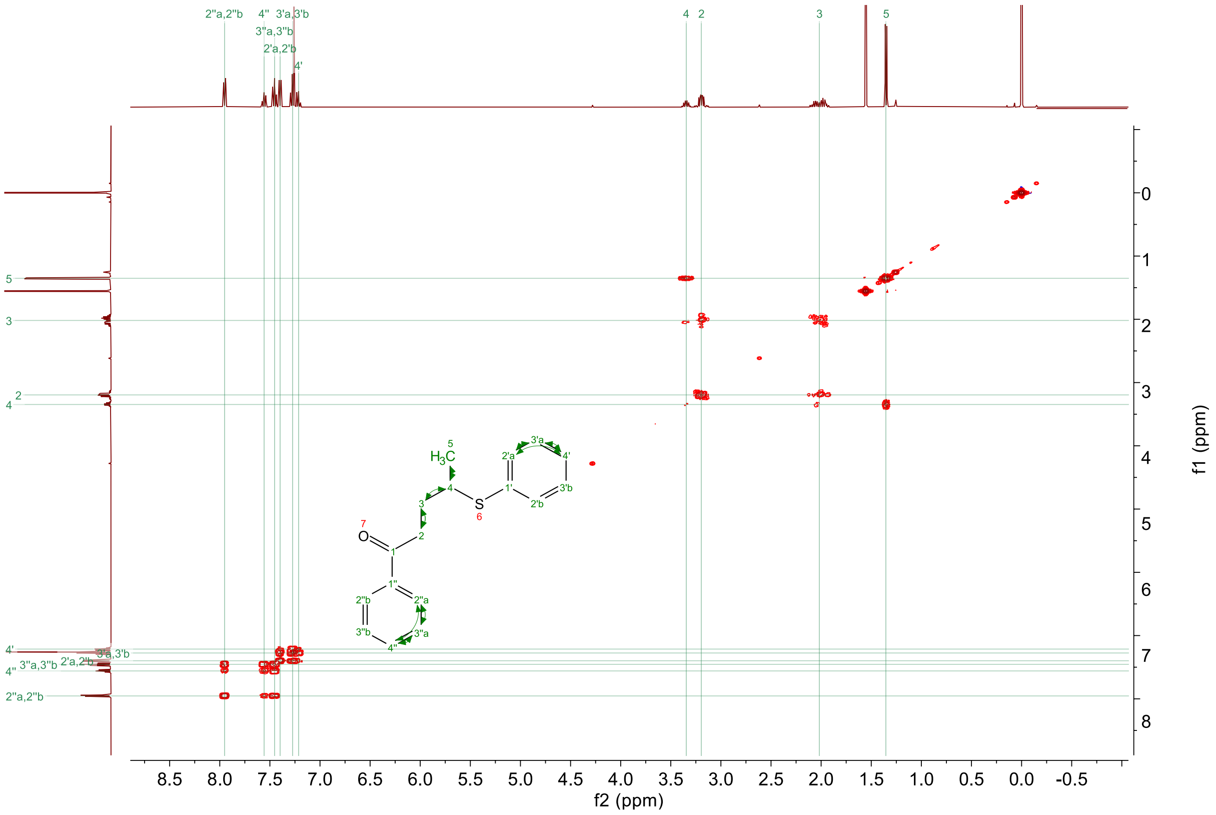

*Multiplicity-edited ^{1}H-^{13}C-HSQC NMR of **3ha** (produced with GluER T36A)*

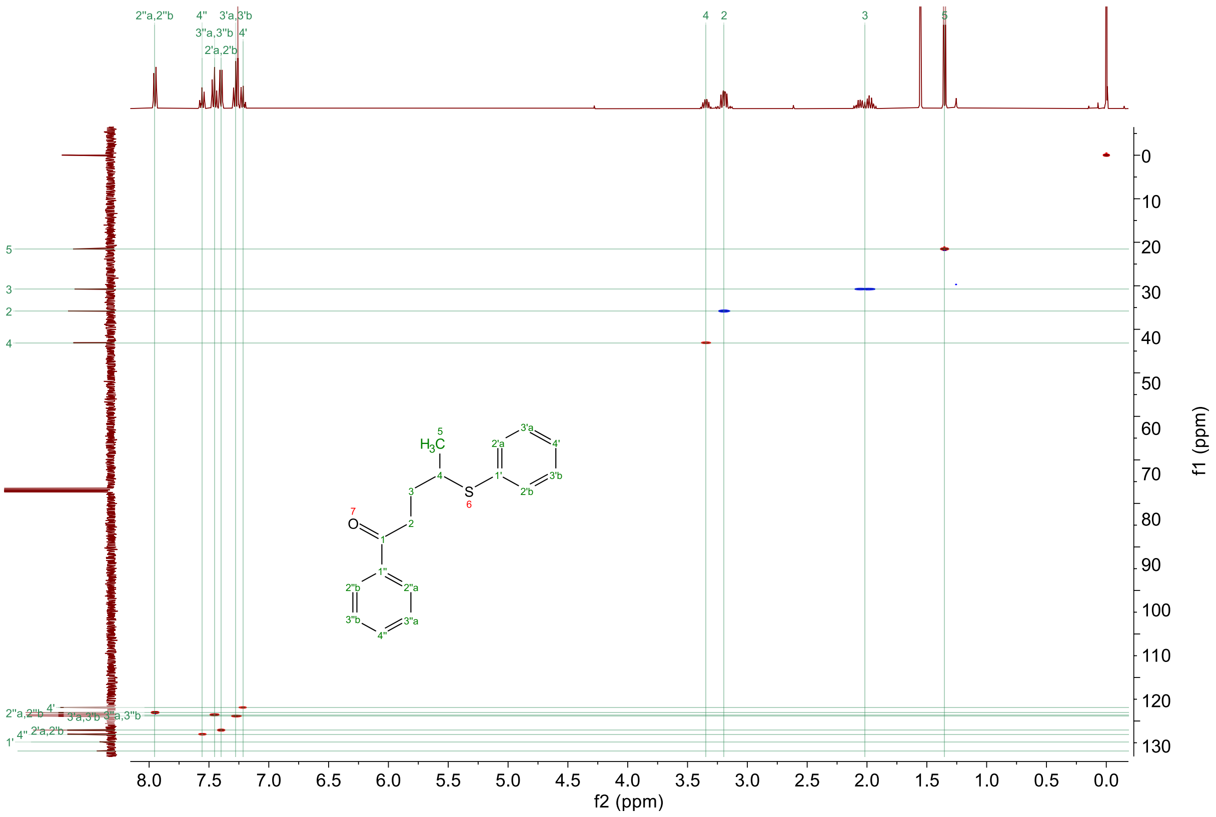

*^{1}H-^{13}C-HMBC NMR of **3ha** (produced with GluER T36A)*

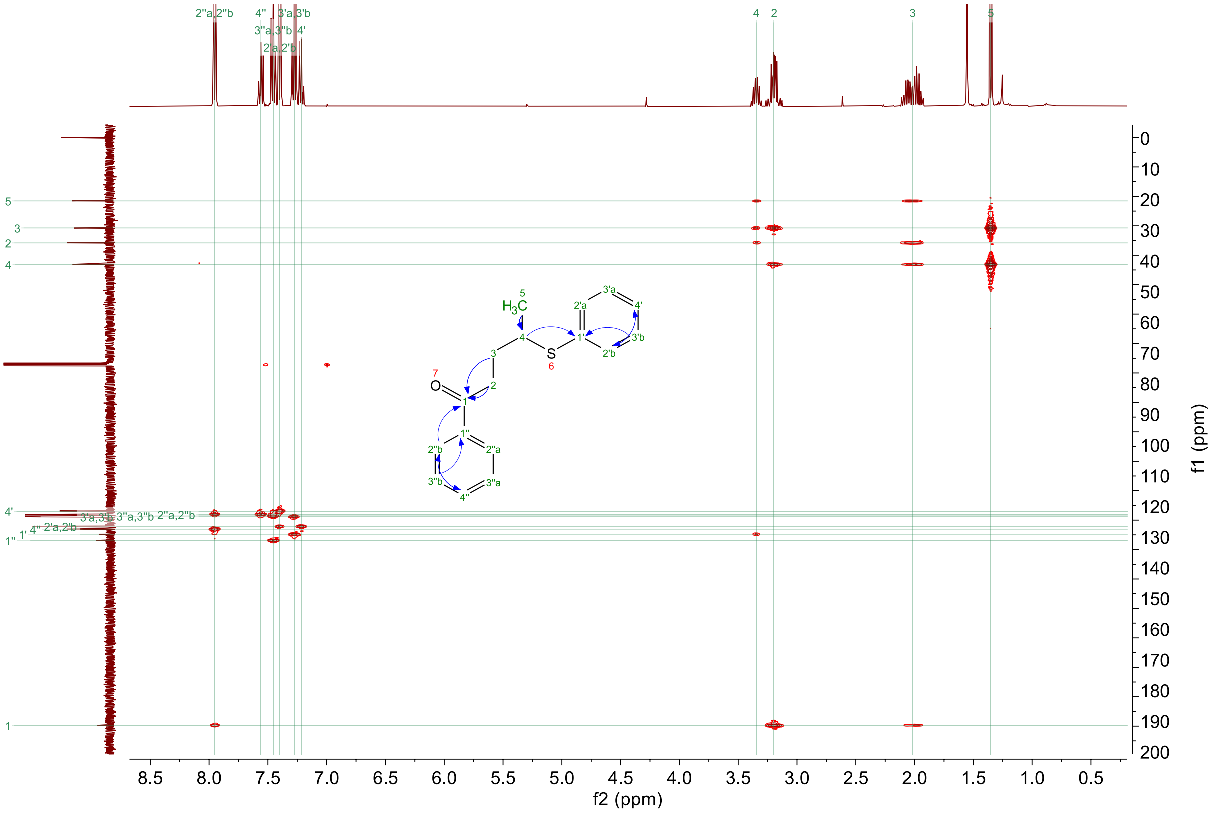

Supplement: Supplementary file 1 [file ja5c00761_si_001.pdf]
